# Biolipid Film‐Fused Electrochemiluminescence for Multipurpose In Situ Bioassays

**DOI:** 10.1002/advs.202524242

**Published:** 2026-04-07

**Authors:** Jialiang Chen, Bin Li, Yingying Wang, Fulin Zhu, Wenxuan Yang, Xuanyu Fu, Tiantian Man, Qiuyue Wu, Kewei Ren, Shengyuan Deng

**Affiliations:** ^1^ School of Environmental and Biological Engineering Nanjing University of Science and Technology Nanjing China; ^2^ School of Mechanical Engineering Nanjing University of Science and Technology Nanjing China; ^3^ School of Chemistry and Chemical Engineering Nanjing University of Science and Technology Nanjing China

**Keywords:** bilayer‐bridged biomembrane mimics, channel‐confined cytomembrane chemistry, electrochemiluminescence‐enabled biointerfaces, surface‐state‐sensitive signal transduction, vesicle‐merged single‐cell visualization

## Abstract

Electrochemiluminescence (ECL) is intrinsically a surface‐state‐sensitive strategy, yet its seamless synergy with soft biomembranes remains sparsely explored. Herein, a biolipid‐bound, membrane‐interactive scaffold is built upon an ECL‐emissive artificial nanochannel and smoothly blended into supported phospholipid bilayers containing cholesterol and diversified natural or synthetic lipids. Such bilayer‐bridged assemblies preserve efficient emission while permitting dynamic membrane remodeling. The resulting biointerface enables label‐free profiling of cytomembrane‐active species, including divalent cations, amphiphilic pharmaceuticals, and pore‐forming peptides, through real‐time isotherm and kinetic analysis. By further integrating phospholipid‐pendant recognition motifs, femtomolar detection of Alzheimer's disease biomarkers (β‐amyloid polypeptide and tau protein) is achieved. Moreover, vesicle‐mediated membrane merging embeds ECL emitters into living‐cell plasmalemma, affording surface‐confined single‐cell visualization using endogenous oxygen as the coreactant. Collectively, this biointerface‐compatible ECL paradigm enables multiplexed monitoring of molecular, membranous, and cellular processes via membrane‐structural modulation.

## Graphical abstract

An ECL‐emissive, membrane‐interactive scaffold was fabricated, and facilely fused with natural and non‐native phospholipids into multifactorial mimicries of cytomembranes and vesicles for in vitro representative membrane‐process probing. Such a biointerface‐based, state‐sensitive ECL paradigm not only pinpointed proximal phenomena, including channeling and chelation, but also served as a scalable substrate for multiplexed measurement, and was further merged onto living cells for efficient, eco‐friendly, surface‐confined imaging.

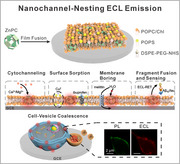

## 1 Introduction

Electrochemiluminescence (ECL) is an intrinsically surface‐confined emission technique, which has emerged as a cutting‐edge tool for in situ biointerfacial investigation and electrode process analysis [1]. Endowed with a high signal‐to‐noise ratio and rich spatiotemporal modalities, ECL excels over conventional optical probing approaches in studying dynamic biointerfacial events [2]. Recent refinements in ECL technology include: **(i)** total internal reflection coupled with super‐resolution ECL microscopy (with a focal depth of 400 µm) for revealing heterogeneity in multisplanchnic textures [3]; **(ii)** single‐protein sensing via ruthenium‐complexed conjugators during T‐cell transformation, accompanied by topographic profiling of target transmembrane receptors [4, 5]; and **(iii)** pixelated photon‐counting for contouring cellular contacts with higher morphological resolution than immunofluorescence staining [5], which has been further applied to evaluate the effects of electrophotodynamic antibacteriotherapy and optogenetic manipulation on *Drosophila larvae* [6, 7].

Despite these remarkable achievements, a core contradiction continues in applying ECL to biomembrane studies: the requirement for concentrated cytotoxic coreactants in traditional ECL systems is incompatible with physiological conditions [8]. To avoid coreactant‐induced cell necrosis, cell inactivation or paraformaldehyde fixation is commonly used as an expedient [8]; however, this approach only enables the study of static, shape‐persistent cytomembranes, and cannot reflect the intrinsic fluidity and dynamic processes of native biomembranes. A key research gap thus emerges: there is a lack of a membrane‐mimetic ECL platform that can preserve the fluidity of lipid bilayers while localizing ECL luminophores within the membrane phase rather than in the bulk solution. As an alternative strategy, endogenous reactive oxygen species (ROS) in cellular metabolons have been exploited as ECL coreactants to irradiate the cytosolic periphery [9]; yet, invasive conductive capillaries used in this method can hardly characterize the structure of (sub)organelles [10], nor can they achieve full‐cell landscape imaging via rapid scanning.

Inspired by red‐blood‐cell wall debris‐reconstituted liposomes for long‐circulating controlled release [11], membrane‐fusing ECL systems have been developed (e.g., the Meso‐Scale‐Discovery (MSD) multifactorial ECL imager) for high‐throughput assays of antibody‐drug affinities [12]. Nevertheless, this in vitro model tends to form multilamellar fragment films, which bury binding sites in the interlayers and thus deviate significantly from the realistic physiological membrane microenvironment. Another critical gap is the precise probing of small molecules (e.g., ions, metabolites, and liposoluble drugs) proximal to compliant lipid bilayers: these species lack specific epitopes for ECL labeling and typically induce only subtle interfacial electrical perturbations, making it extremely difficult to transduce their interactions with biomembranes into robust and quantifiable ECL signals [13]. Notably, these small molecules are key participants in a variety of membrane‐associated physiological processes, including Cu^2+^‐coordinated recruitment of phosphatidylserine to K^+^ channels [14], adsorption‐primed transmembrane permeation of liposoluble ligands (e.g., ibuprofen) [15], and nanofiltration by cell‐penetrating peptide assemblies (e.g., penetratin and hirudin) [16, 17]. Therefore, it is urgent to develop ECL‐based strategies to interrogate these small molecule–membrane interactions, delineate their concentration‐responsive patterns, and extract the underlying biophysical properties–efforts that will complement well‐established biofilm‐based techniques such as TIRF microscopy and patch‐clamp platforms [3].

To circumvent the above limitations, Bard et al. engineered collisional ECL events at bare electrodes by applying overpotentials across gradients of attoliter‐scale unilamellar vesicles or oil droplets enriched with rubrene and organic amines [18]. However, these evanescent ECL signals rarely reflect stable interactions at lipoidal heterointerfaces [19, 20], and cannot realize quantitative characterization of membrane processes. Fortunately, nanopore sequencing platforms have achieved refined fingerprinting of homologs and enantiomers via their iontophoretic characteristics during traversal through polymer‐film‐fused porins [21]; this precedent provides a conceptual framework for ECL sensing of biomembrane processes. Specifically, the symport channeling of coreactants, cations, and other species across lipid bilayers is jointly governed by localized surface tension and tacticity [22], both of which are highly sensitive to the binding of biolayer‐specific substrates. This interdependence furnishes a unique basis for ECL‐oriented “bio‐surface” analysis: although the surface‐confined nature of ECL is often regarded as a drawback for bulk solution interrogation, it becomes a distinct advantage for membrane‐focused studies by strictly restricting the ECL readout to the lipid‐electrode interface with near‐zero background. By engineering a luminophore that thermodynamically embeds into the headgroup region of lipid bilayers, we can convert minute membrane binding/insertion events into high‐contrast, surface‐confined ECL signals, enabling quantitative readout of membrane perturbations that are inaccessible to conventional bulk electrochemical techniques.

**FIGURE 1:**
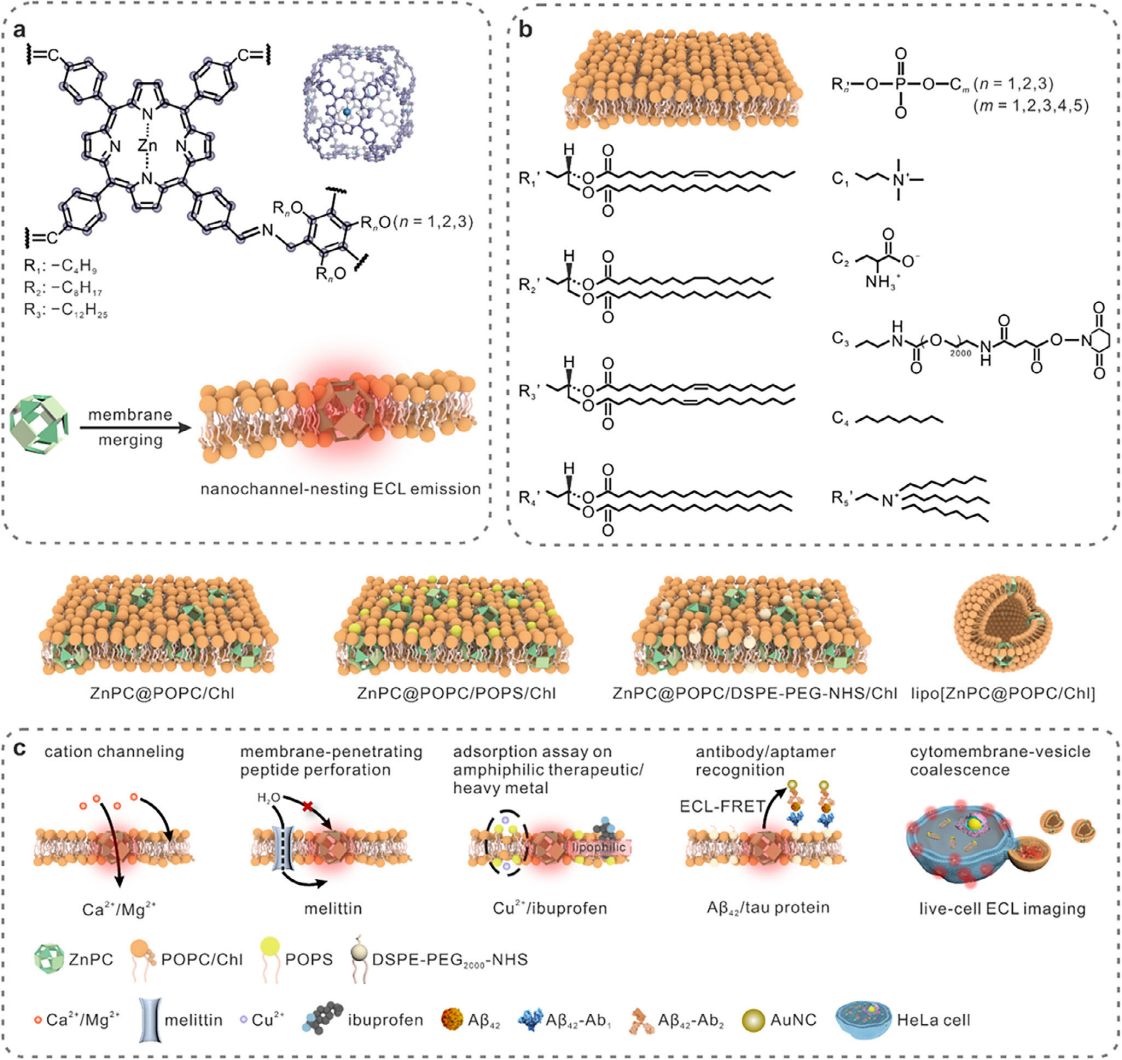
Overview on the nanochannel‐embedded membrane formation for pluripurpose probing via ECL emissions. (a) ChemDraw‐constructed connections between ZnTFPP and (2,4,6‐tri(butoxy/octyloxy/dodecyloxy)benzene‐1,3,5‐triyl)trimethanamines (^4/8/12^TbTm) through Schiff‐base bonding; the upper right depicts the topological polyhedron of a ZnPC channel composed of 6× face‐centred ZnTFPP and 8× tripodal TbTm (for concision and clarity, peripheral alkoxys, *R*_n_, are omitted). (b) SLB membrane modeling made of Chl and a batch of (a)biological lipids in a fundamental form as *R*_n_′O(O═P─O^−^)OC_m_. Below are specified structures of *R*_n_′ and *C*_m_, encompassing common categories (Table S1) of 3‐palmitoyl‐2‐oleoyl‐*sn*‐glycero‐1‐phosphoChline (POPC), 1‐palmitoyl‐2‐oleoyl‐*sn*‐glycero‐3‐phospho‐L‐serine (POPS), 1,2‐dipalmitoyl‐*sn*‐glycero‐3‐phosphocho‐line (DPPC), 1,2‐dipalmitoyl‐*sn*‐glycero‐3‐phospho‐L‐serine (DPPS), 1,2‐dioleoyl‐*sn*‐glycero‐3‐phospho‐L‐serine (DOPS), 1,2‐distearoyl‐*sn* ‐glycero‐3‐phosphoethanolamine – polyethylene glycol 2000 – *N*‐hydroxy succinimide (DSPE‐PEG‐NHS), as well as a synthetic surfactant, lipofectamine (2‐(trioctyl‐ammonio)ethyl)octylphosphate (TOAP). (c) Coalescing ZnPCs with lipids into ECL‐enabled bilayers, denoted (from left to right) as ZnPC@POPC/Chl, ZnPC@POPC/POPS/Chl, ZnPC@POPC/DSPE‐PEG‐NHS/Chl, together with a unilamellar liposome lipo[ZnPC@POPC/Chl]. The four afford five analytical avenues (from left to right): (1) Mg^2+^/Ca^2+^ channeling‐caused ECL extinguishment, (2) Cu^2+^/ibuprofen‐binding‐induced ECL variation, (3) melittin‐insertion‐induced ECL change, (4) antibody/aptameric recognition‐rendered ECL annihilation amplified by energy transfer to an Au nanotag, and (5) whole‐cell imaging irradiated by in‐membrane ECL without compromising permeability.

Building on our previous work on the transmembrane tunneling behavior of halide anions resolved from potentiostatic ECL decay profiles [22], we herein fabricate a biolipid‐fused ECL construct by emulating the thylakoid membrane (an organic optoelectronic transducer where chlorophyll analogues slip‐stack with multi‐ion channelosomes into caveolae‐like assemblies) [24]. Specifically, an ECL‐emissive artificial ion channel (denoted ZnPC) was synthesized via dynamic covalent chemistry [25], forming a rhombicuboctahedral nanocage composed of 6× zinc *meso*‐tetra(*p*‐formylphenyl)porphine (ZnTFPP) chromogenic cofactors and 8× lipophilic triamino moieties (Figure 1a). As an open‐channel architecture, ZnPC undergoes shallow insertion into lipid bilayers, preferentially localizing in the headgroup region to maximize interfacial interactions with cholesterol (Chl), 1‐palmitoyl‐2‐oleoyl‐*sn*‐glycero‐3‐phosphocholine (POPC), and other functional lipids (Figure 1b). The resulting ZnPC@POPC/Chl assembly can be easily immobilized on electrodes, exhibiting robust ECL efficiency, reproducibility, and favorable antifouling performance.

By incorporating phosphatidylserine (POPS) at physiologically relevant concentrations, we assembled an auto‐ECL cytomembrane mimic that enables high‐fidelity, label‐free affinity assays of multilevel model ligands (divalent cations Mg^2+^/Ca^2+^, ibuprofen, and melittin) (Figure 1c). Furthermore, this universal, uniform, and facile film‐forming strategy was adapted into an immunosensing substrate by co‐blending pegylated phospho‐ethanolamine (DSPE‐PEG‐NHS) as a coupling mediator, achieving ultra‐sensitive detection of two Alzheimer's disease biomarkers: β‐amyloid polypeptide (Aβ_42_) and tau protein (τ). Finally, ZnPC was integrated into the plasmalemma of living HeLa cells via vesicle‐cytomembrane coalescence, using endogenous, freely diffusing O_2_ as the coreactant to realize surface‐confined single‐cell ECL visualization.

Collectively, this work establishes a biointerface‐compatible ECL paradigm based on biolipid film‐fused ZnPC nanochannels. This platform enables multiplexed monitoring of molecular (small molecule–membrane interactions), membranous (lipid bilayer dynamic remodeling), and cellular (live‐cell membrane imaging) processes via membrane‐structural modulation. The results highlight the potential of phospholipid film‐fused ECL for ultrasensitive biointerfacial assays, and position this strategy as a robust alternative to contemporary multifactorial MSD schemes for biomembrane research.

## 2 Results

### 2.1 Characterization of ECL Nanochannel‐Nested Lipid Layers

**FIGURE 2:**
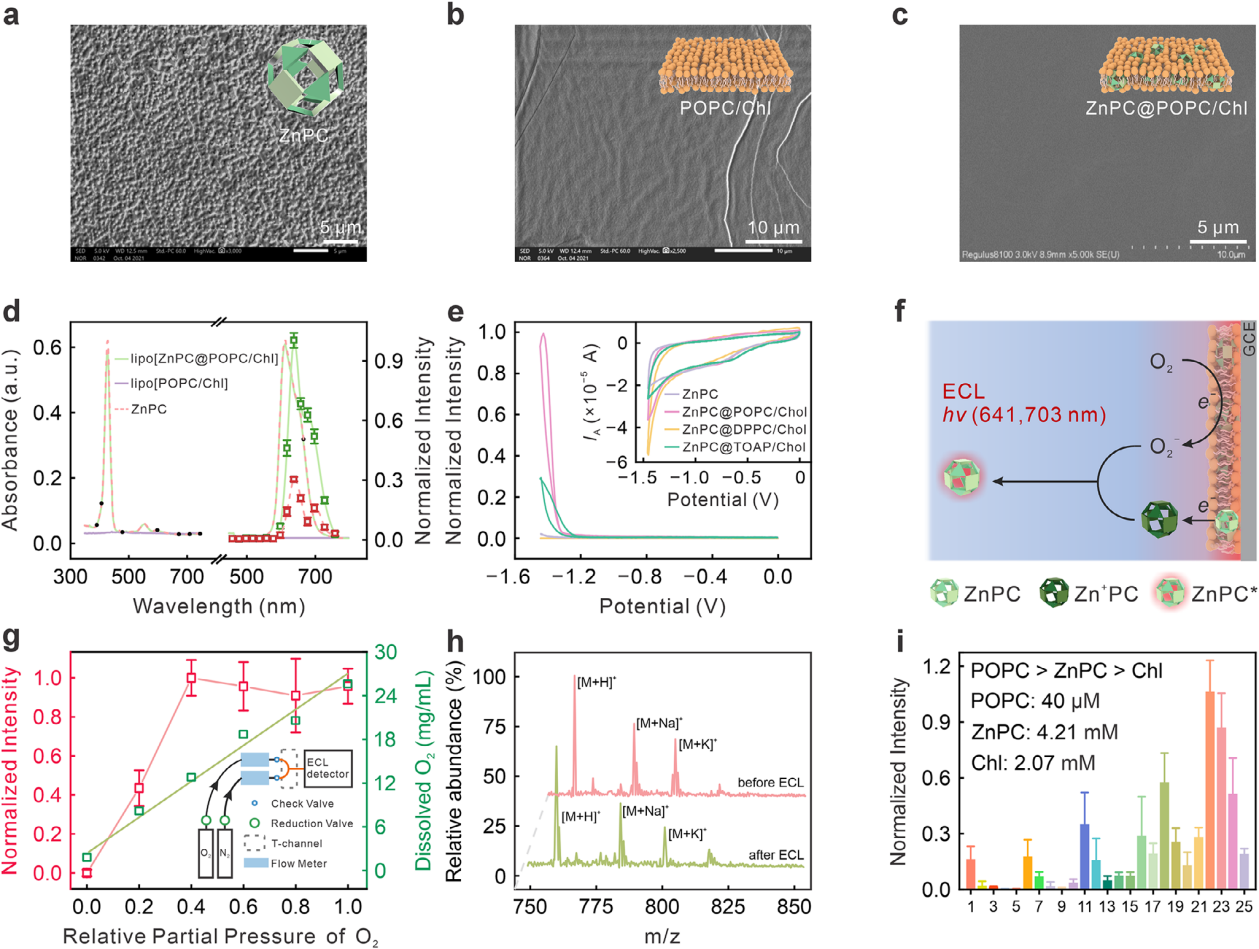
Comprehensive characterizations of ZnPC@POPC/Chl across microscale morphologies and multimodal spectroscopies. SEM microimages of (a) ZnPC, (b) POPC/Chl, and (c) ZnPC@POPC/Chl. **Inset**: corresponding structural schematics. (d) UV–vis absorbance (left *y*‐axis), and PL plus bandpass‐filtered ECL emission (right *y*) spectra of lipo[ZnPC@POPC/Chl], lipo[POPC/Chl], and ZnPCs at equal concentrations in CHCl_3_ (UV–vis and PL) and in HEPES bioassay buffer (ECL). (e) *I*_ECL_ versus potential plots of ZnPC, ZnPC@POPC/Chl, ZnPC@DPPC/Chl, and ZnPC@TOAP/Chl, each containing equivalent content of ZnPC (10 µL × 40 µm). Scan rate: 0.1 V/s; scan range: [0, −1.45] V. **Inset**: corresponding cyclic voltammograms. (f) Empirical electron‐relay roadmap about the coreactive ECL mechanism at GCE/ZnPCs. (g) ECL intensity (left *y*) and dissolved oxygen (right *y*) at varying relative partial pressure of O_2_: 0.0, 0.2, 0.4, 0.6, 0.8, 1.0. Linear relationship: *I*_D.O._ = 3.1×*c*[O_2_] − 23.2 (*R* ^2^ = 0.989). **Inset**: schematic setup of the O_2_/N_2_ mass flow control circuitry. (h) MALDI‐TOF mass spectra of POPC at GCE/ZnPC@POPC/Chl before and after ECL examinations. (i) ECL bar chart corresponding to the grouping in Table S3. The *x*‐axis numbers indicate the “Group No.”. **Inset**: component weights and optimal conditions. Data are reported as mean ± S.D. (standard deviation), *n* = 5.

ZnPCs were self‐enclosed via imine condensation, as delineated in the “Fabrication of Biofilm‐Fusable ECL Emitters” of the Supporting Information and Figure S1 [22], with C═N connectivity corroboration for three derivatives (i.e., *^n^* PC = 6×TFPP + 8×*^n^* TbTm, *n* = 4, 8, 12) in the nuclear magnetic resonance (^1^H‐NMR) spectra of Figure S4 (see Supporting Information Note, “Peak Picking in ^1^H‐NMR Spectrograms”). Figure 2a gleans the geometry of Zn^4^PC, the typical prototype (hereinafter, ZnPC defaults to Zn^4^PC unless specified as Zn^8^PC or Zn^12^PC) from scanning electron micrographs (SEM), wherein ∼150‐nm spheroidal species spread across a detachable tip of a glassy carbon electrode (GCE, Figure S5a). This deviation from the individual dimension (edge length: 2.83 nm) arises from hydrophobicity‐driven bundling among aliphatic chains, as concordantly confirmed by the contact angle (θ_c_ = 94.5°, Figure S5b). By contrast, ruptured POPC/Chl vesicles (molar ratio: 2/1) serving as a blank control confer a flat film‐forming morphology on the GCE (Figure 2b), and their hydrophilic headgroups give a θ_c_ = 13.5°. Thirdly, ZnPC (0.04 mm), POPC (4.21 mm), and Chl (2.07 mm) were reconstituted into ZnPC@POPC/Chl (protocol elaborated in “Electrode Surface Functionalization and Membrane Interaction Investigations”, Supporting Information), wherein ZnPCs dip and disperse tracelessly within the thin coating (Figure 2c), attributable to a core diagonal length (3.64 nm, discounting the stretching span of ^8^TbTm). Considering the composite ZnPC@POPC/Chl, the horizontal cross‐section is construed here as the apparent topographic thickness of the supported membrane assembly rather than a direct demonstration of a canonical hydrated SLB sheet (Figure S5c). Consistently, FRAP measurements manifested remarkable recovery with a diffusion coefficient of 1.05±0.08 µm^2^/s (Figure S6), supporting the presence of a laterally mobile membrane‐like milieu [22].

Next, the photophysical properties of ZnPC@POPC/Chl were elucidated in the merged multi‐spectral diagrams of Figure 2d. The UV–vis absorption of ZnPC@POPC/Chl liposomes (lipo[ZnPC@POPC/Chl], see “Preparation of ZnPC‐Invasive Vesicles” in Supporting Information, whose laser‐ray revealed a Tyndall effect, Figure S5d), displays a sharp Söret band and dual diminutive *Q* bands, overlapping with those of equimolar ZnPCs in chloroform, accentuating the absence of aggregation [22]. The two photoluminescence (PL) emissions, excited at (λ_ex_)_opt_ = (λ_abs_)_max_, show similar signatures, whereas lipo[POPC/Chl] delivers no discernible signals in this range. Although the PL spectra are red‐shifted by ∼20 nm relative to the ECL of GCE/ZnPC@POPC/Chl, they follow a proximal pattern, affirming that fusion within POPC/Chl does not compromise ECL efficiency; rather it safeguards ECL excitons from electrophilic extinguishing, which is advantageous for subsequent sensing applications. The ECL yield of ZnPC@POPC/Chl in O_2_‐saturated solution, referenced to ruthenium tris(bipyridyl)/tripropylamine, was 11.25% [4].

Furthermore, several similar phospholipids were compared in terms of ECL intensity (*I*_ECL_, Figure 2e), including typical natural unsaturated lipids (e.g., POPC (16:1)), natural saturated lipids (DPPC (16:0)), and a synthetic TOAP (an analogue to tetra(*n*‐octyl)ammonium, the conventional lipoid for forming ECL eutectics [26], see “Preparation of (2‐(Trioctyl‐ammonio)ethyl)octylphosphate (TOAP)” [27] above Figure S2 and ^1^H‐NMR in Figure S7). ZnPC alone cannot sustain stable ECL emanation in physiological phases, whereas ZnPC@POPC/Chl shows a superior signal at onset and peak potentials of *E*_ON_ = −1.32 V and *E*_P_ = −1.42 V, respectively. In contrast, *I*_ECL_ of ZnPC@DPPC/Chl at commensurate mole fractions fell behind, being ∼1.5% of the former, which was ascribed to the entropy‐unfavorable self‐assembly of DPPC and its weakness in withstanding hydrated polaron percolation [26].

From the charging capacitance of cyclic voltammograms (inset), the lipid depth (*d*) could be calculated via Equation S4 (Supporting Information Note: “Deducing the Depth of Phospholipid Films”): *d*_ZnPC@POPC/Chl_ = 1.07 nm, consistent with the thickness in Figure 2c, whereas *d*_ZnPC@DPPC/Chl_ = 1.41 Å, anomalously small. Additionally, the zwitterionic TOAP, tailored for RNA transfection, yielded *I*_ECL_ for ZnPC@TOAP/Chl of only one‐third that of ZnPC@POPC/Chl, with a negatively moved *E*_P_, resulting from the suboptimal permeability of the interspaced TOA organization [27]. Consequently, POPC/Chl was established as the benchmark for follow‐up functionalization.

Moving forward, the impact of coreactants on ECL was inspected in Figure S8a. *I*_ECL_ arises with the accumulation of O_2_ partial pressure (*p*_O2_), reaching a plateau at saturation, implying the improvement of ECL by dissolved O_2_ (D.O.). Conversely, purging with N_2_ inhibits *I*_ECL_ to baseline noise. Interestingly, supplementing H_2_O_2_ at a content tantamount to saturated D.O. (∼400 µm) did not increase *I*_ECL_; instead, it dropped below the air‐saturated level. This underperformance underscores the deleterious effect of H_2_O_2_ on SLB, as it oxidatively damages C═C bonds in POPC [26], thereby interrupting the integrity and leading to *I*_ECL_ decline. Consequently, O_2_ was opted as an endogenous ECL enhancer.

Figure 2g further depicts the dependence of *I*_ECL_ on *p*_O2_ by a bifurcated flowmetric setup (see Equations S1 and S2 in “Instrumentation”, Supporting Information), where *I*_ECL_ increases until a relative *p*_O2_ = 40% (≈2× atmospheric pressure) and then stabilizes, striking a balance between *c*[D.O.] (24 mg/mL, measured by an oximeter) and *p*_O2_. The coreacting concatenation between ZnPC and O_2_ was elucidated in Figure 2f, which was formulated from Equations S5–S12 and Figure S9 in the Supporting Information Note: “Explanation on ECL Cascading Coreactions”.

As aligned in Figure S8b, in aerated medium, the ECL of ZnPC@POPC/Chl stands stable with a small standard deviation of 1.51% over continuous cycles; moreover, it renders robust coefficients of variation (CVs) of 12.36% within runs and 10.35% between runs, reaffirming the reproducibility of the in situ SLB structure. Finally, the electrolytic endurance of phospholipids was verified via MALDI‐TOF. In Figure 2h, the POPC profile prior to ECL testing (*m*/*z* = 760.40, [M+H]^+^) matches the post‐experiment fragments (*m*/*z* = 760.27, [M+H]^+^), confirming the enviro‐stability of the entire system.

### 2.2 ECL‐Based Cytomembrane‐Mimicking Affinity Assays

Now that the ECL attributes of ZnPC@POPC/Chl have been anatomized, its performance was parameterized and optimized prior to upcoming applications. The influential factors include: **(i)** the formulation of filmogens, **(ii)** the selection of solutions, and **(iii)** the pH parameter. To obtain the optima of **i**., orthogonal optimizations were executed across 25 combinations of basic settings: 5 (*c*_ZnPC_ = 5, 10, 20, 40, and 60 µm) × 5 (*c*_POPC_ = 0, 0.8, 1.6, 2.4, and 3.2 mg/mL) × 5 (*c*_Chl_ = 0, 0.8, 1.6, 2.4, and 3.2 mg/mL), as tabulated in Table S3. With *I*_ECL_ adopted as the key criterion (compiled in Figure 2i), every set of five unifactorial outcomes was summed (*K*_n_) and averaged (*k*_n_ = *K*_n_ / 5, n = {1, 2, 3, 4, 5}), as listed in Table S4. According to (*k*_max_ − *k*_min_), the three components were ranked in relevance as POPC > ZnPC > Chl, producing the optimal configurations of *c*_ZnPC_ = 40 µm, *c*_POPC_ = 4.21 mM (3.2 mg/mL), and *c*_Chl_ = 2.07 mm (0.8 mg/mL). Beyond composition, HEPES (10 mm, pH 7.5) was identified as the best buffer, as manifested in Figure S10 **(ii**, **iii**), together with representative ECL images (see “Wide‐Field Full‐Electrode ECL Visualization” in Supporting Information).

**FIGURE 3:**
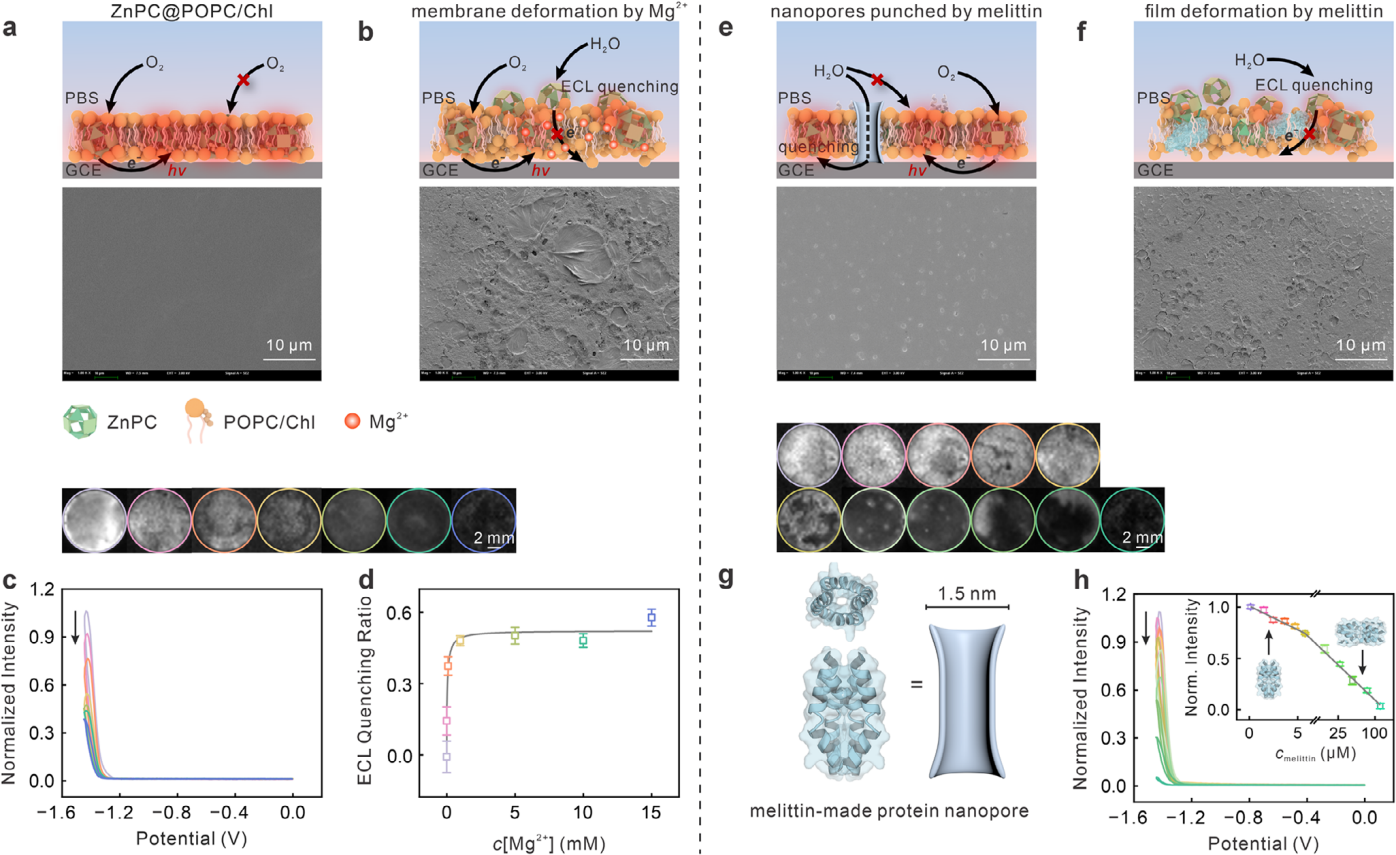
ECL‐based cytomembrane‐mimetic measurements. **Left panel**: SEM micrographs of ZnPC@POPC/Chl (a) before and (b) after exposure to 10 mm MgCl_2_; above are schematic side views of the surface architecture. (c) *I*_ECL_ versus potential profiles as a function of *c*[Mg^2+^] ranging from 0.01 to 15 mm; above is an array of ECL images acquired at corresponding concentrations, indicated by the circle color. ECL images were obtained under cyclic voltametric excitation (see “Wide‐Field Full‐Electrode ECL Visualization” in Supporting Information. (d) Langmuir isothermal fitting between normalized Δ*I*_ECL_ (i.e., 1−*I*_ECL_/*I*_ECL_^Θ^) and *c*[Mg^2+^]. **Right panel**: SEM micrographs of ZnPC@POPC/Chl after association with (e) 5 µm and (f) 100 µm melittin; above are schematic side views of melittin‐membrane complexation. (g) Structure of melittin molecule and its membrane‐perforating polymers. (h) *I*_ECL_ versus potential plots at *c*_melittin_ changing from 1 to 100 µm. **Inset**: corresponding calibration curves fitted over segmented scopes of [1, 5] and [5, 100] µm. Above is an album of ECL exposures at corresponding concentrations, indicated by the circle color. Data are expressed as mean ± SD, *n* = 5.

From the fractional ratio of *c*_ZnPC_/*c*_POPC_ ≈ 1/100, ZnPC is expected to scatter sparsely within the POPC/Chl SLB, thereby maintaining membrane functionality. Accordingly, a preliminary probe was conducted to test whether ion channeling could occur under these conditions and elicit ECL feedback. Herein, two divalent cations (Mg^2+^, Ca^2+^) were chosen to enact electromigration across the membrane at negative potentials. Prior to their introduction, the SEM of ZnPC@POPC/Chl shows a smoothly spread scenery (Figure 3a). In stark contrast, adding 10 mm MgCl_2_ made the SLB sheet to scrunch (Figure 3b), attributable to its electro‐repulsion with phosphocholine, therefore neutralizing electrostatic equilibrium within the SLB [28].

To instantiate this interpretation, ECL titration was performed over *c*[Mg^2+^] ranging from 0.01 to 15 mm. As unfolded in Figure 3c, *I*_ECL_ declines during the densification of Mg^2+^; the relative attenuation amplitude, defined as (1 − *I*_ECL_ / *I*_ECL_^Θ^), fits a binominal Langmuir isotherm (Figure 3d), with parameters presented in Table S5. This analysis discloses dual ECL kinetics: *K*_D1_ and *B*_max,1_ betoken the binding power and probability on POPC/Chl, respectively; while *K*_D2_ and *B*_max,2_ correspond to ZnPC [22]. The *K*_D_ values align closely with those obtained from molecular simulations and isothermal titration calorimetry [29, 30]. From *K*_D1_ >> *K*_D2_ and *B*_max,1_ < *B*_max,2_, it is clear that ionic infiltration predominates over on‐SLB sorption, such that transmembrane transport Mg is accompanied by water as well as other ^1^O_2_ scavengers, whose seepage ultimately culminates in *I*_ECL_ depletion. This mechanistic rationale is further verified by fluorometric and impedimetric controls (Figure S11). For Ca^2+^, its ECL responses resemble that of Mg^2+^ (Figure S12), and both surging segments allowed linear calibration. At an analyte concentration of 10 mm, the intra‐assay CVs for five independent determinations of Mg^2+^ and Ca^2+^ were 5.05% and 6.95%, respectively, while the inter‐assay CVs were 5.81% and 2.41%, thereby verifying repeatability.

Proceeding one step further, the SLB‐binding melittin was picked to probe membrane–macromolecule interactions. As a cell‐penetrating peptide, it comprises 26 amino‐acid residues [16], as depicted in the ribbon representation of Figure 3g. At lower concentrations (0–5 µm), melittin dimerizes or tetramerizes into ∼300‐nm membrane‐scale defects through peptide accumulation in ZnPC@POPC/Chl (Figure 3e; Figure S13a). This process brings about bilayer breach and abates *I*_ECL_ owing to the ingress of ECL inhibitors (e.g., H^+^). Albeit this stage is reversible, *I*_ECL_ falls as a function of *c*_melittin_: *I*_ECL_/*I*_ECL_^Θ^ = 362.4·*c*_melittin_ − 10808.2 (*R* ^2^ = 0.975), with a steeper slope (Figure 3h; upper section of Table S6), suggesting significant SLB sensitivity to structural sabotage; the corresponding *I*_ECL_‐potential traces is exhibited in Figure S13b. Further elevating *c*_melittin_ to 100 µm imposes irreversible injury on lipid layers, evidenced by widespread wrinkling (Figure 3f; lower section of Table S6), despite a gentler slope described by *I*_ECL_/*I*_ECL_^Θ^ = 88.0·*c*_melittin_−9293.5 (*R* ^2^ = 0.996).

To differentiate the distinct dynamics of signal attenuation between ionic channeling and peptide poration, we further scrutinized the spatial spread of ECL imagery using the coefficient of variation. As summarized succinctly in Table S7, the Mg^2+^‐treated ZnPC@POPC/Chl displayed diminished *I*_ECL_ with a consistently constrained CV (coefficient of variation, 0.15–0.20), derivable from uniformly distributed ion binding. In stark contrast, melittin exposure provoked pronounced CV (up to 0.72), portraying punctate formation of localized cross‐membrane pores and heterogeneously hosted defects on the electrode surface (Table S8). Consequently, despite the overall trend of signal suppression, the distinct CV–intensity inclinations indicate that CV may serve as a viable variable for discriminating distinct interfacial ligands and their mediated membrane perturbation modes (Figure S14). The within‐work (ten‐time tests at certain *c*_melittin_) and between‐batch (given *c*_melittin_ across eight electrodes) precisions across two intervals were attested to be 2.4% (5 µm) and 1.7% (50 µm), and 12.5% (5 µm) and 10.9% (50 µm), respectively. Altogether, the piecewise bilinear fits faithfully feature the phase transition of the melittin‐membrane complex, which has identical implication with conventional biolayer interferometry [31], thus validating the validity of ECL‐luminogenic lipids in illuminating lipo‐peptidyl interplay.

**FIGURE 4:**
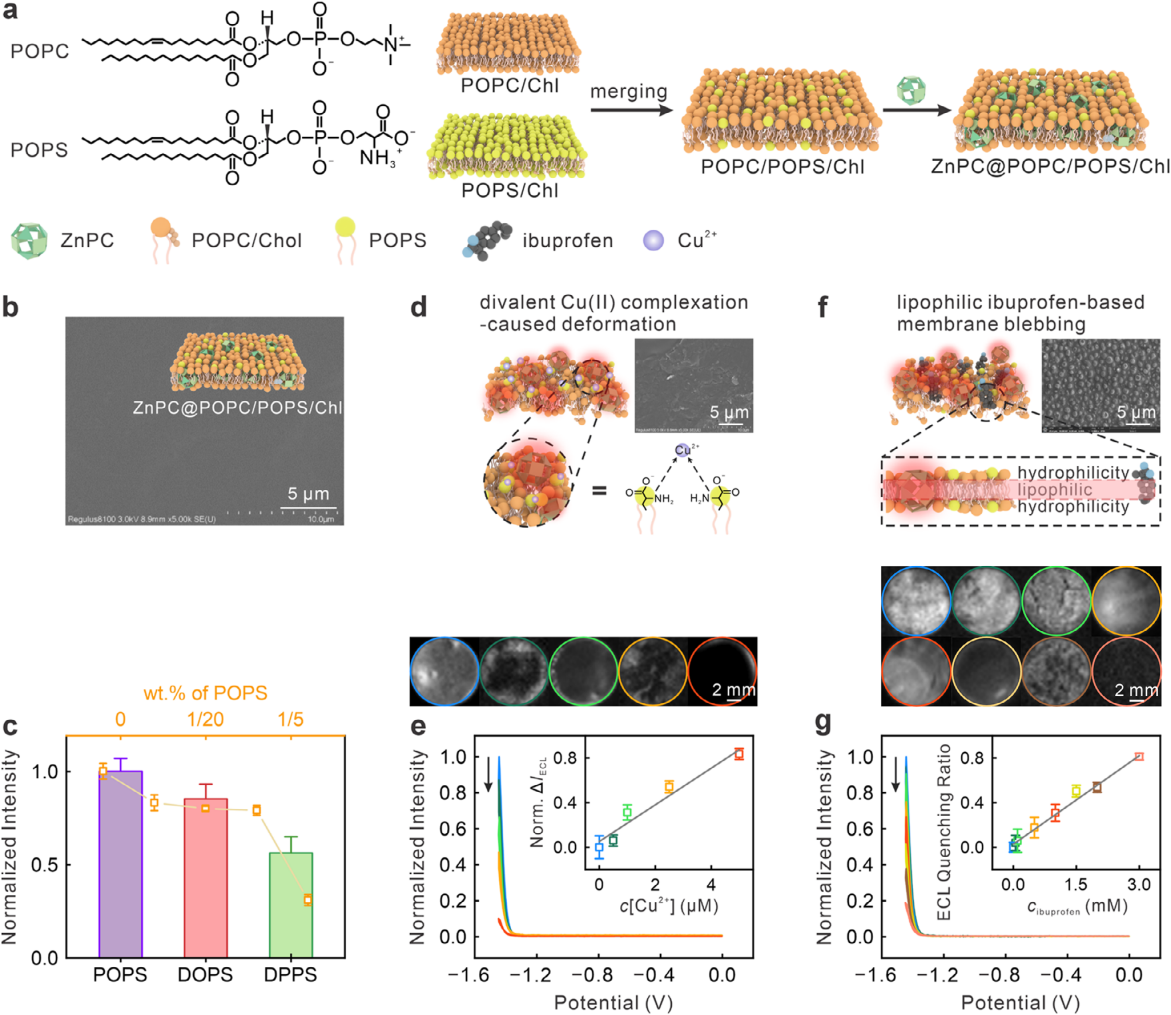
Preparation of POPS‐composited SLB for site‐selective sensing. (a) Flowchart about building a binary phospholipid bilayer, that comprises POPC and POPS into ZnPC@POPC/POPS/Chl. (b) SEM snapshot of ZnPC@POPC/POPS/Chl. **Inset**: 3D depictions of the di‐lipidated SLB. (c) Phospho‐L‐serine panel (POPS, DOPS, and DPPS) screening (histogram), together with the POPS proportion‐dependent *I*_ECL_ responses (lines and dots). (d) SEM specimen of ZnPC@POPC/POPS/Chl+Cu^2+^ (5.0 µm). **Inset**: mechanistic modeling of the POPS‐binding behavior. (e) *I*_ECL_‐versus‐potential plots of *c*[Cu^2+^] at 0.0, 0.5, 1.0, 2.5, 5.0 µm. **Inset**: corresponding calibration correlation between the normalized Δ*I*_ECL_, i.e., (*I*_ECL_^Θ^ − *I*_ECL_)/*I*_ECL_^Θ^ (quenching percentage), and *c*[Cu^2+^]; above is an array of ECL images acquired at corresponding concentrations, indicated by the circle color. ECL images were obtained under cyclic voltametric excitation (see “Wide‐Field Full‐Electrode ECL Visualization” in Supporting Information. (f) SEM sample of ZnPC@POPC/POPS/Chl+ibuprofen (3.0 mm). **Inset**: mechanistic manifestation of SLB‐binding behavior. (g) *I*_ECL_ *vs*. potential profiles of *c*_ibuprofen_ from 0.05 to 3 mm. **Inset**: calibration curve between the normalized Δ*I*_ECL_ and *c*_ibuprofen_. Above is an album of ECL exposures at corresponding concentrations, indicated by the circle color. Data are presented as mean±SD, *n* = 5.

In the next move, to meet the realistic regime, another common constituent, POPS, was employed to compose ZnPC@POPC/POPS/Chl (Figure 4a), which not only adjusted architectural attributes akin to a native context, but afforded accessible amine moieties in phosphor‐L‐serine as specific sites for affinity [26]. As clearly captured in Figure 4b, the POPS‐inserted SLB presents a polished surface, similar to that of Figure 3a, with an optimum occupancy of POPS/POPC = 1/10 (mol.%) in Figure 4c, i.e., *c*_ZnPC_:*c*_POPC_:*c*_POPS_:*c*_Chl_ = 1:100:10:50. Consequently, comprising certain POPS curtails *I*_ECL_ only marginally (Figure S15). On the contrary, the participatory presence of POPS strengthens surface electronegativity, namely the lipid‐layer stability (Figure S16). Two supplementary substitutes, DPPS and DOPS, were handpicked to replace POPS but surpassed strikingly in *I*_ECL_, which owes to: **(i)** the considerable crystalline‐to‐gel transition temperature (41°C–45°C) of saturated DPPS, constraining charge transfer and O_2_ accessibility [26]; and **(ii)** the double‐bonded C═C in DOPS, where strong steric hindrance among tail groups gets ZnPC in a disordered and non‐defensive domain. Thus, POPS, by its balanced backbone rigidity and rheological resilience, becomes the best‐behaved building block; moreover, the POPS proportion parallels physiological prevalence in mammalian cells and erythrocytes [32]. Thereafter, the requisite conditions were re‐examined to be pH 8.0 phosphate‐buffered saline (PBS, Figure S17). Under this specified setting, repetitive ECL retained a relative standard deviation (RSD = 0.71% in Figure S18), signifying superior steadiness.

Afterward, ZnPC@POPC/POPS/Chl was adapted accordingly for Cu^2^^+^ ligation, which possesses the strongest stepwise stability constant in the Irving–Williams series [33]. Concomitantly, copper cations exert exceptional effects on cytomembranes [34], which were expected explicitly to be explored in this ensemble. Figure 4d depicts a detailed local landscape after the addition of Cu^2+^, wherein wrinkles and ridges are readily revealed. This behavior is derived directly from the capability of Cu^2+^ to couple with ─NH_2_ groups on the negatively charged hydrophilic heads of POPS, ultimately culminating in lipid peroxidation and membrane disruption [34]. As *c*[Cu^2+^] climbs continuously, rupture of the SLB allows water, acting as a radical scavenger, to permeate inward, thereby reducing *I*_ECL_ (Figure 4e). The overall inclination conforms to the Langmuir isotherm, with linearity at lower thresholds (0–5 µm, inset, Table S9). In parallel processes, a Cu^2^^+^‐responsive probe (Rhodamine B hydrazide) was applied to solutions preceding and posting ECL practices to justify the above arguments (Figure S19), both befitting the outcomes from microfluidic assays [35].

Beyond ion interrogation, sensing small medicinal molecules is likewise necessary and notable for preclinical pharmacological evaluation. To be specified, ibuprofen, an anti‐inflammatory over‐the‐counter drug, has hydrophobicity that helps passive diffusion across the cytomembrane to inhibit intracellular cyclooxygenase [36]. To catch cross‐membranous mechanisms, ZnPC@POPC/POPS/Chl was interrogated with ibuprofen. As attained apparently in Figure 4f, numerous blebs were brought into being on the as‐synthesized SLB. This deformation was derived dominantly from the film‐filling ibuprofen molecules, whose charged carboxylate tails repel phospholipid headgroups, arousing a surge in surface tension and in consequence changing curvature locally [37]. Figure 4f inset illustrates this underlying principle. With *c*_ibuprofen_ accumulating gradually, ∆*I*_ECL_ linearizes steadily up to 3 mm (Figure 4g inset), covering its physiological presence in plasma and coinciding consistently with in vitro evaluations from stalagmometer and second‐harmonic/sum‐frequency spectrometry [36, 37], hence highlighting the harmony of ECL with SLB‐based surface‐affinity assays.

Furthermore, the spatial spread of ECL emissions was estimated to disentangle interaction mechanisms of Cu^2+^ and ibuprofen. As documented in Table S10, the addition of Cu^2^^+^ triggered a sharply soar in CV (reaching up to 0.53), indicative of severe spatial heterogeneity. This phenomenon is attributed accurately to high‐affinity coordination between Cu^2+^ and serine headgroups of POPS. In contrast, ibuprofen treatment induced incrementally a moderate rise in CV from 0.19 to 0.24 (Table S11), aligning with a structural strain model wherein amphiphilic ibuprofen insertion causes widespread wrinkling and distributed surface roughness, rather than formation of localized lipid microdomains or Cu^2+^ clusters as further exemplified by the CV–intensity curves in Figure S20.

### 2.3 ECL‐Embedded SLB Substrate for Sensing Alzheimer's Markers

**FIGURE 5:**
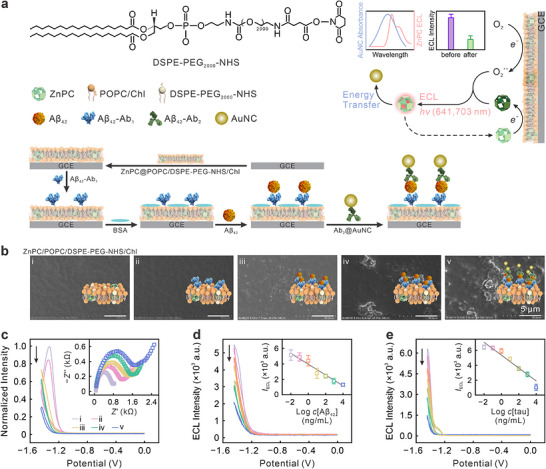
Preparation of DSPE‐PEG‐NHS‐hybridized SLB substrates for site‐specific sensing on Alzheimer's markers. (a) Schematic sketch about the layer‐by‐layer construction of a ZnPC/POPC/DSPE‐PEG‐NHS/Chl‐underlain universal substrate for immuno‐ and aptameric assays, by use of the so‐called “ECL‐RET” transducer from ZnPC the source to AuNC the labeled drain; inset with the chemical formula of DSPE‐PEG_2000_‐NHS. (b) Sequential SEM microphotographs substantiating the SOP (from left to right): GCE/ZnPC/POPC/DSPE ‐PEG‐NHS/Chl (i), i + Aβ_42_‐Ab_1_ (ii), ii + BSA (iii), iii + Aβ_42_ (iv), and iv + Ab_2_@AuNC (v).(c) ECL feedbacks to the fabrication flow in line with b (*c*[Aβ_42_] = 1 ng/mL). **Inset**: Nyquist plots pertaining to the same stepwise setting in 5 mM Fe(CN)_6_^3−/4−^ at an open‐circuit potential of 0 V and frequencies from 10^−2^ to 10^5^ Hz. *I*_ECL_ vs. potential plots recorded in aliquots of 0.01‐to‐10^4^ ng/mL for d) Aβ_42_ and e) tau proteins in PBS. **Inset** shows the corresponding calibration curves. Data are presented as mean ± S.D., *n* = 5.

The ECL exploitation of phospholipids was primarily accomplished through liposomal labeling or self‐assembled monolayer [38], it is rare for a multicomponent lipomembrane to serve straightly as an ECL sensor substrate. Here, we leveraged the abovementioned advantages of SLB to detect a dual set of biomarkers: Aβ_42_ and tau, in blood [39], which hold the hinge for early Alzheimer's disease diagnosis. To this purpose, DSPE‐PEG‐NHS was decided as a membrane receptor to receive recognizers, while reducing nonspecific sorption. In this context, the proof‐of‐concept walkthrough of the ZnPC@POPC/DSPE‐PEG‐NHS/Chl construct is dissected in Figure 5a and discussed in “ECL Biosensor Build Based on Lipid Layers for Alzheimer's Marker Measurements” (Supporting Information). In a stepwise scheme: **(1)** DSPE‐PEG‐NHS was doped into ZnPC/POPC/Chl to implant points for antibody or aptamer attachment; **(2)** Either primary antibody (Ab_1_) or aptamer was anchored onto the interface via amidation; **(3)** BSA (bovine serum albumin) was applied to block nonspecific sites; **(4)** Targets were pulled down through antigen‐antibody (for Aβ_42_) and τ‐aptameric (Table S2) affinities; and **(5)** Secondary antibody (Ab_2_) tagged with gold nanocluster (AuNC) was mounted atop to quench ECL via resonance energy transfer (ECL‐RET) [40], which underpins a simplified signaling strategy.

Getting down to the standardized operating procedures (SOPs), the dopant density of DSPE‐PEG_2000_‐NHS was weighed and warranted to be 5 wt.‰ (1.27 mol.‰) of POPC (Figure S21), under which condition *I*_ECL_ still stays above 90% of the original and remains robust as always (Figure S22). Furthermore, methodical modulations were made upon *c*[Aβ_42_‐Ab_1_], *c*[τ‐apt], and their incubation times, as assorted in Figure S23, and the appropriate preconditions are *c*[Aβ_42_‐Ab_1_] = 0.7 µg/mL and *c*[τ‐apt] = 1.0 µg/mL with both *t*_incubation_ = 70 min. Whereafter, we sought SEM for spotting the success of the stepwise sandwiched assembly. As arranged in a row of Figure 5b, ZnPC@POPC/DSPE‐PEG‐NHS/Chl, the matrical membrane manifests an even exterior comparable to previous compositions **(i**). Though Aβ_42_‐Ab_1_ or τ‐apt immobilization on DSPE‐PEG‐NHS barely altered this appearance **(ii**), the unspecific physisorbed occupancy by BSA began and beget glomeration **(iii**). Antigen (Ag, 1 ng/mL) attachment augmented the asperity by haptenic complexation **(iv**). At last, the docked Ab_2_@AuNC maximized the morphological nonuniformity **(v)**. Such a protocol progressively pares *I*_ECL_ down, as amassed in Figure 5c (i.e., 10 000>9740>9510>4920>1130 a.u.), while escalating the electrochemical resistance in the inset (947<1211< 1407<1624<2273 Ω). These monotonic and matched *I*_ECL_ and *R*_ET_ changes conclusively verify the bi‐epitope pinching of Aβ_42_‐Ab_1_ (τ‐apt) / Ag / Ab_2_@AuNC, facilitating an ECL‐RET effect between ZnPC as the donor and AuNC as the acceptor (Figure S24a), thus significantly sensitized the signal by 4.4‐fold (4920/1130).

Succedently, titrations were conducted using Aβ_42_ and tau as titrants, as tracked in Figure 4d,e, respectively. Their correlated calibration curves were quantitatively qualified in Table S12, that *I*_ECL_ dive discernibly with logarithmic *c*[Aβ_42_] and *c*[tau] over the range of 0.01–10^4^ ng/mL. The limits of detections (LOD) were determined to be 2.25 pg/mL for Aβ_42_ and 3.24 pg/mL for tau, calculated using the conventional criterion *I*_LOD_ = *I*_blank_ − 3σ_blank_, where *I*_blank_ and σ_blank_ were obtained from blank measurements (*n* = 5), and was back‐calculated from the fitted calibration curve. These performances are comparable with those of ELISA, the mainstream methodology (Figure S24b), and competitive among archived approaches (Table S13). Residual regression revealed the robustness of the linear model across the reported dynamic range, with no evident systematic shift (Figure S25).

To ascertain the actual applicability, the concentrations of Aβ_42_ and tau in sera samples from Alzheimer's patients were gathered and given to tentative tryouts via this ECL‐RET readout and the microplate‐reading ELISA, with their outcomes proving commeasurable and consistent in Table S14. These results, together with satisfactory specificity (Figure S26) and impressive intra‐batch integrity (RSD of 7 replicates: Aβ_42_ 1.27%, tau 0.98%) as well as inter‐batch invariance (RSD of 7‐day repeats: Aβ_42_ 8.87%, tau 7.46%) at a median *c*_antigen_ = 1 ng/mL, represented the remarkable repeatability of this SLB‐based ECL‐RET route, together with its superb sensitivity and surface‐confined interfacial readout, rendering it ready for real cyto‐membranal fragment‐fusing assays.

### 2.4 Mapping Monocellular Adhesion via Membrane‐Buried ECL

ECL monocellular microscopy may materialize as a transformative tool for realtime membrane‐melding mapping and adhesion profiling. For this purpose, we put together an ECL visualizer (Figure S3), incorporating a confocal cell‐culture cartridge and a potentiostat‐tuned tri‐electrode ensemble (see “Cell Culture and Single‐Cell ECL Imaging Procedures” in Supporting Information for further information). On the other hand, ZnPC‐fused fusogenic vesicles were maneuvered through molecular engineering: the nanoemitter entered and dwelled within POPC/Chl during thin‐film hydration, ensued and executed by cryo‐thaw cycling and consecutive controlled extrusion to yield mono‐disperse liposomes.

**FIGURE 6:**
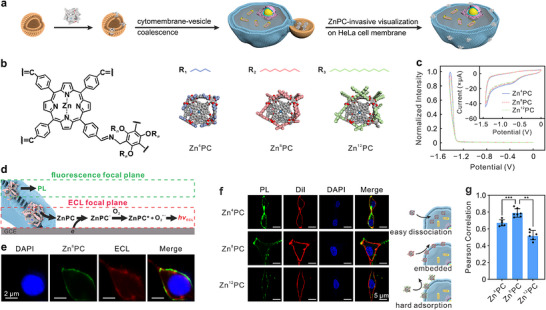
Formation of fusomes between ZnPC‐invasive vesicles and HeLa cell as a model for ECL‐based cytomembrane imaging. (a) Flow‐chart of the liposome‐cytomembrane melding: (1) Coextrusion of ZnPCs and POPC/Chl to form ZnPC‐channeled liposomes; (2) Graphical guidance and glimpse of vesicle‐cell coalescence; (3) Melding modalities and manifestations: time‐resolved membranous interface kinetics after the fusion of ZnPC. (b) General roadmap for fabricating Zn^4/8/12^PC, the homologous membrane‐interactive scaffold bearing respective ^4/8/12^TbTm ligands, by recourse to dynamic C═N networking and nucleation. (c) *I*_ECL_‐versus‐potential plots of Zn^4^PC (i), Zn^8^PC (ii), and Zn^12^PC (iii) @POPC/Chl at GCE in PBS. **Inset**: the corresponding cyclic voltammograms (scan rate: 100 mV/s). (d) Diagrammatic description about the cellular film‐fused ECL visualizer and its surface‐confined focal plane relative to that of epifluorescence microscopy. (e) Brightfield, darkfield (PL, green), ECL (red), and merged PL‐ECL microscopic images of HeLa cells, harboring the nanochannel Zn^8^PC. Pearson correlation: 0.643. Cells were inoculated onto ITO electrodes at the bottom of a confocal cuvette and proliferated to 10^5^ cell/mL. ECL exposures were elicited in O_2_‐saturated solution (PBS, pH 7.4), stimulated by cyclic voltammetry (scan sweeping between 0 and −2 V over a 120 s capture, Figure S32a). Objectives: Plan, Fluor ∞/−, N.A. 0.17, W.D. 2.1 (20X) and 0.66 (40X). (f) Conceptual cartoon about how altering the length of ZnPC side‐chains conduces to divergent distributions and fates around the cytomembrane: near the inner leaflet until endocytosis (Zn^4^PC), cross‐membrane (Zn^8^PC), and extracellularly at outer leaflet (Zn^12^PC). (Nikon, Japan; Filter cube: FITC, incident/emergent: 488/525 nm; Objective: 63X, Plan Apo, W.D. ∞/0.17, N.A. 1.40, oil immersion; Detector: ORCA Flash 4.0 sCMOS camera, 2048×2048 pixel^2^, Hamamatsu Photonics) (g) Pearson correlation profiling in terms of the fluorescence signals of ZnPC and Dil (1,1'‐dioctadecyl‐3,3,3',3'‐tetramethylindocarbocyanine perchlorate). Shown are mean ± S.D. from ten individual cells (HeLa‐Zn^4^PC vs. HeLa‐Zn^8^PC, HeLa‐Zn^8^PC vs. HeLa‐Zn^12^PC). **P < 0.01, ***P < 0.001, two‐tailed Student's *t*‐test.

One unexpected edge and extra advantage is that lipo[ZnPC@POPC/Chl] could invade and integrate into HeLa cells, and coalesce and coexist without additional assistance from folate ligation, e.g., DSPE‐PEG‐FA (folic acid). Different from traditional tags tied to cytomembranes via covalent conjugation, the present strategy utilizes lipids, the inherent ingredients of vesicles, for self‐adaptive staining. Specifically, ZnPCs were effectively encapsulated within lipid bilayer via vesicle‐membrane merging (Figure 6a), by virtue of the film fluidity and familiarity to cytomembranes, which simultaneously secures a microenvironment featuring both reactant permeability and redox compatibility [22].

Of note, the carbon chain length at vertices influences the film‐filling efficacy of ZnPC. To resolve this relation‐ship, extra two ^8/12^TbTm ligands were customized for the Zn^8/12^PC encagement (Figure 6b), as ascertained in the foregoing Figure S4. Figure S27 further found that altering the alkyl chain configurations leaves and lends mostly commensurate features in ECL, PL and UV–vis spectral profiles of lipo[Zn^4/8/12^PC@POPC/Chl] (*c*_ZnPC_:*c*_POPC_:*c*_Chl_ = 1:100:50); likewise for the overlapped *I*_ECL_ and cyclic voltammetric curves at GCE/Zn^4/8/12^PC@POPC/Chl (Figure 6c). both jointly justify that prolonging porphyrinic periphery perturbs the photophysical properties trivially.

To test these three variants on cells, vesicle concentration and incubation time was prioritized to optimize via visualization in confocal (refer to “Confocal Visualization of ZnPC Vesicle Internalization” in Supporting Information). Prior to the transfection experiment, cell membrane integrity and adhesion rate were verified using DiSC3(5) (3,3'‐dipropylthiadicarbocyanine iodide) and Calcein‐AM (calcein acetoxymethyl ester), respectively (Figure S28). By pixelated photocounting in pictures of Figures S29 and S30, *I*_PL_ from cell‐correlated Zn^4/8/12^PCs were picked up and processed as the metric (see “Pearson's Correlation Coefficient Computation” in the Supporting Information Note), and 5 µM Zn^8^PC with 60‐min cultivation was identified as the proper operational parameters.

Thereafter, ECL imaging on cytomembranes was underwent with HeLa cells adhered onto an indium‐tin‐oxide (ITO) slide. As exemplified in Figure 6e, both ECL and PL patterns were predominantly captured and confined on cell membranes with conspicuous spatial overlaps (see “Confocal Colocalization for Cytomembranal Visualization” in Supporting Information). In spite of this, the PL distribution of ZnPC prefers positioning on the outer cellular surface, conforming to the reference report of a ∼1.2‐µm axial displacement between ECL and PL focal planes, as schematized in Figure 6d [41], i.e., the ECL‐emitting plane is placed closer to the electrode due to the surface‐confined effect [1, 2].

Of note, under our live‐cell visualization conditions, cathodic polarization does not necessarily imply sustained or spatially homogenous hydrogen evolution reaction (HER) over the entire electrode, because the practical HER onset on ITO is dictated by its limited cathodic activity and spatially heterogeneous interfacial response rather than by thermodynamics alone [42]. Over membrane‐contacting regions, the phospholipids further functions as a hydrophobic and highly resistive barrier that inhibits interfacial ion transport and electron transfer, thereby disfavoring HER (or OER) beneath intact membrane‐coverings [43]. At bare or defect‐dwelled ITO, cathodic polarization may motivate local alkalization, and accordingly the pH increase induces the negative move of H^+^/H_2_ equilibrium potential, thus imposing a self‐limiting effect on further H^+^ reduction [44]. On the other hand, under buffered, high‐ionic‐strength scenarios, ion‐screening confines the electric field and primary potential drop to the electrical double layer, so electrode‐driven disturbances are localized on the immediate interface of electrode/ electrolyte rather than propagating profoundly into the bulk medium [45]. Any ROS generated under these regimes would likewise keep spatially localized and short‐lived in this film‐forming configuration, not uniformly diffusing into the bulk. Taken together, these electrochemical expounding pinpoints that the perturbation under the present imaging conditions is transient and interfacially confined, consistent with the observed cell‐viability results.

Aside from that, although the absence of membrane permeation‐promotor prevented ZnPCs from encroaching the cytoplasm, cutting the carbon chains (Figure 6f) or extending the incubation periods might cause unintended internalization of ZnPC [46]. The latter would overstretch the on‐electrode cellular protrusions into a “shallow” substrate, which could not restrict ZnPCs tightly within membranes; in other words, the thickness of a cytomembrane must exceed the lateral length of ZnPC [26]. Confocal colocalization with the early endocytosis marker Rab5 conferred a consistent phenomenon (Figure S31).

Previous post‐insertion investigations upon synthetic porin‐mimetic models hinted their targeting toward hydrophobic protein‐sitting transmembrane domains [22, 23]. This is because cellular pseudopodia adhering to heterosurfaces would stir suboptimal thickness, thereof fettering and frustrating the function of transmembrane proteins. In this way, this method could illuminate the on‐membrane metabolically active perimeter, spatially pertinent to the protein‐crowding channel areas, whereby warranting the signal specificity.

Contradictory to current cyto‐imaging methodologies by the ECL technique, this facile flow afforded fusomes of luminophores in the cytomembrane, conquering or circumventing common challenges at: **(1)** Conventional chemical coupling is cytotoxic and compromising in most cases, primarily contributed by covalent crosslinkers (e.g., EDC/NHS) that impacted film fluidity, subsequently activating apoptotic signaling pathways [8]; **(2)** This membrane‐bound blueprint streamlined SOPs by canceling cycles for biolabeling (e.g., iterative activation / deprotection plus purification) [8], hence avoiding operator‐prone errors and sample loss in procedural processing; **(3)** The cytotoxicity of organo‐amines (e.g., TPrA), the anodic coreactant, is accredited to their redox‐active alkylamine moieties, which induce oxidative deterioration to cellular components via radical regeneration during ECL stimulation (impairing membrane integrity and mitochondrial utility) [8], thus being less compatible with live‐cell visualizations than our solution here.

Finally, this membrane‐melding scheme succeeded in ECL cytometry over a wide window (see “Cell Counting” in Supporting Information). By recording fusome formation between ECL‐active vesicles and HeLa cells as well as their adhesion at the electrode (Figure S32b), the ECL signals could be sensed across 10^5^–10^10^ cells (Figure S32c). Importantly, cytotoxicity assays (see “Evaluation of Cellular Cytotoxicity” in Supporting Information) authenticated that ZnPCs, the fusogenic agent at 5 µm, maintains only mild cytotoxicity at most (Figure S32d).

## 3 Conclusion

In conclusion, an ECL‐emissive, membrane‐interactive scaffold fabricated and fused into ZnPC@POPC/Chl highlighted high‐fidelity emission and versatile functionality. Systematic characterization demonstrated its adaptability for incorporating native and artificial lipids into membrane mimetic models for in vitro exploration of divalent cation‐channelling, cross‐membrane melittin boring, liposoluble ibuprofen binding, and lipo‐oxidative Cu(II) complexation. These processes were further fathomed and formulated with surface state‐sensitive ECL, yielding thermodynamic and kinetic indices consistent with conventional benchmarks. Moreover, an ECL‐RET immunosensing strategy succeeded precise, reproducible quantification of Aβ_42_ and τ proteins, overtopping previous methods in LODs. Zn^8^PC‐snorkeling liposomes illuminated individual HeLa cell basements brightly and briskly through membrane merging, enabling an endocoreactant‐energized ECL visualizer with excellent cell viability and enhanced interfacial insight beyond epifluorescence imaging. Collectively, these serial solutions underpinned the pluripotent capacity of phospholipid film‐fused ECL for ultrasensitive biointerfacial assays, positioning it as a robust competitor to contemporary multifactorial MSD schemes.

## Conflicts of Interest

The authors declare no conflicts of interest.

## Supporting information




**Supporting File**: advs75163‐sup‐0001‐SuppMat.docx.

## Data Availability

The data that support the findings of this study are available from the corresponding author upon reasonable request.
