# Supplementary material for: Biolipid Film‐Fused Electrochemiluminescence for Multipurpose In Situ Bioassays
Source: Adv Sci (Weinh). 2026 Apr 7;13(36):e24242. doi: 10.1002/advs.202524242 (PMC13317783; doi:10.1002/advs.202524242)
Supplement: Supplementary file 1 — Supporting File: advs75163‐sup‐0001‐SuppMat.docx. [file ADVS-13-e24242-s001.docx]

SUPPORTING INFORMATION

**Biolipid Film-Fused Electrochemiluminescence for Multipurpose *in situ* Interfacial Assays**

Jialiang Chen,^‡a^ Bin Li,^‡a^ Yingying Wang,^‡b^ Fulin Zhu,^b^ Wenxuan Yang,^a^ Xuanyu Fu,^a^ Tiantian Man,^b^ Qiuyue Wu,*^a^ Kewei Ren,*^c^ and Shengyuan Deng*^a^

**^a^** *School of Environmental and Biological Engineering*, *Nanjing University of Science and Technology*, *Nanjing 210094*, *China*;

**^b^** *School of Mechanical Engineering*, *Nanjing University of Science and Technology*, *Nanjing 210094*, *China*;

**^c^** *School of Chemistry and Chemical Engineering*, *Nanjing University of Science and Technology*, *Nanjing 210094*, *China*.

* Corresponding authors: qiuyue0815@njust.edu.cn, kwren@njust.edu.cn, sydeng@njust.edu.cn

^‡^ These three authors contributed equally to this work.

**TABLE OF CONTENTS**

[1. Supplementary Experimental Details S−5](#_Toc219711965)

[Chemicals and Materials S−5](#_Toc219711966)

[Table S1 S−5](#_Toc219711967)

[Table S2 S−6](#_Toc219711968)

[Instrumentation S−6](#_Toc219711969)

[Fabrication of Biofilm-Fusable ECL Emitters (ZnPC) S−7](#_Toc219711970)

[Figure S1 S−8](#_Toc219711971)

[Preparation of Small Unilamellar Vesicles and Supported Lipid Bilayers S−8](#_Toc219711972)

[Preparation of (2-(Trioctylammonio)ethyl)octylphosphate (TOAP) S−9](#_Toc219711973)

[Figure S2 S−9](#_Toc219711974)

[Preparation of ZnPC-Invasive Vesicles S−9](#_Toc219711975)

[Fluorescence Recovery After Photobleaching (FRAP) S−10](#_Toc219711975)

[Electrode Surface Functionalization and Membrane Interaction Investigations S−10](#_Toc219711976)

[Wide-Field Full-Electrode ECL Visualization S−11](#_Toc219711977)

[ECL Biosensor Build Based on Lipid Layers for Alzheimer’s Marker Measurements S−12](#_Toc219711978)

[Cell Culture and Single-Cell ECL Imaging Procedures S−12](#_Toc219711979)

[Figure S3 S−13](#_Toc219711980)

[Confocal Visualization of ZnPC Vesicle Internalization S−13](#_Toc219711981)

[Evaluation of Cell Membrane Integrity S−14](#_Toc219711982)

[Evaluation of Cell Membrane Adhesion on the Electrode S−14](#_Toc219711983)

[Confocal Colocalization for Cytomembranal Visualization S−14](#_Toc219711984)

[Cell Counting S−15](#_Toc219711985)

[Evaluation of Cellular Cytotoxicity S−15](#_Toc219711986)

[Statistical Analysis S−15](#_Toc219711986)

[2. Supplementary Notes S−16](#_Toc219711987)

[Peak Picking in ^1^H-NMR Spectrograms S−16](#_Toc219711988)

[Deducing the Depth of Phospholipid Films S−16](#_Toc219711989)

[Explanation on ECL Cascading Coreactions S−16](#_Toc219711990)

[Pearson’s Correlation Coefficient Computation S−18](#_Toc219711991)

[3. Supplementary Figures S−](#_Toc219711992)19

[Figure S4 S−19](#_Toc219711993)

[Figure S5 S−21](#_Toc219711994)

[Figure S6 S−22](#_Toc219711995)

[Figure S7 S−23](#_Toc219711996)

[Figure S8 S−24](#_Toc219711997)

[Figure S9 S−24](#_Toc219711998)

[Figure S10 S−24](#_Toc219711999)

[Figure S11 S−25](#_Toc219712000)

[Figure S12. S−25](#_Toc219712001)

[Figure S13 S−26](#_Toc219712002)

[Figure S14 S−26](#_Toc219712003)

[Figure S15 S−26](#_Toc219712004)

[Figure S16 S−27](#_Toc219712005)

[Figure S17 S−27](#_Toc219712006)

[Figure S18 S−27](#_Toc219712007)

[Figure S19 S−28](#_Toc219712008)

[Figure S20 S−28](#_Toc219712009)

[Figure S21 S−28](#_Toc219712010)

[Figure S22 S−29](#_Toc219712011)

[Figure S23 S−29](#_Toc219712012)

[Figure S24 S−29](#_Toc219712013)

[Figure S25 S−30](#_Toc219712014)

[Figure S26 S−30](#_Toc219712015)

[Figure S27 S−31](#_Toc219712011)

[Figure S28 S−31](#_Toc219712012)

[Figure S29 S−31](#_Toc219712013)

[Figure S30 S−32](#_Toc219712014)

[Figure S31 S−33](#_Toc219712015)

[Figure S32 S−34](#_Toc219712015)

[4. Supplementary Tables S−35](#_Toc219712016)

[Table S3 S−35](#_Toc219712017)

[Table S4 S−36](#_Toc219712018)

[Table S5 S−37](#_Toc219712019)

[Table S6 S−39](#_Toc219712020)

[Table S7 S−40](#_Toc219712021)

[Table S8 S−41](#_Toc219712022)

[Table S9 S−42](#_Toc219712023)

[Table S10 S−43](#_Toc219712024)

[Table S11 S−44](#_Toc219712025)

[Table S12 S−45](#_Toc219712026)

[Table S13 S−46](#_Toc219712027)

[Table S14 S−47](#_Toc219712028)

[References S−48](#_Toc219712029)

**1. Supplementary** **Experimental Details**

**Chemicals and Materials**

(2,4,6-tri(butoxy/octyloxy/dodecyloxy)benzene-1,3,5-triyl)trimethanamines (^4/8/12^TbTm) were fetched from Jilin Chinese Academy of Sciences-Yanshen Technology Co., Ltd. (Changchun, China). Immortalized cervical cancer cell (HeLa), trypsin, and 3-(4,5-dimethylthiazol-2-yl)-2,5-diphenyltetrazolium bromide (MTT) were offered from KeyGEN Biotech Co., Ltd. (Nanjing). Fetal bovine serum (FBS), and dulbecco’s modified eagle medium (DMEM) were procured from Gibco through Life Tech. (New York, USA). Meso-tetra(*p*-formylphenyl)porphine (TFPP), and 2-chloro-2-oxo-1,3,2-dioxaphospholane (CODP) were purveyed by Huawei Ruike Chemical Tech. (Beijing).

Bovine serum albumin (BSA), chloride (CaCl_2_, CuCl_2_, MgCl_2_, NaCl, NH_4_Cl, ZnCl_2_), dimethyl sulfoxide (DMSO), *n*-octyl alcohol, potassium carbonate (K_2_CO_3_), tetrahydrofuran (THF), trichloromethane (CHCl_3_), trifluoroacetic acid (CF_3_COOH), and trioctylamine were purchased from J&K Chemical (Shanghai). Melittin, ibuprofen, and Calcein-AM (calcein acetoxymethyl ester) were acquired from Aladdin and Maclin Biochemical Technologies (Shanghai), respectively. 2,5-Dihydroxybenzoic acid (DHB), cholesterol (Chl), and phospholipids (Table S1) were supplied by Shanghai Yuanye Biotechnology, Pengsheng Biotech and Sigma-Aldrich, respectively. DiSC3(5) (3,3’-dipropylthiadicarbocyanine iodide) was bought from Adamas Reagent Co., Ltd. (Shanghai, China). Mica sheets ($d$ = 10 mm) were sourced from Chemxyz Bio-technology (Shanghai).

Human beta-amyloid 1-42 polypeptide (Aβ_42_, Catalog No. PE-002, Mw = 4.5 kDa), together with its mouse-*anti*-human monoclonal primary (Ab_1_ the capture, KC-106) and paired reporter antibody (Ab_2_, KC-523); recombinant human tau protein (τ, PP-11309, 47 kDa) and its mouse-*anti*-human monoclonal Ab_2_ (KC-116); carcinoembryonic antigen (CEA, PP-102, 12.5 kDa), hemoglobin (HGB, PP-11716, 11.5 kDa), recombinant human immunoglobulin G protein (IgG, KC-001), and human serum albumin (HSA, PP-11214, 32.0 kDa) were obtained from Absea Science and Technol (Beijing). Enzyme-linked immunosorbent assay (ELISA) kits for Aβ_42_ (MM-82685O2) and τ (MM-2260H2) were provided by Mei-Mian Industrial (Yancheng, China). Synthetic oligonucleotides (Table S2), Alexa Fluor 647-conjugated goat-*anti*-mouse IgG (Ab_2_, D110109), monoclonal mouse *anti*-Rab5A (Ab_1_, D194703), and 10X antibody diluent were ordered from Sangon Biotechnol. (Shanghai). Saponin was purchased from Betoytime Biotechnol. (Shanghai). Glutathione-capped gold nanoclusters (AuNCs, ~40 nm) were custom-fabricated by XF-Nano Science and Technology (Nanjing). Mag-fluo-4 acetoxymethyl ester (Mag-Fluo-4) and rhodamine B hydrazide were purchased from Shanghai Maokang Biotechnology.

Unless otherwise specified, all chemicals are of analytical grade and used as received. A 0.1 M 4-(2-hydroxyethyl) piperazine-1-ethanesulfonic acid (HEPES) buffer, prepared in ultrapure H_2_O (≥18.2 MΩ·cm) containing 0.3 M KCl as the supporting electrolyte, was employed for all assays; ultrapure water was produced using an EPED Plus-E3 TS Purification system. Additional buffers included tri(hydroxymethyl)aminomethane (Tris)-HCl and phosphate buffered saline (PBS, 0.1 M, pH 7.5).

**Table S1.** Phospholipid Selections and Structural Schemes Employed in This Study

| Full Name | Short Name | Structure |
| --- | --- | --- |
| 3-palmitoyl-2-oleoyl-*sn*-glycero-1-phosphocholine | POPC | 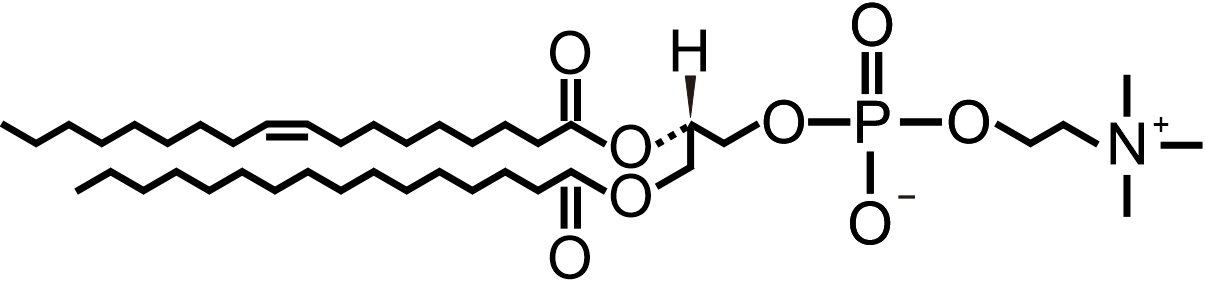 |
| 1-palmitoyl-2-oleoyl-*sn*-glycero-3-phospho-L-serine | POPS | 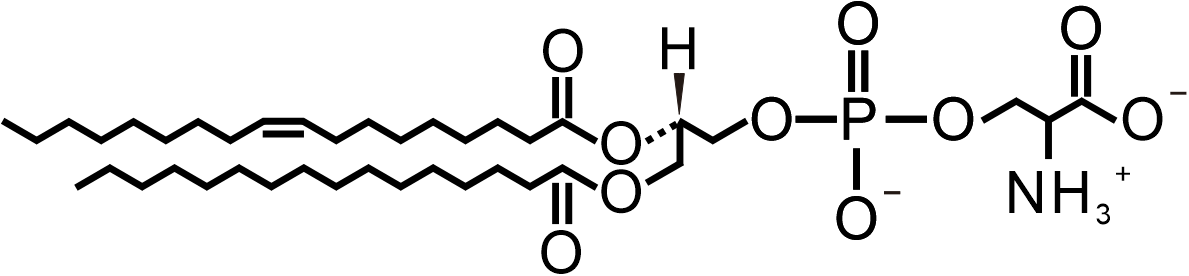 |
| 1,2-dipalmitoyl-*sn*-glycero-3-phosphocholine | DPPC | 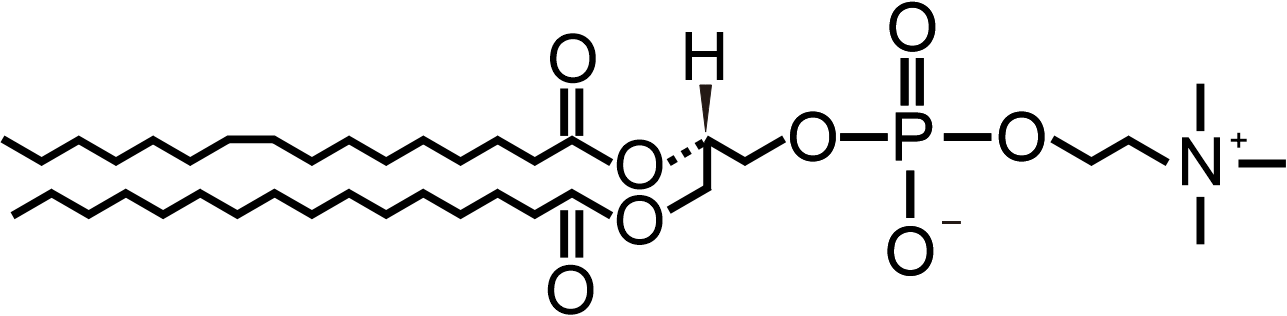 |
| 1,2-dipalmitoyl-*sn*-glycero-3-phospho-L-serine | DPPS | 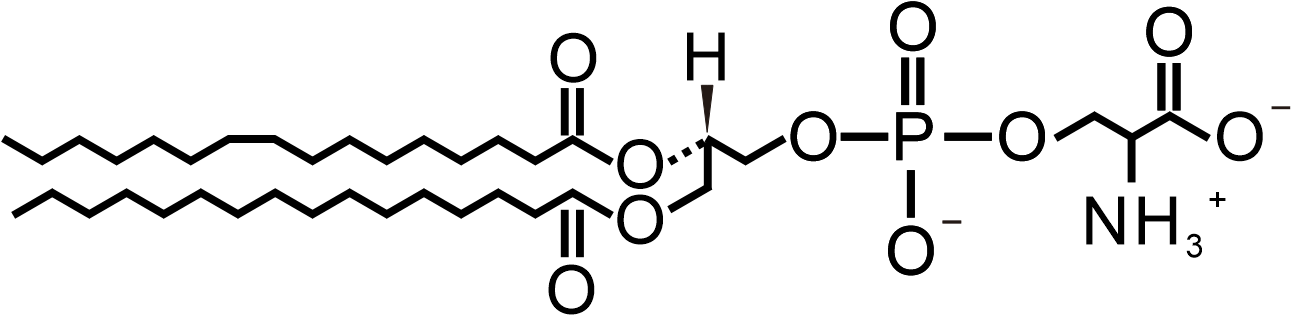 |
| 1,2-dioleoyl-*sn*-glycero-3-phospho-L-serine | DOPS | 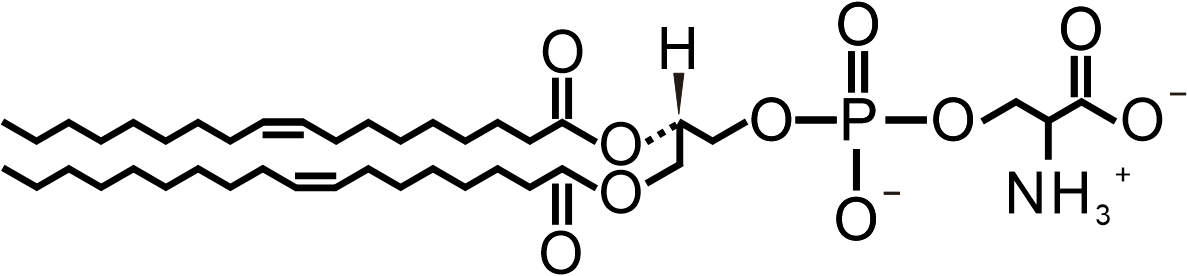 |
| 1-palmitoyl-2-(12-((7-nitro-2-1,3-benzoxadiazol-4-yl)amino)dodecanoyl)-*sn*-glycero-3-phospho-L-serine | NBD-PS | 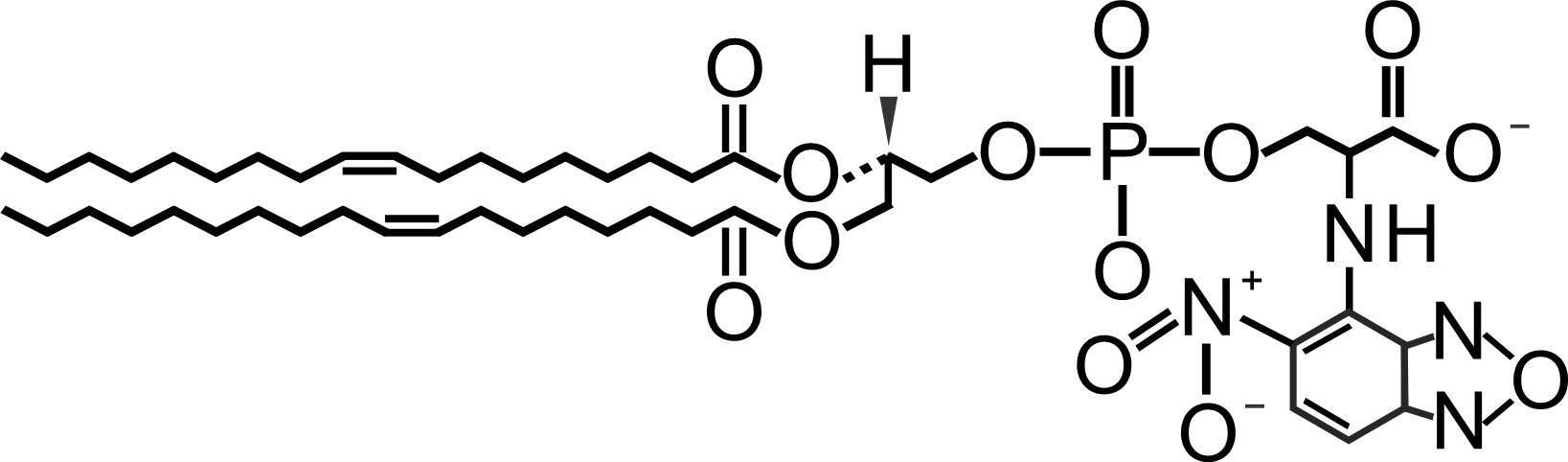 |
| 1,2-distearoyl-*sn*-glycero-3-phosphoethanolamine - polyethylene glycol 2000 - *N*-hydroxy succinimide | DSPE-PEG_2000_-NHS | 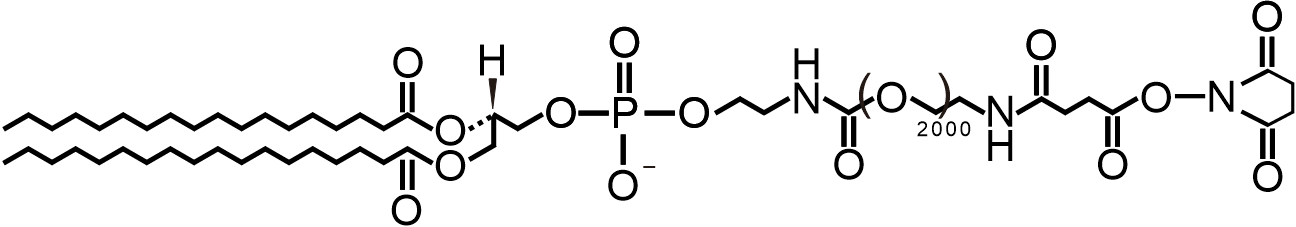 |

**Table S2.** Oligonucleotide Sequences Utilized in This Study

| Strand | Sequence (from 5′ to 3′) |
| --- | --- |
| tau aptamer  (τ-apt) | NH_2_−(CH_2_)_6_−CAG CAC CGT CAA CTG AAT AAG GAC TGC TTA GGA TTG CGA TGA TTC AGG GTG ATG CGA TGG AGA TGT |

**Instrumentation**

Field-emission environmental scanning electron microscopy (FE-ESEM) was conducted with an FEI Quanta 250 FEG microscope (Zeiss, German; acceleration voltage: 5.0 kV), and size statistics in ImageJ (v1.8.0.112, National Institute of Health, U.S.). Ultraviolet-visible (UV-Vis) absorption and Photoluminescence (PL) emission spectrogram were garnered from a SpectraMax M3 Multimode Microplate Reader (Molecular Devices, U.S.). Dynamic light scattering (DLS) sizing as well as zeta potentiometry was performed on a Zetasizer Nano ZS90 Analyzer (Malvern Panalytical, U.K.). Mass spectrometric and Nuclear magnetic resonance (^1^H-NMR) profiles were probed on an Ultraflextreme MALDI-TOF (i.e., matrix-assisted laser desorption/ionization time-of-flight mass spectrograph, 5 mg/mL lipids and DHB the matrix in 7/3 (v/v) CH_3_CN/H_2_O and 0.1 vol.% CF_3_COOH), and an Avance III 500-MHz Spectrometer (Bruker, German), respectively. Static surficial hydrophilicity at equilibrium was estimated on a JYC-series contact angle goniometer (Shanghai FangRui Instrument). (Small-angle) X-Ray diffractometry (SA-XRD) were obtained on a Bruker D8 Advance diffractometer. Atomic force microscopic (AFM) topography was attained from a Bruker Multimode-8 Microscope equipped with ScanAsyst-Air tips operated in tapping mode (spring constant: 0.4 N/m, scan range: 1×1 μm^2^, 512 pixels per line, scan rate: 2 Hz).

Electrochemistry and Chemiluminescence experimentations were executed on a CHI660E workstation (Cheng-hua Instruments, China) and an MPI−EII Client System (Xi’an Remex; photomultiplier tube (PMT) bias: −1000 V, bandpass: 230~920 nm; magnification: 3), respectively. The standard three-electrode configuration comprised a glassy carbon working electrode (inner Φ 5 mm, AIDA Hengsheng, Tianjin), a Pt-wire counter, and an Ag/AgCl reference (fabricated by the Nanjing Institute of Soil Science, Chinese Academy of Sciences) assembled in a cylinderical cuvette (Tianjin Incole Union, 10 mL). Electrochemiluminescence (ECL) spectrometry was scanned using an array of narrowband filters (±10 nm, 25-mm depth×35-mm diameter, 95% transmittivity, Shenzhen Gengxu Optoelectronics). Unless otherwise specified, the scan rate remained at 100 mV/s by default. Electro-chemical impedance spectroscopy (EIS) was studied in 0.3 M KCl containing 5 mM Fe(CN)_6_^3/4−^ at an open-circuit potential of −0.2 V over frequencies ranging from 10^−2^ to 10^5^ Hz, with the equivalent circuit diagram drawn by ZSimDemo v3.30d at www.echemsw.com.

For solution saturation, pure nitrogen (N_2_) or oxygen (O_2_) was overflown out of an Alicat Scientific mass flow controller (MFC, Tucson, AZ) at a steady speed of 5 sccm (standard cubic centimeters per minute). To dictate the dissolved oxygen (D.O.) level, the partial pressures ($p$) of N_2_ and O_2_ were regulate respectively via a pair of MFC flowmeters, whose outlets were attached to an anti-backflow check valve. Gas streams in such bifurcation blended at a T-junction and brimmed the ECL cell thought pipelines, as the block diagram depicts in Figure 2g inset. The overall flow rates, $\upsilon$, of N_2_ and O_2_ was retained at 10 cm^3^/min, with partitioned $\upsilon$_O2_ and $\upsilon$_N2_ in complementary ratios of 0/10, 2/8, 4/6, 6/4, 8/2, and 10/0, which was justified by their tantamount thermal conductivity, $\kappa$, according to Eq. S1:^[1]^

| $\kappa$ = $n$·$\upsilon$·$l$·$c$_V_ / 3*N*_A_ | **(S1)** |
| --- | --- |

where $n$ denotes the number density of molecules, $l$ the mean free path length, and *N*_A_ the Avogadro constant; the conversion factor ($f$) is defined as the specific heat capacity $c$_V_ (N_2_: 0.2485, O_2_: 0.2193 cal/g·K) multiplied by the density $\rho$ (N_2_: 1.250, O_2_: 1.427 g/L, at 0 ºC). By normalizing $f$_N2_ to 1.00, $f$_O2_ = 0.993, thereby confirming the equivalence between $\upsilon$_O2_ and $\upsilon$_N2_. Further taking "the Ideal Gas Law" and "Dalton’s Law of Partial Pressures" into account,^[2]^ we have:

| $\chi$_O2_ = $p$_O2_ / ($p$_O2_ + $p$_N2_) = $n$_O2_ / ($n$_O2_ + $n$_N2_) = $\upsilon$_O2_ / ($\upsilon$_O2_ + $\upsilon$_N2_) | **(S2)** |
| --- | --- |

where $\chi$_O2_ means the mole fraction of O_2_. Notably, $n$ is proportional to the volumetric velocity $\upsilon$, given that the molar volume of an ideal gas is constant at 22.4 L/mol. After each electrode test, the D.O. value was evaluated using an oximeter (Seven2Go S9, Mettler Toledo, LLC., Columbus, OH).

**Fabrication of Biofilm-Fusable ECL Emitters (ZnPC)**

First of all, TFPP (C_48_H_30_N_4_O_4_, Mw: 726.8) was prepared in compliance with the conventional acid-catalyzed four-fold cyclization between aldehyde and pyrrole.^[3]^ The product was purified by Agilent 1260 Infinity II Prime liquid chromatography (Santa Clara, CA) and desolvated via rotary evaporation (>95%, N-1100, Eyela Instrument, Shanghai). Next, to 20 mL of CHCl_3_ pre-dissolving TFPP (36 μM, 27 mg) and TbTm (48 μM, either ^4^TbTm: C_21_H_39_N_3_O_3_, Mw 381.6, 18 mg; ^8^TbTm: C_33_H_63_N_3_O_3_, 549.9, 26 mg; or ^12^TbTm: C_45_H_87_N_3_O_3_, 718.2, 34 mg), 2.2 μmol CF_3_COOH in 1 mL of THF was melded, and the mixture underwent stirring for a 14-h imine condensation at room temperature. Afterwards, the organic phase was progressively extracted against saturated NH_4_Cl, K_2_CO_3_, and brine, followed by anhydrous K_2_CO_3_ powder. Upon processing in a vacuum oven at 60 °C, the porphyrin cage (correspondingly, ^4^PC: C_456_H_444_N_48_O_24_, Mw 6980.7; ^8^PC: C_552_H_636_N_48_O_24_, 8316.0; and ^12^PC: C_648_H_828_N_48_O_24_, 966-0.0) as an azomethine compound was crystallized as a dark-violet solid (productivity: 95%). Thence, its hollow center ensued metalation with excess ZnCl_2_ (mole ratio >1:8) in THF under gentle agitation at 50 ℃ for 2 days in the dark. Recurring to the foregoing refinements, it would deliver dry ZnPCs (Zn^4^PC: Mw 7361.0; Zn^8^PC: 8707.4; Zn^12^PC: 10053.8) that shall be stored in shade prior to ulterior utilization. Zn^4^PC is abbreviated as ZnPC in most cases. The net reaction is rendered in Figure S1.


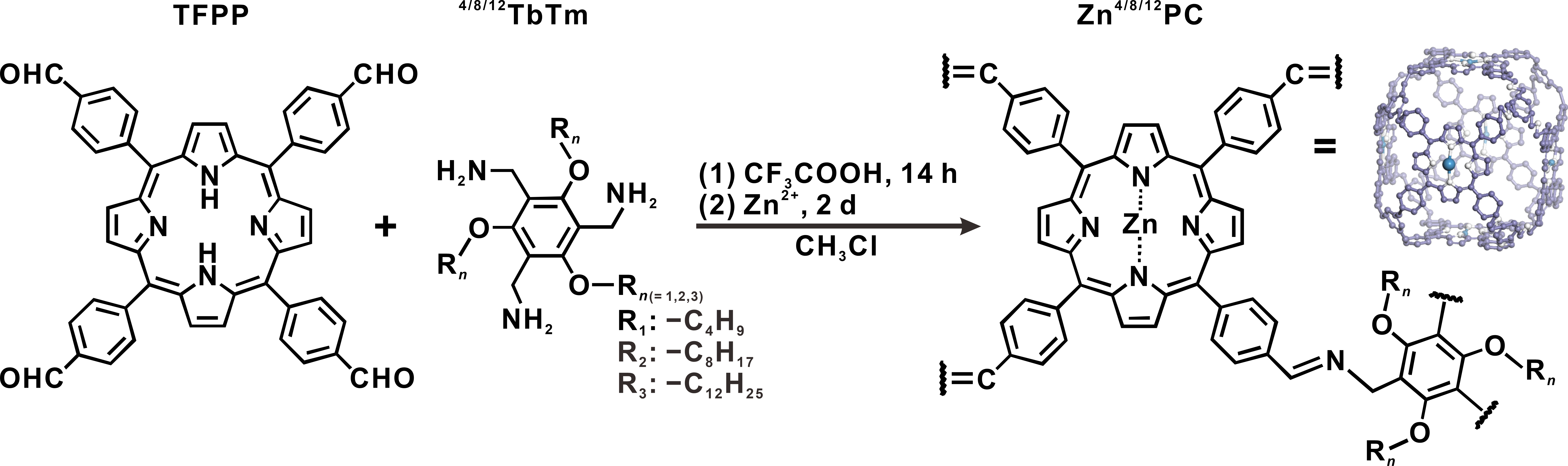


Figure S1. Synthetic scheme depicting construction of Zn*^n^*PC (*n* = 4, 8, 12) through dynamic C=N coupling. The right panel presents a space-filling scaffold of a microcube assembled from 6×face-centred ZnTFPP and 8×tripodal *^n^*TbTm. Peripheral alkoxy chains and hydrogens are purposefully suppressed for structural succinctness.

**Preparation of Small Unilamellar Vesicles and Supported Lipid Bilayers**

First, small unilamellar liposomes were secured via the freeze-thaw and extrusion method. 3.2 mg/mL POPC was concocted in chloroform, and the container was changed to a scintillation vial for N_2_ ventilation to vaporize the volatiles. The lipids were desiccated and deposited at the bottom for at least an additional 2 h of residual removal in a depressurized desiccator, equipped with an SHZ-D oil pump (0.0974 MPa, Yingyu High-Tech, Guangzhou). The lipids were then rehydrated in PBS (100 mM NaCl, 10 mM Na_2_HPO_4_) at pH 7.4 to a concentration of 1 mg/mL, and subjected to bath sonication for 3−5 min to detach adherent lipids completely. Thereafter, the homo-genous phase was submitted to ten alternating cryo-thaw cycles in liquified N_2_ and warm water at 40 °C; thereby the vesicles were extruded using a pneumatic extruder (AE001, 0−1000 psi, ATS Engineering, Israel) through a polycarbonate paper with 200 nm pores (Whatman, Florham Park, NJ), with the process repeated ten times. These vesicles were freshly fabricated before use.

Second, following depositing a 10 μL sample onto freshly cleaved mica and incubating at room temperature for 30 min, during which the vesicles adhere to the surface and spontaneously ruptured, spreading and fusing to form a continuous bilayer. After incubation, the vesicle suspension was carefully aspirated from the mica surface using a pipette. Then the mica monosheet was rinsed with PBS (pH 7.4) by slowly syringing the buffer multiple times with a pipette. Last, the sample was imaged using a Bruker Multimode-8 AFM. To precisely parameterize the membrane morphology, roughness values ($R$_q_) were evaluated from five random regions of interest (ROI, 50×50 nm^2^) on the smooth surface, rather than the entire picture incorporating steep steps. This prevents the macro-topographic altitudes from interfering the micro-roughness measurements. All topographic images were subjected to a first-order flattening filter to subtract substrate tilt prior to quantitative analysis.

**Preparation of** **(2-(Trioctylammonio)ethyl)octylphosphate (TOAP)**

By analogy to tetra(*n*-octyl)ammonium bromide (TOAB),^[4]^ an efficient eutectic film-former for ECL-emissive porphyrins, a zwitterionic phospholipoid was prepared using cationic tri(*n*-octyl)amine as the headgroup.^[5]^ As illustrated in Figure S2, its synthesis starts with the production of precursor, alkylated dioxaphospholane oxide, via esterification of CODP with an aliphatic alcohol. Specifically, CH_3_(CH_2_)_7_OH (3.91 g, 30 mmol) and N(CH_2_-CH_3_)_3_ (4.27 g, 30 mmol) were combined in 25 mL of anhydrous THF. Thenceforward, CODP (30 mmol) dissolved in 10 mL of THF was delivered dropwise at 0 °C. Thereafter, the commixture was maintained at 25 °C for 12 h. Separating superfluous triethylamine hydrochloride by filtration, the filtrate was subjected to rotary evaporation under reduced pressure to produce the intermediate (6.17 g, yields: 87%).

In the second step, ionizable TOAP was generated through a ring-opening orthogonal addition between trioctyl-amine (6.00 g, 17 mmol) and octyl dioxaphospholane oxide (6.60 g, 28 mmol) dissolved in 20 mL DMSO at 70 °C for 3 days, followed by *in vacuo* evacuation of DMSO to obtain a pale-yellow oily liquid (7.21 g, yield: 72%).


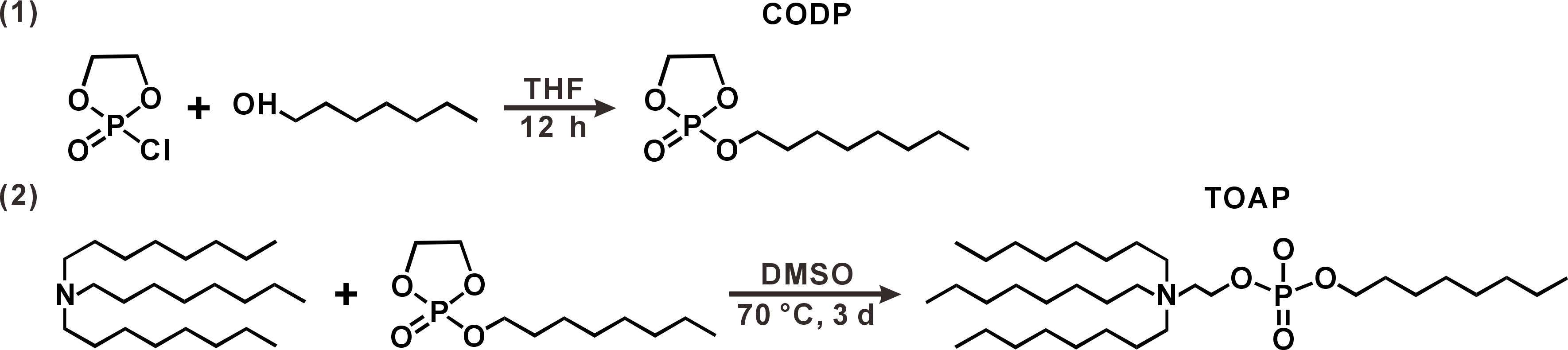


Figure S2. Stepwise streamlined synthesis of TOAP, structurally similar to TOAB, serving as an artificial amphiphilic phospholipid for polarity-permeability modulation of lipid bilayers.^[6]^

**Preparation of ZnPC-Invasive Vesicles**

Similar to the above-stated steps, this also started with small unilamellar liposomes from the freeze-thaw and extrusion.^[7]^ POPC (3.2 mg/mL) and Chl (0.4 mg/mL) were co-dissolved in chloroform, and the container was transferred to a scintillation vial for N_2_ ventilation to evaporate the volatiles. The lipids were dried and dwelled at the bottom for further ≥2 h remnant removal in a depressurized desiccator with an SHZ-D oil pump (0.0974 MPa, Yingyu High-Tech, Guangzhou). The lipids were then rehydrated in PBS (100 mM NaCl, 10 mM Na_2_HPO_4_) at pH 7.4 to a concentration of 1 mg/ mL, and brought into bath sonication for 3−5 min in order to desorb all adherent lipids. Next, the homogenous phase was put through 10 alternate cryo-thaw cycles in liquified N_2_ and warm water at 40 °C; thereby the vesicles were exuded by a pneumatic extruder (AE001, 0−1000 psi, ATS Engineering, Israel) across a polycarbonate paper with 200-nm pores (Whatman, Florham Park, NJ), which were repeated ten times.

For generating ZnPC-invaded vesicles, 20 μM ZnPC was supplemented to the solution, while other components stayed the same. The exception is that all ZnPC-carrying vesicles, such as ZnPC@POPC/Chl and ZnPC@POPC/ POPS/Chl, only one-round extrusion was exerted to elude the depletion of ZnPCs during excessive extrusions. These vesicles were freshly fabricated before use.

**Fluorescence Recovery After Photobleaching (FRAP)**

For FRAP experimentation, add 1 mol.% NBD-POPS and adhere to the rest routines for ZnPC-invasive vesicles. FRAP experiments were exercised with a T*i*2-U inverted epifluorescence microscope (Nikon, Japan) equipped with a DS-Qi2 sCMOS camera (Nikon, Japan) for fluorescent frame formation. To reinforce the resolution, an oil immersion objective with a high numerical aperture was picked (40X, Plan, Fluor ∞/−, N.A. 0.17, W.D. 0.66). One channel was chosen to capture the photoluminescence of NBD-PS lipid (incident light: 488 nm; emergent slit: 500−550 nm). Photobleaching was begot using a 405-nm diode laser (MDL-III-405, 50 mW, Changchun New Industries Optoelectronics Tech. Co., Ltd., China) at maximum output power. FRAP studies were successfully secured for more than three rounds on different days and lipid domains.

The fluorescence intensity ($I$_FL_) within the photobleaching spot was first scaled by a nonphotobleached space, and then normalized to the mean magnitude of $I$_FL_ measured prior to photobleaching. The lipid diffusivity was derived from the FRAP readout by:^[8]^

| $D=\frac{r_{\text{n}}^{\text{2}}+r_{\text{e}}^{\text{2}}}{\text{8}\tau_{\text{1/2}}}$ | **(S3)** |
| --- | --- |

where $D$ (μm^2^/s) is the diffusion coefficient and $\tau$_1/2_ (s) is the half time of recovery. $r$_n_ (μm) and $r$_e_ (μm) are the radii of the resulting round region and the effective radius after bleaching, respectively. Thus, we separately assessed $\tau$_1/2_ from FRAP trajectories and $r$_e_ from the fluorescence figures, and then calculated the lipid diffusivity by Eq. S3.

**Electrode Surface Functionalization and Membrane Interaction Investigations**

Pieces of bare glassy carbon electrode (GCE, inner Φ 5 mm) were abraded against an appropriate amount of 0.3, 0.05 μm γ-alumina slurries (Lab Testing Tech., Shanghai) on the suede surface using a motorized mortar (Gaoss-Union Photoelectric, Tianjin) until a mirror finish. Thenceforth, the electrode bodies were decontaminated in a high-power ultrasonic cleaner (Kunshan Ultrasonics, Suzhou) through successive baths of absolute alcohol and deionized H_2_O. After being blown dry with N_2_ blasts, the electrodes were set aside for subsequent surface film formation.

Chloroform containing 6.4 mg/mL POPC and 0.8 mg/mL Chl was amalgamated and agitated with 40 μM ZnPC at an equivoluminal ratio of 1:1. The pretreated GCE substrate was syringed with 10 μL of ZnPC@POPC/Chl, and allowed to evaporate CHCl_3_ for 10 min. The immobilized interface denoted as "GCE/ZnPC@POPC/Chl" was then immersed in 2 mL of 0.1 M HEPES (pH 7.5) for ECL examination. For the SEM scrutiny, samples at semi-volumes (5 μL) were dispensed onto detachable GCE headpieces (Φ 2.5 mm, GaossUnion). For AFM altitudinal assessment, a 5 μL aliquot was likewise syringed onto a GCE headpiece and allowed to adsorb for 3 min, then mildly trickled with 200-μL ultrapure H_2_O and drying under N_2_.

For lipid-bilayer binding titrations, divalent cations Ca^2+^ and Mg^2+^ (0.001, 0.01, 0.1, 1, 5, 10, 15 mM), Cu^2+^ (0.5, 1, 2.5, 5, 10, 50, 100 μM), melittin (1, 2, 3, 4, 5, 10, 25, 50, 75, 100 μM), and ibuprofen (0.05, 0.1, 0.5, 1, 1.5, 2, 3 mM) were all aliquoted in 0.1 M HEPES (pH 7.4). Cyclic voltammetry was employed as the stimulus, with a scan rate of 100 mV/s over a sweep window between 0 and −1.45 V. On the other hand, the Ca^2+^/Mg^2+^-selective Mag-Fluo-4 and Cu^2+^-selective rhodamine B hydrazide were utilized to elucidate the membrane processes of these species. Adhering to a differential method, dyes were introduced independently and equimolarly at 20 μM into the HEPES buffer either before or after the ECL experiments, sampled at a same amount, and subjected to the spectrofluorometry at excitation ($\lambda$_ex_) and emission ($\lambda$_em_) wavelengths of $\lambda$_ex_/$\lambda$_em_ [Mag-Fluo-4] = 494/516 nm, and $\lambda$_ex_/$\lambda$_em_[Rhodamine B hydrazide] = 510/578 nm (sampling interval: 1 nm).

**Wide-Field Full-Electrode ECL Visualization**

A thin-layered electrolyzer for in situ Raman scattering spectroscopy (No. 031-4, 1 mL, 25-mm-wide window, 6-mm from an inverted glassy carbon disk (GCD, Φ 2.5 mm), GaossUnion Photoelectric, Tianjin) was adapted for wide-field ECL imaging in a Tanon-520 Multi Automatic Gel Transilluminator (Shanghai) with a Teledyne Photo-metrics PI6500 CCD (charge-coupled device, Tucson, U.S.) camera (cryogenic at −40 °C, resolution: 6 mega-pixels (2750×2200), target plane: 2/3 inches (11×9 mm^2^)).

A droplet of ZnPC@POPC+Chl (20 μL CHCl_3_ containing 20 μM ZnPC, 3.2 mg/mL POPC, and 0.4 mg/mL Chl) was cast onto the cell-compatible GCD and sat stationary to evaporate the solvent for 10 min. The operando Ra-man chipset was assembled in an airproof fashion wherein a microfluidic chamber was fully infused with 1mL debubbled buffer, and positioned on the shooting plate inside the dark box. The three-electrode bundle was linked via alligator clips to a CHI660E workstation (Chenghua Instruments Co., Ltd.) externally.

ECL exposures (5 min within 18 s of effective ECL-active exposure, AllDoc_x v2.2.1.0 (**a**)) was synchronized with cyclic voltammetry (Electrochemical Soft-ware, v20.04 (**b**), typical preset: Initial Potential = 0 V, Final Potential = −1.50 V) via a Windows 10 (**c**) scripter (Anjian, v2014.06.19549) for orchestrated startups and timing alignment. The on-demand moves of mouse cursor were automated as arranged in the timetable below. The ECL light-spots were snapshotted and stacked in albums for post-processing using TanonImage (v1.00) and ImageJ (v1.54f), through which panelled photos were digitized in terms of pixelate-averaged ECL intensity ($I$_ECL_, 1 pixel ≈ 1.3 μm). To bespeak batch uniformity, the chamber was purged and replenished with highly pure nitrogen between acquisitions.

| Step Sequence | Mouseover to Desktop Coordinate  ($x$, $y$) | Hover Latency  (ms) | Left-Click Times |
| --- | --- | --- | --- |
| **1** | (**b**): (181, 55) | 1000 | 1 |
| **2** | (**c**): (654, 1055) | 1000 | 1 |
| **3** | (**a**): (1870, 299) | 1000 | 1 |

**ECL Biosensor Build Based on Lipid Layers for Alzheimer’s Marker Measurements**

Stepwisely, (1) a formulation was fashioned by blending 20 μM ZnPC, 3.2 mg/mL POPC, 0.4 mg/mL Chl, and 0.0016 mg/mL DSPE-PEG_2000_-NHS. Succedently, 20 μL of the blended bulk was drop-deposited onto a bare GCE to afford ZnPC@POPC/DSPE-PEG-NHS/Chl. (2) Typically, 10 μL of 0.5 mg/mL Aβ_42_-Ab_1_ or tau-aptamer was adsorptively anchored via amidation between the −COOH terminus and *N*-hydroxysuccinimide sites for ~70 min; afterward, 10 μL of 0.5 wt.% BSA was introduced to blocked nonspecific occupancies. A 10 mM PBS (pH 7.4) was wielded for washing. (3) Graded gradients (0.01, 0.1, 1, 10, 100, 1000, 10000 ng/mL) of Aβ_42_ or tau standard substances were transferred to the recognition region and retained to react over 30 min. (4) After rinsing residual antigens, an adequate amount of AuNC-conjugated Aβ_42_-Ab_2_ or tau-Ab_2_ (10 μL, 0.1 mg/mL) was pipetted and positioned onto the preassembled hapten layer to set up a sandwich-typed immunocomplex. These four procedures constituted the complete workflow.

Herein, labeling Ab_2_ with AuNCs was enacted via electrostatic attraction.^[9]^ Specifically, the pH of the AuNCs dispersion (100 μL, 0.5 mg/mL) was adjusted and alkalized to 9.0 using K_2_CO_3_. Ulteriorly, 30 μL of 1 mg/mL Aβ_42_-Ab_2_ or tau-Ab_2_, 100 μL of 0.1 mg/mL PEG_4000_, and 100 μL of BSA (final content 0.5 wt.%) were filled in, followed by vortexing for 1 h. The suspension was centrifuged to collect Aβ_42_-Ab_2_@AuNC or tau-Ab_2_@AuNC nanolabels at 7500 rpm for 6 min, with the supernatants decanted. The collected pellets were then redispersed and resuspended in 100 μL of PBS (pH 7.4).

**Cell Culture and Single-Cell ECL Imaging Procedures**

HeLa cells were incubated in accordance with the approach in the literature.^[10]^ Briefly, 5 mL DMEM along with 10 wt.% FBS were recruited to culture cells in an incubator (MCO-18AC, PHCbi Health Medical Devices, Japan) under a humidified milieu (5 vol.% CO_2_+95 vol.% air) at 37 °C. Upon entering the logarithmic growth period, the medium was aspirated away, while cells were rinsed thrice with D-PBS. Subsequently, 2 mL of 0.1 wt.% trypsin was trickled onto the dish to digest cells for 2 min. The proliferated cells were centrifuged at 800 rpm for 5 min, purified with D-PBS for three rounds, and seeded onto an indium tin oxide (ITO, 2.6×0.3×0.1 cm^3^, Fisherbrand) glass slide for 12 h of surface settling. Herein, all ITOs were precleaned in a near-boiling 7X detergent dilution (1:7 v/v in ultrapure H_2_O, MP Biomedicals, LLC., Solon, OH) for 2 h on ceramic supports, swilled thoroughly with double-distilled H_2_O, and swiftly dried with N_2_. The resulting smooth surface furnished a cell-compatible context. Finally, vesicles involving 500 μM POPC, 200 μM Chl and 5 μM Zn^8^PC were co-incubated with cells for 60 min. Prior to ECL examinations, 5 mL of PBS (0.1 M, pH 7.4) was sucked in to substitute DMEM in the matrix.

Single-cell morphology was monitored on a homemade ECL imager (Figure S3), which was modularly integrated with a T*i*2-U inverted PL microscope (Nikon, Japan), an electron-multiplying charge-coupled device (EMCCD, iXon Ultra 897, Andor, U.K.), a CHI660E electrochemical workstation, as well as a customized electrolytic cell (polystyrene, 90% transmittance, 0.8×0.8×0.7 cm^3^ per compartment (2×4), general geometry: 5.7×2.5×0.8 cm^3^) using ITO (85% transmittance throughout 380−780 nm) as the substrate and also the working electrode, with both the bulky Pt wire and Ag/AgCl still serving as the counter and reference, respectively). The system was stationed in a dark room to suppress stray scattering light interference. The ECL exposures were extended to a cumulative duration of 120 s by voltametric cycling in O_2_-saturated 0.1 M PBS within a dynamic window of [0, −2] V at 0.1 V/s. The 120 s exposure corresponded to the accumulation of three consecutive CV cycles, yielding an effective ECL emission window of 21 s. ImageJ was adopted to analyze the acquisitions via the objective (20X: Plan, Fluor ∞/−, N.A. 0.17, W.D. 2.1; 40X: Plan, Fluor ∞/−, N.A. 0.17, W.D. 0.66). In parallel, fluorescence from ZnPCs was excited by an LED source (excitation filter: 470±40 nm, dichroic mirror: 500 nm, barrier filter: 534±55 nm; C-LED-FIF, Nikon, Japan) with an exposure time of 100 ms.


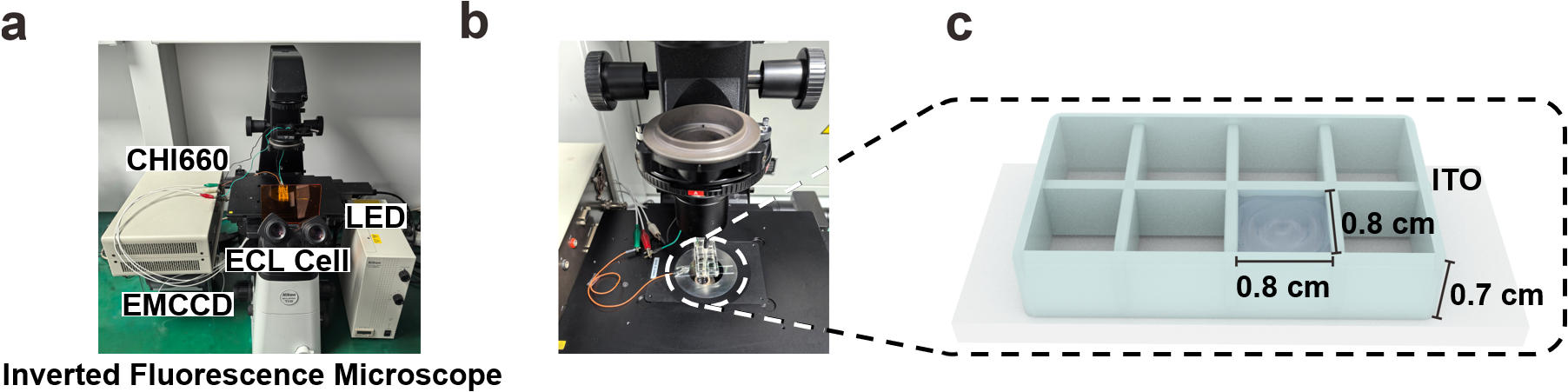


Figure S3. Photographic portrayal of the ECL cell-probing platform. a) Instrumental integration and overall layout. b) Magnified micrograph of the self-assembled ECL electrolyzer. c) Schematic sketch illustrating the ECL cuvette configuration.

**Confocal Visualization of ZnPC Vesicle Internalization**

HeLa cells were incubated and cultured in DEME medium containing 10 wt.% FBS at 37 °C under a 5% CO_2_ atmosphere. Thereafter, the cells were cast off and collected from culture dishes using 2 mL trypsin (0.1 wt.%), followed by centrifugation at 800 rpm for 5 min, and were subsequently resuspended and rinsed in 1×Dulbecco’s PBS (D-PBS) for triple times. Ultimately, the cells were inoculated and immobilized onto a 15-mm confocal dish, and left undisturbed and unstimulated for 24 h. Later, the media including ZnPC-embedded vesicles (0.1, 1, 5 μM) were carefully covered the cartridge and cultivated at 37 ºC for 3, 20, 60, and 180 min, progressively. Proceeding one step further, the cells were washed and withdrawn thrice with 1×D-PBS, and their confocal laser-scanning snapshots were shot and surveyed on an Eclipse T*i*2-E epifluorescence microscope (Nikon, Japan; filter cube: FITC, incident/emergent: 488/525 nm; objective: 63X, Plan Apo, W.D. ∞/0.17, N.A. 1.40, oil immersion; detector: ORCA-Flash 4.0 sCMOS camera, 2048×2048 pixel^2^, Hamamatsu Photonics, Japan) under a 10 s exposure at 1−2 frame per second. Images were inspected and interpreted using NIS-Elements Viewer v5.21 and ImageJ software.

For immunofluorescence staining, cells were fixed with 4% paraformaldehyde for 10 min at ambient temperature and then cleaned thrice with PBS. Residual aldehydes were deactivated with 50 mM NH_4_Cl in PBS for 10 min at room temperature, in addition to two PBS washes. Again at room temperature, cells were permeabilized using the commercially supplied saponin solution (direct and undiluted) for 10 min, and blocked with 1 wt.% BSA in the same saponin for 30 min. The samples were incubated with monoclonal mouse *anti*-Rab5 antibodies (1 mg/mL aliquoted to 200X in blocking buffer) for 1 h. After triple rinses with the permeabilizer, cells were co-incubated with Alexa Fluor 647-conjugated goat *anti*-mouse IgG (0.5 mg/mL into 500X in blocking buffer) for 1 h in the dark, which were then washed three times with PBS. Cell nuclei were counterstained with DAPI (1 μg/mL) for 5 min with double washes. At last, the cells were preserved in PBS for observation. The ZnPC photoluminescence was gathered in the green channel and DAPI in the blue channel. Coupling between Rab5 and mouse *anti*-Rab5 Ab_1_ was visualized with the aid of Alexa Fluor 647-labeled Ab_2_.

**Evaluation of Cell Membrane Integrity**

Cell membrane integrity was assessed and analyzed using the photoluminescent probe DiSC3(5) (3,3'-dipropyl-thiadicarbocyanine iodide). HeLa cells were incubated identically as described in section "Cell Culture and Single-Cell ECL Imaging Procedures". Briefly, the HeLa cells were transplanted and tethered in a homemade ECL cell (1 ×10^4^/well), as illustrated in Figure S3, for 12 h of surface sticking, while vesicles containing and comprising 500 μM POPC, 200 μM Chl, and 5 μM Zn8PC were co-incubated concurrently with the cells for 60 min. Prior to ECL examination, 5 mL of PBS (0.1 M, pH 7.4) was introduced to replace DMEM. This was followed by electro-chemical stimulation for 2 min (scan rate: 0.1 V/s, potential range: [0, −2] V). Subsequently, 1 mL of 5 µM cyto-membrane dye, DiSC3(5) (in PBS) was incorporated and incubated, and the cells were maintained at 37 °C for 20 min. The cells were then washed and withdrawn three times with D-PBS, followed by recording and reading the fluorescence intensity using a SpectraMax M3 Multimode Microplate Reader. Cells omitting ECL treatment served as blank controls. For DiSC3(5), $\lambda$_ex_/$\lambda$_em_ = 622/670 nm.

**Evaluation of Cell Membrane Adhesion on the Electrode**

Cell adhesion stability was assessed and analyzed using a quantitative PL-based resistance-to-detachment assay. Briefly, HeLa cells were transplanted and tethered in a homemade ECL cell (1×10^4^/well), as shown in Figure S3, for 12 h of surface sticking, while vesicles containing and comprising 500 μM POPC, 200 μM Chl, and 5 μM Zn^8^PC were co-incubated concurrently with the cells for 60 min. Next, the homemade ECL cell was secured and shaken on a horizontal shaker at 200 rpm for 5 min. Subsequently, the cells were washed and withdrawn three times with D-PBS to remove detached cells. Then, 1 mL of 5 µM Calcein-AM (Calcein acetoxymethyl ester, in PBS) was incorporated and incubated, and the cells were maintained at 37 °C for 20 min. Untreated cells served as control. The fluorescence intensity was then recorded and read using a SpectraMax M3 Multimode Microplate Reader. For Calcein-AM, $\lambda$_ex_/$\lambda$_em_ = 494/515 nm. The relative adhesion rate was calculated and compared by dividing the fluorescence intensity of the experimental group by that of the control group.

**Confocal Colocalization for Cytomembranal Visualization**

HeLa cells were seeded and sustained in culture dishes at 37 °C for 48 h to reach the exponential growth phase. Thereafter, 0.5 mL of 5 µM ZnPC-fused vesicles in PBS was perfused and propagated into 5 mL DMEM medium, and the cells were fostered and facilitated for 60 min. Subsequently, the medium was discarded and displaced with PBS to remove unbound ZnPC. As a follow-up, 2 mL of 5 µM cytomembrane-binding dye, DilC18(3) (Dil, 1,1'-dioctadecyl-3,3,3',3'-tetramethylindocarbocyanine perchlorate, Macklin Biochem., in PBS) was incorporated and incubated,^[11]^ and the cells were maintained at 37 °C for 20 min. After separating out the staining solution, the cells were washed and withdrawn three times with copious PBS, and visualized using an inverted epifluorescent microscope. For ZnPC, $\lambda$_ex_/$\lambda$_em_ = 428/614 nm; for Dil, $\lambda$_ex_/$\lambda$_em_ = 549/565 nm.

**Cell Counting**

Cytometry was conducted and calculated using the cell suspension after trypsin digestion and deposition as described above, followed by dilution for accurate counting. (1) Load a DS-66 hemocytometer (16.2 L×6.2 W×0.2 H mm^3^, 6 channels, Ruiwode Life Science, Shenzhen) and place a coverslip (30×10×1 mm^3^, Fisherbrand) over the counting chamber; carefully convey a 10−20 μL droplet, allowing it to wet and wick the trough via capillary force. (2) Observe the channel through a 10× objective, gazing upon the grid pattern. (3) Count the cells in the corner and central squares, registering only those touching the top and left borders, not those abutting the bottom or right borders. (4) Compute the cell concentration (cells/mL) = numerated cell number × rarefaction factor × 10^4^.

**Evaluation of Cellular Cytotoxicity**

The cytotoxicity of ZnPCs was assessed and analyzed by a standard MTT (3-(4,5-dimethylthiazol-2-yl)-2,5-diphenyltetrazolium bromide) assay.^[12]^ HeLa cells were transplanted and tethered in a 96-well microplate (1×10^4^ /well) at 37 °C for 18 h, and then treated and incubated with ZnPC-containing vesicles for 3 h, followed by electrochemical stimulation for 2 min (scan rate: 0.1 V/s, potential range: [0, −2] V). Cells omitting ZnPCs served as blank controls, whereas an additional group was subjected to electrochemical stimulation alone for 2 min. Sub-sequently, the cell culture medium was removed and replaced by MTT solution (5 mg/mL) and slowly shaken and sustained at 37 °C for 4 h on a BE-9008 thermoshaker (Kylin-Bell Lab Instruments, Haimen, China). Finally, by dislodging residual dye, 100 μL of DMSO was distributed and dissolved in each well, and the absorbance was recorded and read at 490 nm to calculate cell viability.

**Statistical Analysis**

Unless otherwise specified, no data points were excluded from the analyses. Data are displayed as mean ± standard deviation, unless otherwise stated in the corresponding figure legends. The sample size ($n$) for each experiment is indicated in the corresponding figure legends. Surface roughness of AFM was calculated from randomly selected regions of interest on flattened images by NanoScope Analysis 3.0 (Build R1Sr3.169498). Calibration curves were constructed by linear regression and residual regression was subsequently performed to scrutinize the fitting stability where suitable using Origin Pro 2018 (b9.5.1.195). For single-cell signal quantification and Pearson’s correlation coefficient, data are shown as mean ± standard error of the mean by ImageJ (v1.54s). Statistical significance between two groups was gauged using a two-tailed Student’s *t*-test, and a value of *P* < 0.05 was considered statistically significant. Statistical scrutiny was performed using GraphPad Prism 10 (10.6.0).

**2.** **Supplementary Notes**

**Peak Picking in** **^1^H-NMR Spectrograms**

As aligned in Figure S4, the chemical shifts ($\boldsymbol{\delta}$, in ppm) of the precursor TFPP (a), the tritopic ^4/8/12^TbTm (b, d, f), and the nonmetallic Zn^4/8/12^PC (c, e, g) were denoted and designated in three divisions with definitive labels on the ChemDraw-illustrated insets. By comparison, the pyrrolyl H^a^ of TFPP translocated and transferred to 8.8 ppm in *^n^*PC; concurrently, the benzyl H^b^/H^c^ singlets split into a quartet, both in unison with software simulation. The H^d^ in aldehyde at 10.4 ppm decayed to a side-reaction residual (<5 wt.%); simultaneously, the secondary aldimine (−CH=N−) arose and appeared in conjunction with an obvious move of methylene H^e^ in TbTm, thus affirming the Schiff-base formation in *^n^*PC. In accordance with Dynamic Covalent Chemistry theory,^[13]^ 6×TFPP tie together and terminates into an iconic Archimedean polyhedron body with exactly 8×TbTm, as sketched in Figure S1.

**Deducing the Depth of Phospholipid Films**

Taking the Faraday’s equation of electrolysis and the fundamental formula for capacitors into account,^[14]^ we have:

| $C=\frac{\int i\text{d}E}{\text{2}\upsilon E}=\frac{\varepsilon_{\text{r}}S}{\text{4π}kd}$ | **(S4)** |
| --- | --- |

where $\int i\text{d}E$ represents the integral and incorporated area of a CV loop (1.419×10^−5^) in N_2_-saturated solution; $\upsilon$ denotes the scan rate (0.1 V/s), $E$ the potential range (1.45 V); $\varepsilon_{\text{r}}$ refers to the relative dielectric constant (= 3), $S$, $k$, $d$ correspond to the capacitor plate area (i.e., the surface area of a GCE = 6.25×10^−6^ m^2^), the electrostatic constant (8.98×10^9^ N·m^2^/C^2^), and the distance between the two plates (i.e., the film thickness), respectively.

**Explanation on ECL Cascading Coreactions**

As previously expounded elsewhere,^[15]^ the coreacting route of ZnPC encompasses a complexity of three-state superpositions: anti-bonding π in reduced TFPP^−^ (Eq. S4 and S5), *d*-orbital in Zn^2+^ and *p*-orbital in singlet O_2_^•−^ (Eq. S6−S11). In Figure S8, three redox peaks are denoted and designated, i.e., $E$_P1_ = −0.38 V, $E$_P2_ = −0.73 V, and $E$_P3_ = −1.45 V from zero to negative potentials. Progressively, the complete expressions, excerpted from "ECL PROPERTIES" in Supporting Information of Ref. 14, are formulated as follows:

| $E$_P1_: ZnPC + $e$^−^ $\to$ ZnPC^−^ | **(S5)** |
| --- | --- |
| *E*_P1_: 4ZnPC^−^ + O_2_ + 2H_2_O $\to$ 4ZnPC + 4OH^−^ | **(S6)** |
| $E$_P2_: Zn^2+^PC + $e$^−^ $\to$ Zn^1+^PC | **(S7)** |
| *E*_P2_: O_2_ + Zn^1+^PC $\leftrightarrow$ [O_2_−Zn^1+^]PC | **(S8)** |
| *E*_P2_: [O_2_−Zn^1+^]PC $\leftrightarrow$ [O_2_^•−^−Zn^2+^]PC $\to$ Zn^2+^PC + ^1^O_2_ $\to$ $h\upsilon$_ECL_ ($\lambda$ ≈ 641, 703 nm) | **(S9)** |
| $E$_P3_: Zn^1+^PC + $e$^−^ $\to$ Zn^0^PC | **(S10)** |
| *E*_P3_: O_2_ + Zn^0^PC $\leftrightarrow$ [O_2_−Zn^0^]PC | **(S11)** |
| *E*_P3_: [O_2_−Zn^0^]PC $\leftrightarrow$ [O_2_^•−^−Zn^1+^]PC $\to$ Zn^1+^PC + ^1^O_2_ $\to$ $h\upsilon$_ECL_ ($\lambda$ ≈ 641, 703 nm) | **(S12)** |

Since Eq. S4 and S5 engage only the oxygen reduction reactions (ORR), they are not getting involved in the ECL cycle of Figure 2f and the actual emission arises from the excited state of ZnPC (ZnPC*).

As schematized in Figure S8, the square-wave voltammetry (SWV) was selected to separate the contribution of core electro-reductions to ZnPC’s ECL. For a horizontal-homogenized baseline, the characteristic $I$−$V$ intervals beyond −1.35 V were not shown. From a tandem-targeted perspective, $i$_P1_ of TFPP in N_2_ persisted in air (Figure S8a, curves a and b), signifying a superposition of ORR and selective $e$^−^-injection into *N*-dominated LUMO ^[16]^ (lowest unoccupied molecular orbital). Regarding PC, it resolves into $E$_P2_ = −0.63 V and $E$_P1_ = −0.52 V (Figure S8b). Undeniably, the π-cloud expansion plus lone-pair accumulation induce this overpotential reduction. Further features appear in curves c and d, where (i) parallelized catalysis occurs at zincous $E$_P1_ in presence of O_2_ with reversibility augmenting the amplitude of $i$_P1_; and (ii) $i$_P2/3_ of ZnTFPP/ZnPC plunge 4-times more sharply than TFPP/PC, suggesting a Zn^2+^-complexed reductive cascade.^[17]^ Though discriminating the true electronic recipient from TFPP chelation is challenging;^[17]^ by subtracting $i$_P_ in air from that in N_2_ (Δ$i$_P_), the incremental trend emerges: Δ$i$_P1_ > Δ$i$_P2_ ≥ Δ$i$_P3_, indicating that unlike $E$_P1_, $E$_P2/3_ partially proceeds via pre-reduced $e$^−^-donation on the axially ligated intermediate: Zn^1+/0^−O_2_.^[17]^ Despite equal TFPP content, Δ$i$_P2_[ZnTFPP] ≈ Δ$i$_P3_[ZnTFPP] ≈ 1/2·Δ$i$_P2_[ZnPC] can be approximately appraised. A more meticulous metric is the Coulombic integral, Δ$Q$_P_ = $\int$Δ$i$_P_·d$E$_P1_, which more explicitly expresses the electrocatalytic turnover of O_2_. On this foundation, assuming Δ$i$_P3_[ZnPC] → 0, O_2_ conversion could advance to earlier completion via a low-energy barrier at $E$_P2_, highlighting an enhanced utility rate of ZnPC.

In essence, the ECL originates from a tri-state superposition: anti-bonding π in reduced TFPP^−^, *d*-orbital in Zn^2+^, and *p* in singlet O_2_^•−^.^[17]^ Evidently, chromogenic depolymerization amplifies ZnPC’s ECL. Here, contrasting Δ$G$-favored isolation in*β*-cyclodextrin host or reconstituted enzyme pocket,^[16,17]^ an entropy-favored dynamic covalent chemistry pathway was realized. Its reticulated enclosure imparted ECL additional momentum, ensuring shape-persistent permeation for coreactants due to excellent extrinsic porosity (Brunauer-Emmett-Teller surface area: ~1370 m^2^/g),^[19]^ naturally facilitating more effective coreacting contacts rather than the collisional quenching from porphyrinic auto-agglutination.^[18]^ Conversely, tetraphenylporphine $i$_P_ wavelets cannot render TFPP or PC uni-polarly emissive without persulfates, as previously reported.^[16]^ Additionally, the risk of aldehydic/imidoyl hydro-genation or dimerization via water-oil proton exchange near −2 V is negligible here,^[20]^ owing to moisture-resistant alkylated architecture of TbTm.

**Pearson’s Correlation Coefficient Computation**

The Pearson’s correlation coefficient (PCC) is a prevalent statistical protocol for colocalization characterization, ^[21−23]^ which quantifies the spatial colocalizing correlation between dual PL-channeled chromophores. The PCC mathematics is formulated in the following:

| $\text{PCC}=\frac{\sum_{i} \left( R_{i}-\bar{R} \right)\text{×}\left( G_{i}-\bar{G} \right)}{\sqrt{\sum_{i} \left( R_{i}-\bar{R} \right)^{\text{2}}\times\sum_{i} \left( G_{i}-\bar{G} \right)^{\text{2}}}}$ | **(S13)** |
| --- | --- |

where $R_{i}$ and $G_{i}$ refer respectively to the intensity integrals in the red and green channels at the $i$^th^ pixel, while $\bar{R}$ and $\bar{G}$ deem the mean intensities in the corresponding channels.

The PCC peruses pixelwise covariance across channels. Since the algorithm subtracts the mean intensity from the each individual pixel, PCC is sensitive to linear pixel-intensity relationship, yet irrelevant of signal strengths and baseline offset (i.e., background).^[24−26]^

**3. Supplementary Figures**


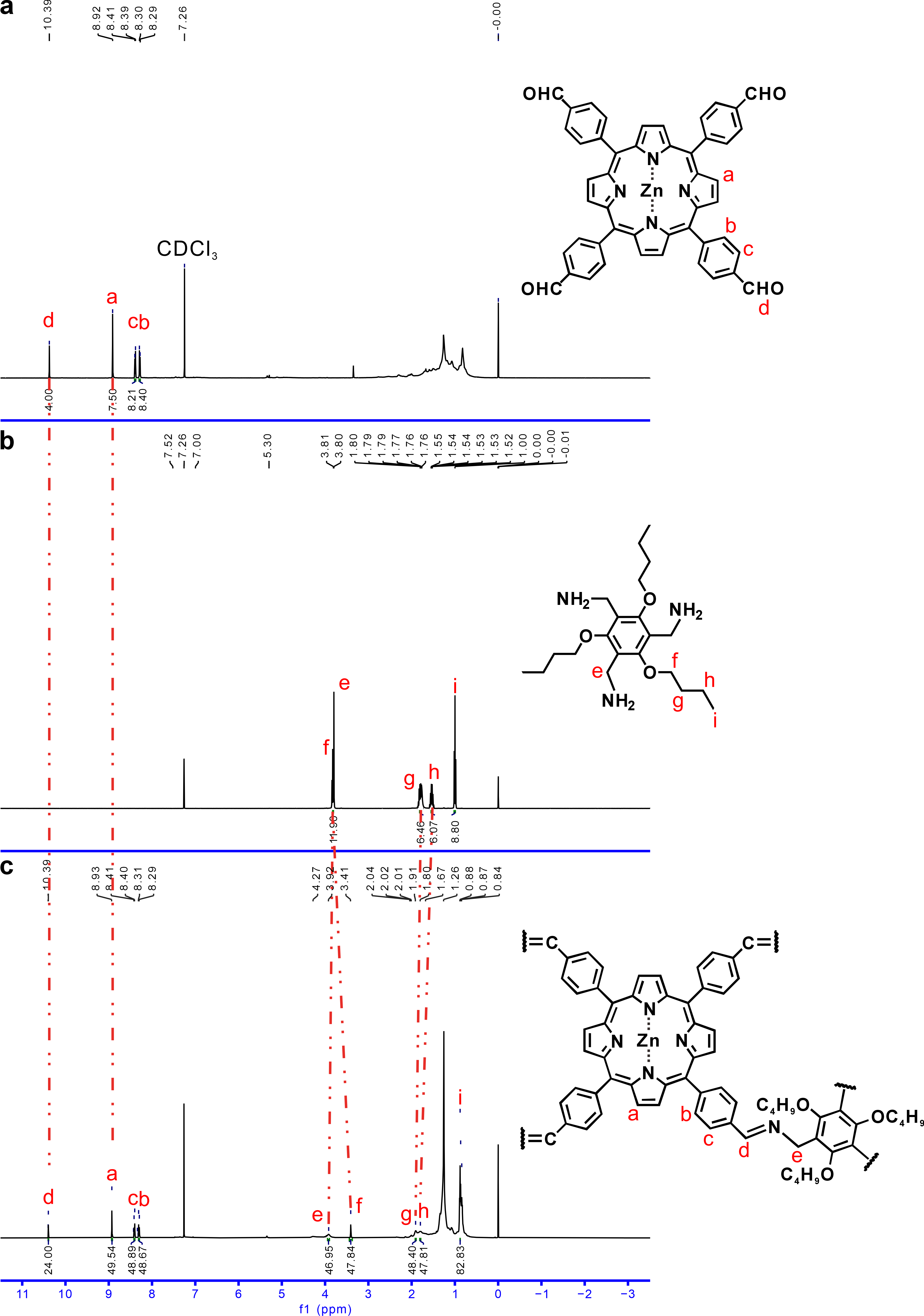


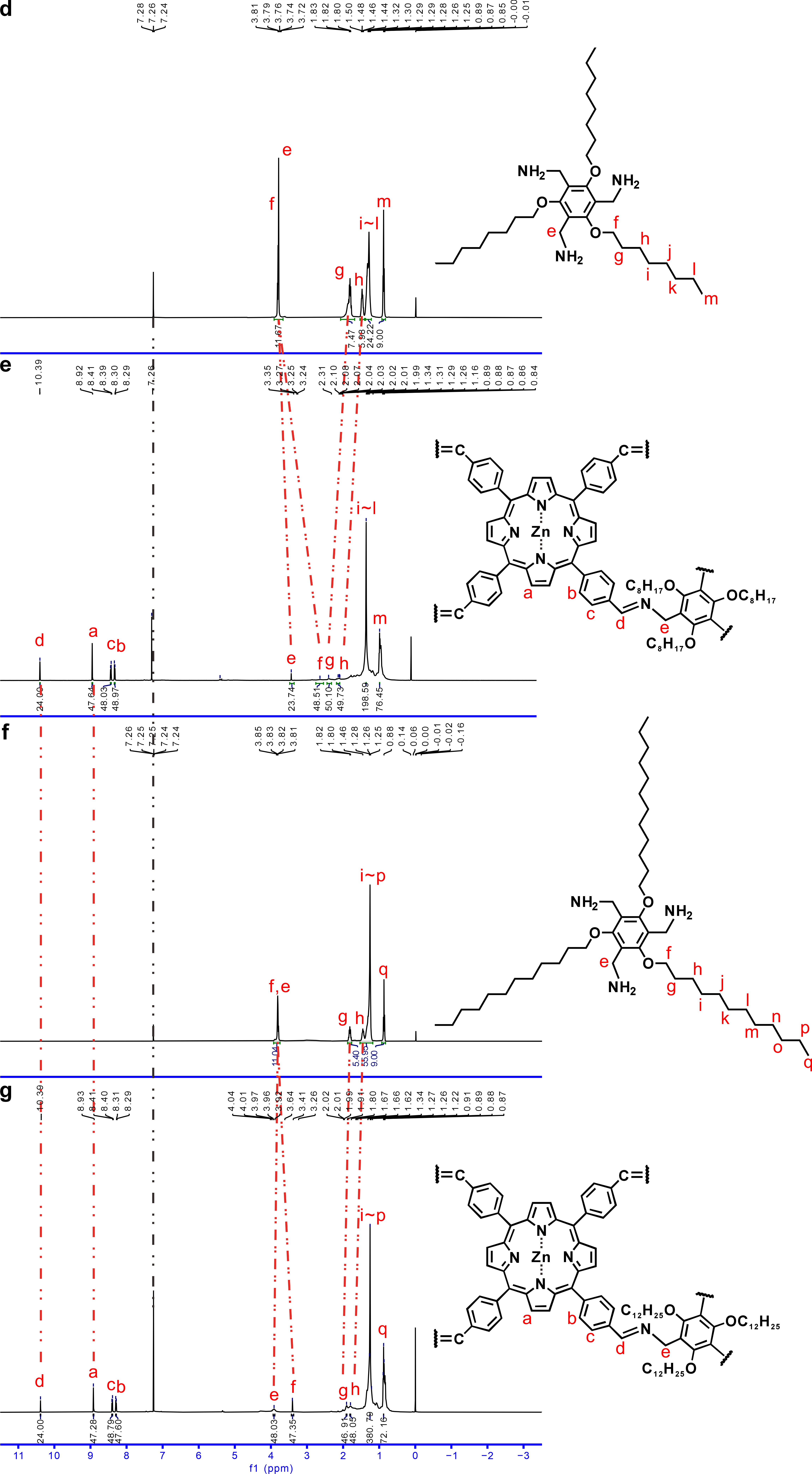


**Figure S4.** Consolidated ^1^H-NMR spectral series of **a)** ZnTFPP, **b)** ^4^TbTm, **c)** Zn^4^PC, **d)** ^8^TbTm, **e)** Zn^8^PC, **f)** ^12^TbTm, and **g)** Zn^12^PC. Each embeds its corresponding ChemDraw structure with the assignments of alphabet-coded Hs. Vertical dash-dotted lines delineate diagnostic dependencies (splitting or shifting) among characteristic chemical shifts ($\delta$):

**a)** 10.39 ($s$, 4H), 8.92 ($s$, 8H), 8.40 ($d$, $J$ = 7.9 Hz, 8H), 8.29 ($d$, $J$ = 8.0 Hz, 8H), −2.77 ($s$, 2H);

**b)** 3.80~3.81 ($m$, 12H), 1.76~1.80 ($m$, 6H), 1.52~1.55 ($m$, 6H), 1.01 ($t$, $J$ = 9.7 Hz, 9H);

**c)** 8.93 ($s$, 49H), 8.29~8.41 ($m$, 96H), 3.91~3.93 ($m$, 48H), 3.41~3.43 ($m$, 48H), 1.91~2.04 ($m$, 48H), 1.67~1.80 ($m$, 48H), 0.84~0.88 ($m$, 82H);

**d)** 3.72~3.81 ($m$, 12H), 1.80~1.83 ($m$, 6H), 1.46~1.50 ($m$, 6H), 1.25~1.44 ($m$, 24H), 0.87 ($t$, $J$ = 8.7 Hz, 9H);

**e)** 8.92 ($s$, 48H), 8.29~8.41 ($m$, 96H), 3.24~3.35 ($m$, 48H), 2.53~2.55 ($m$, 48H), 2.20~2.31 ($m$, 48H), 2.07~2.11 ($m$, 48H), 1.16~1.34 ($m$, 198H), 0.84~0.89 ($m$, 76H);

**f)** 3.81~3.85 ($m$, 12H), 1.80~1.82 ($m$, 6H), 1.25~1.46 ($m$, 56H), 0.87 ($t$, $J$ = 9.0 Hz, 9H);

**g)** 8.93 ($s$, 48H), 8.29~8.41 ($m$, 96H), 3.92~4.04 ($m$, 48H), 3.26~3.41 ($m$, 48H),1.66~1.91 ($m$, 96H), 1.22~1.34 ($m$, 380H), 0.87~0.91 ($m$, 72H).


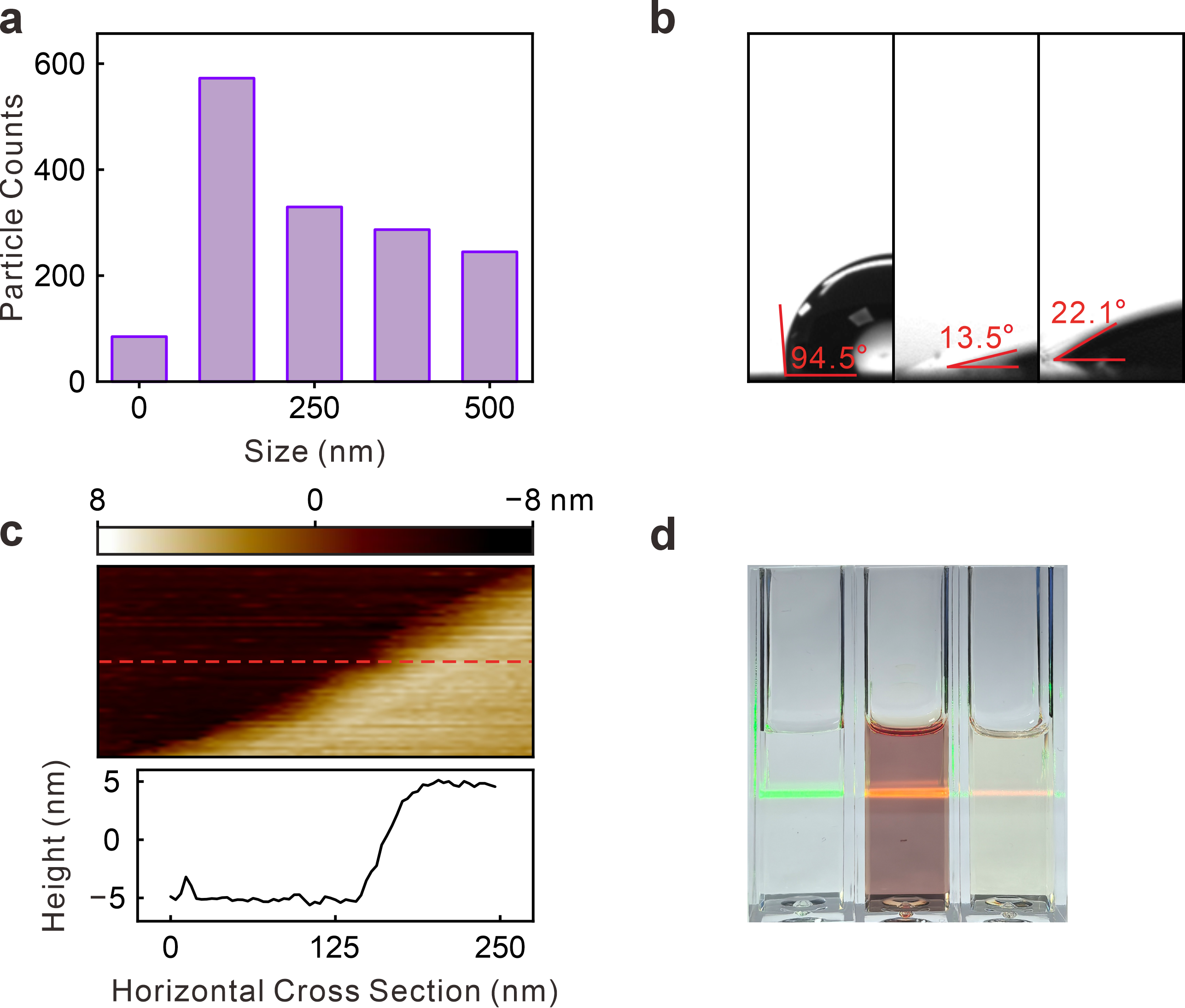


**Figure S5.** **a)** Statistical sizing survey of raw ZnPCs corresponding to Figure 2a, extracted using ImageJ. **b)** Static water contact-angle ($\theta$_c_) goniometric gauging at the triphasic junction (left: ZnPC; middle: POPC/Chl; right: ZnPC@POPC/Chl). **c)** Cross-sectional collection from AFM topographs (upper) and Corresponding three-dimensional (3D) surface reconstruction highlighting the uniform film morphology (lower). The surface roughness ($R$_q_) was reckoned to be 0.47±0.06 nm based on the tests of three independently prepared samples. **d)** Tyndall-type testimony of colloidal commixtures: lipo[POPC/Chl] (left), ZnPCs (middle), and lipo[ZnPC@POPC/Chl] (right) in CHCl_3_. Incident laser light: 532 nm, offered from Deli Group Co., Ltd.


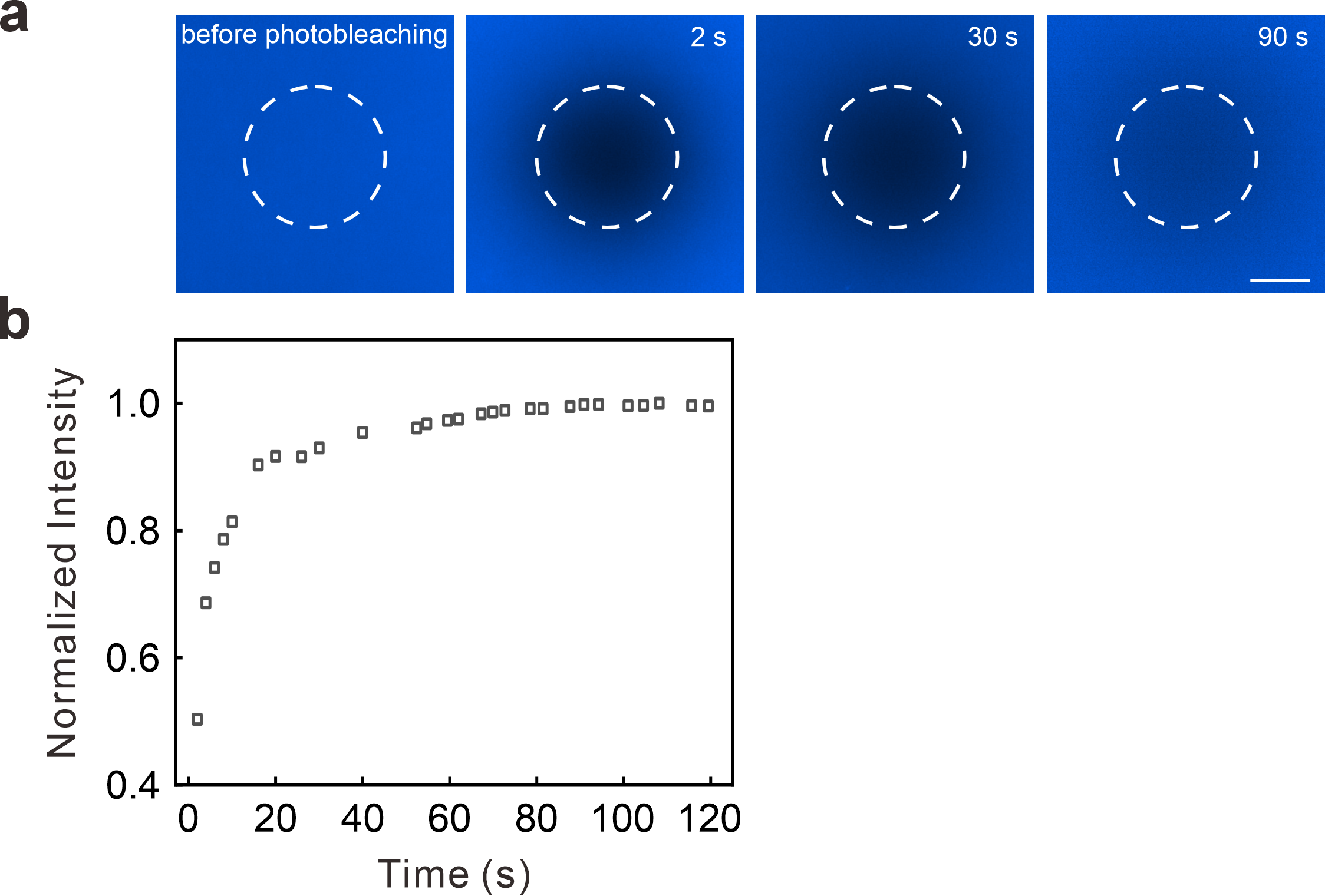


**Figure S6.** **a)** Representative fluorescence figures of ZnPC@POPC/Chl with l mol.% NBD-PS on a piece of coverslip before photobleaching and at various recovery moments after photobleaching (2, 30 and 90 s). The white dashed loops represent the photobleached region. **b)** Time-tracked fluorescence recovery in the photobleached point in **a)**, plotted as normalized intensity versus time, telling the lateral lipid mobility. Scale bar: 5 μm.


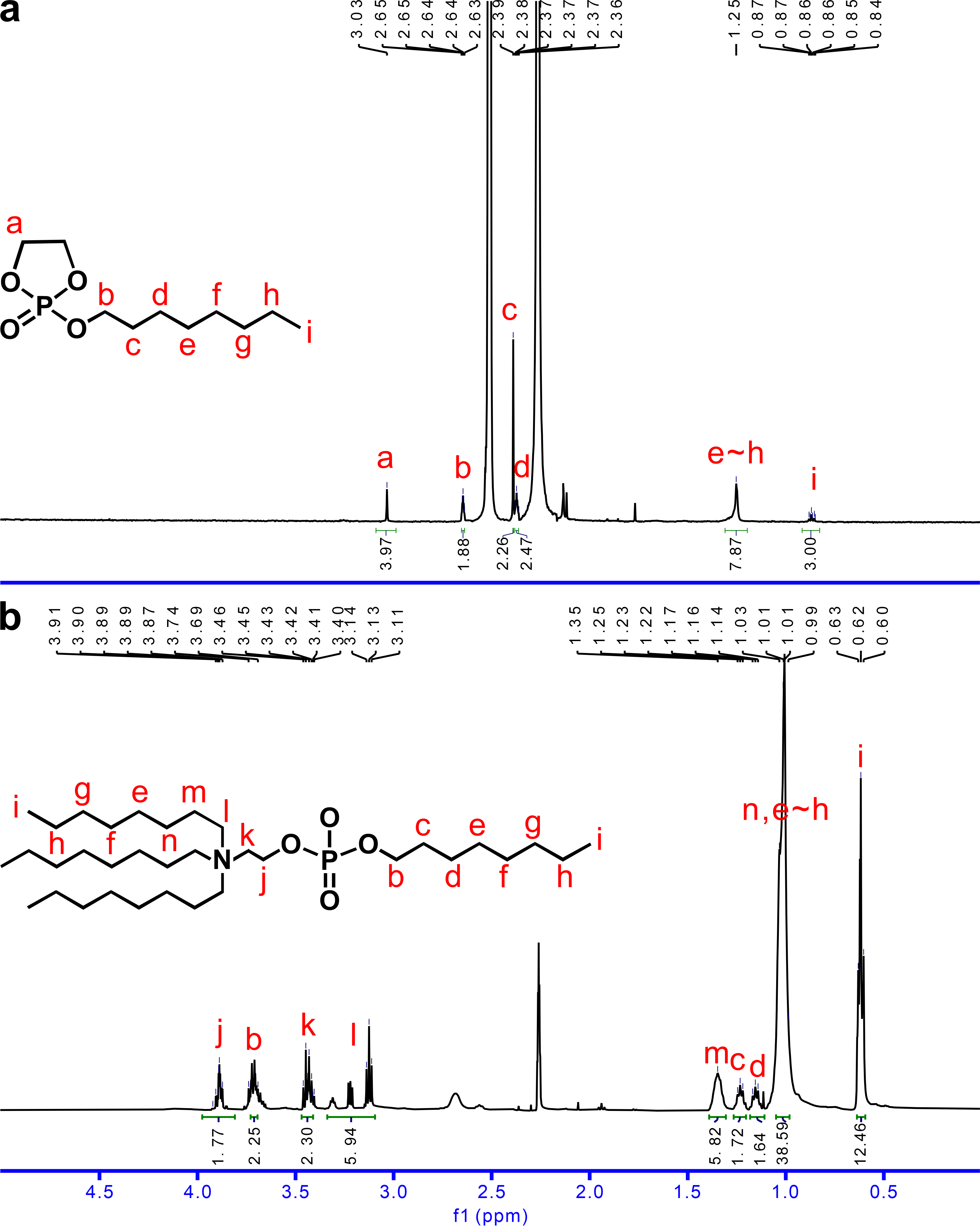


**Figure S7.** Merged ^1^H-NMR spectral maps of **a)** CODP and **b)** TOAP, each embedding a corresponding ChemDraw depiction that correlates characteristic Hs in alphabets. Chemical shifts ($\delta$): **a)** 3.03 ($s$, 4H), 2.63~2.65 ($m$, 2H), 2.39 ($s$, 2H), 2.36~2.37 ($m$, 2H), 1.25 ($s$, 8H), 0.84~0.87 ($m$, 3H); and **b)** 3.89~3.91 ($m$, 2H), 3.69~3.74 ($m$, 2H), 3.42~3.46 ($m$, 2H), 3.13~3.31 ($m$, 4H), 1.35 ($s$, 4H), 1.21~1.24 ($t$, 2H), 1.14~1.16 ($m$, 2H), 0.98~1.03 ($m$, 38H), 0.60~0.63 ($m$, 12H).


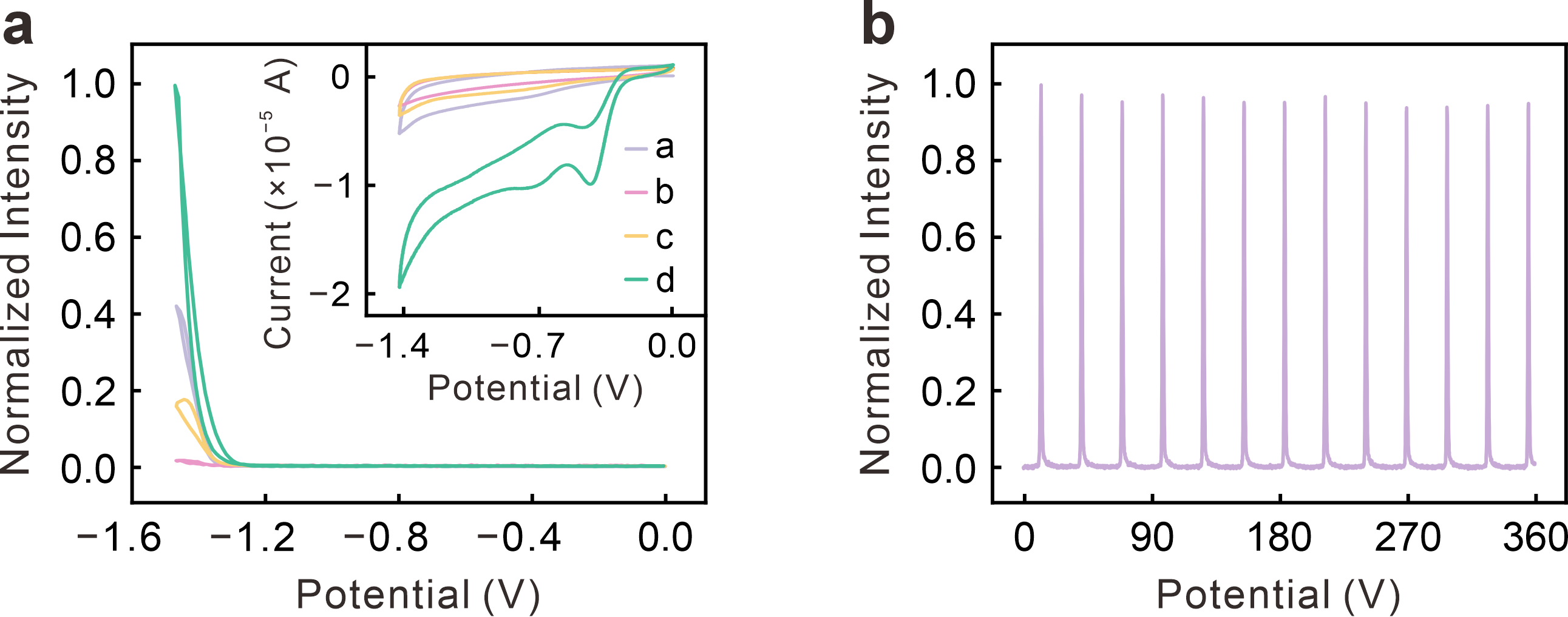


**Figure S8.** **a)** ECL-potential profiles recorded under air (a), N_2_ (b), N_2_ supplemented with 400 μM H_2_O_2_ (c), and O_2_ (d) atmospheres. **Inset:** synchronized cyclic voltammograms, scan rate: 100 mV/s, scan range: [0, −1.45] V. **b)** Stability surveillance of signal persistence over time.


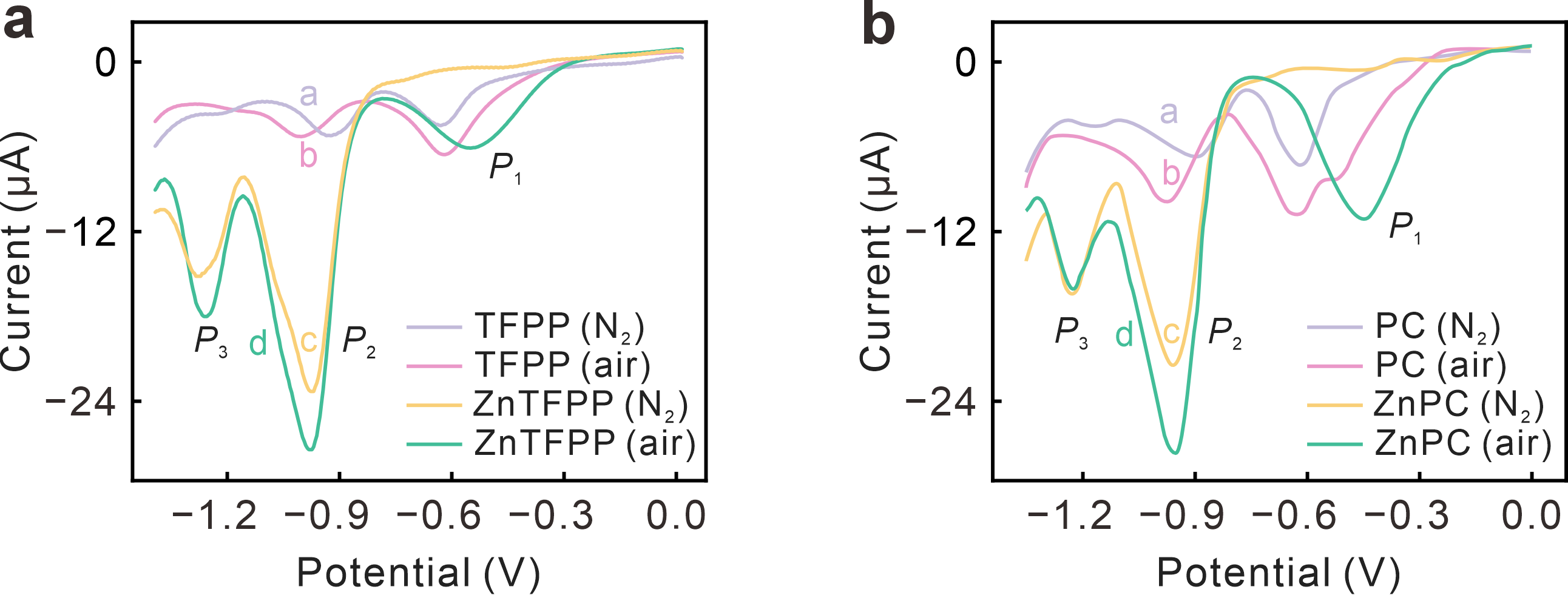


**Figure S9.** **a)** Square-wave voltametric signatures of TFPP (a, b), ZnTFPP (c, d) under N_2_ (a, c) and air (b, d). **b)** Corresponding square-wave voltametric signatures of PC (a, b), and ZnPC (c, d) under identical atmospheres.


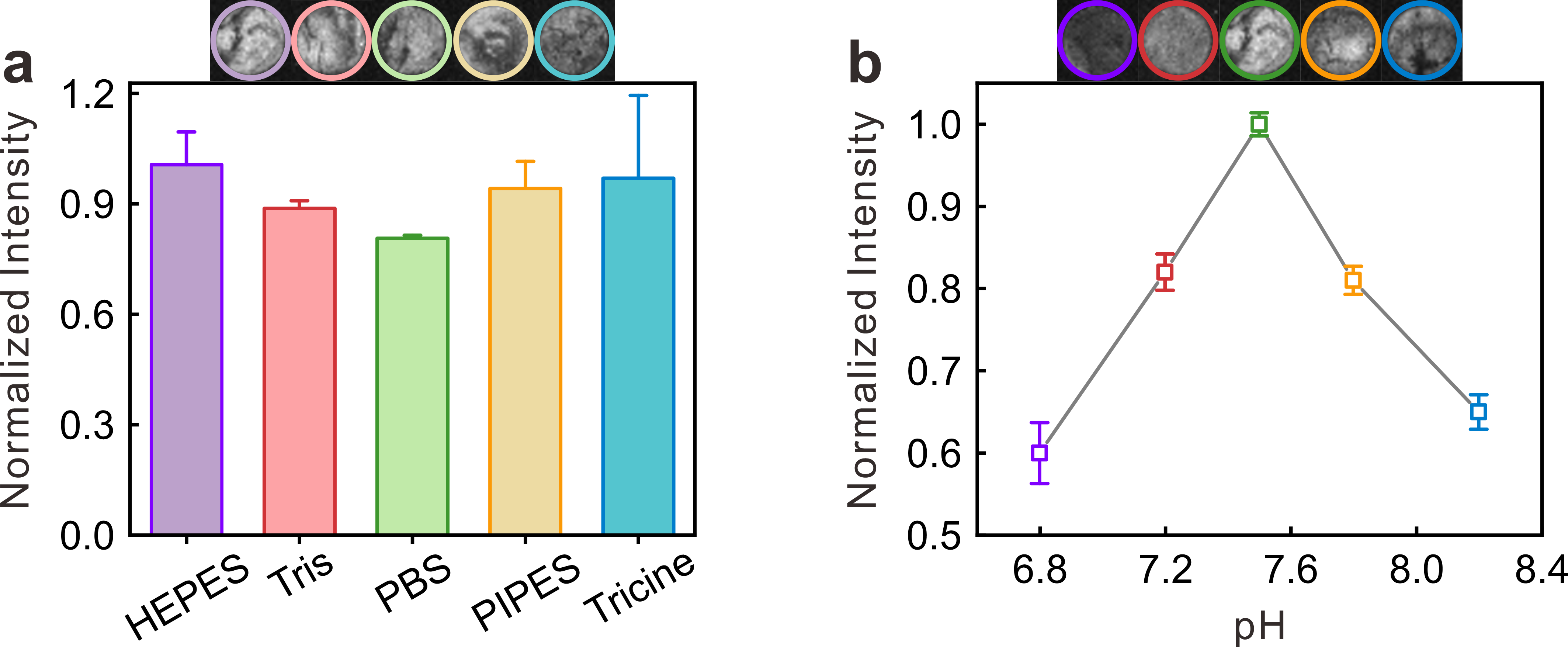


**Figure S10.** Systematic screening of detection settings in terms of normalized ECL intensity ($I$_ECL_): **a)** solution systems (HEPES: 4-(2-hydroxyethyl)piperazine-1-ethanesulfonic acid, Tris: tri(hydroxymethyl)amino-methane, PBS: phosphate buffered saline, PIPES: piperazine-1,4-bis(ethanesulfonic acid), Tricine: *N*-tris (hydroxymethyl)methylglycine). All buffer solutions were 10 mM at pH 7.5 for initial screening; and **b)** solution pH of HEPES (10 mM). Upper panels display ECL exposures at GCE/ZnPC@POPC/Chl under corresponding circumstances.


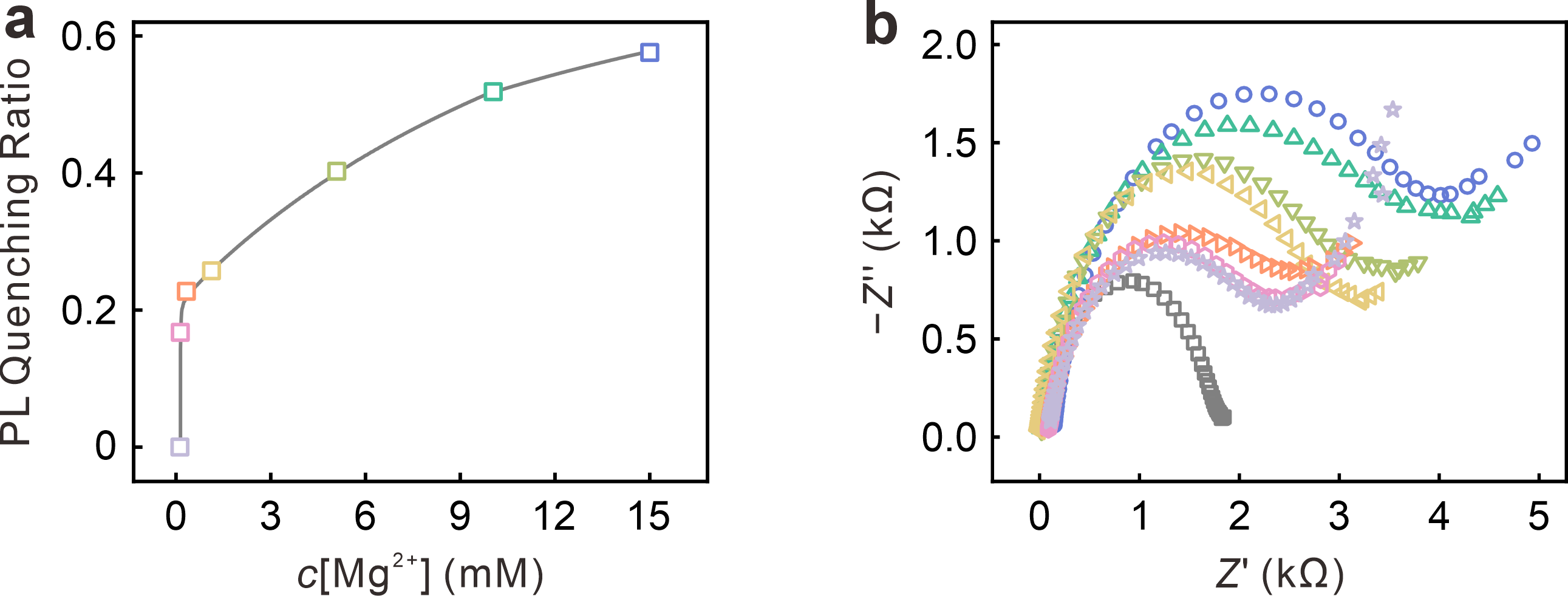


**Figure S11.** Langmuir-type isothermal fitting correlating normalized Δ$I$_PL_ (1−$I$_PL_/$I$_PL_^Θ^) with $c$[Mg^2+^] by adding Mg^2+^-selective Mag-Fluo-4 ($\lambda$_ex_/$\lambda$_em_ = 494/516 nm, see the 3^rd^ paragraph of "Electrode Surface Functionalization and Membrane Interaction Investigations" in SI) into the solution before and after each ECL check. Above is the album of ECL exposures at corresponding concentration indicated by the color of circle. **Inset:** the corresponding Nyquist plots at GCE/ZnPC@POPC/Chl in 0.3 M KCl containing 10 mM pH 7.4 HEPES and 5 mM Fe(CN)_6_^3−/4−^ at an open-circuit potential of −0.2 V and frequencies from 10^−2^ to 10^5^ Hz. Every electron-transfer resistance ($R$_ET_) in semicircle was collected for fitting.


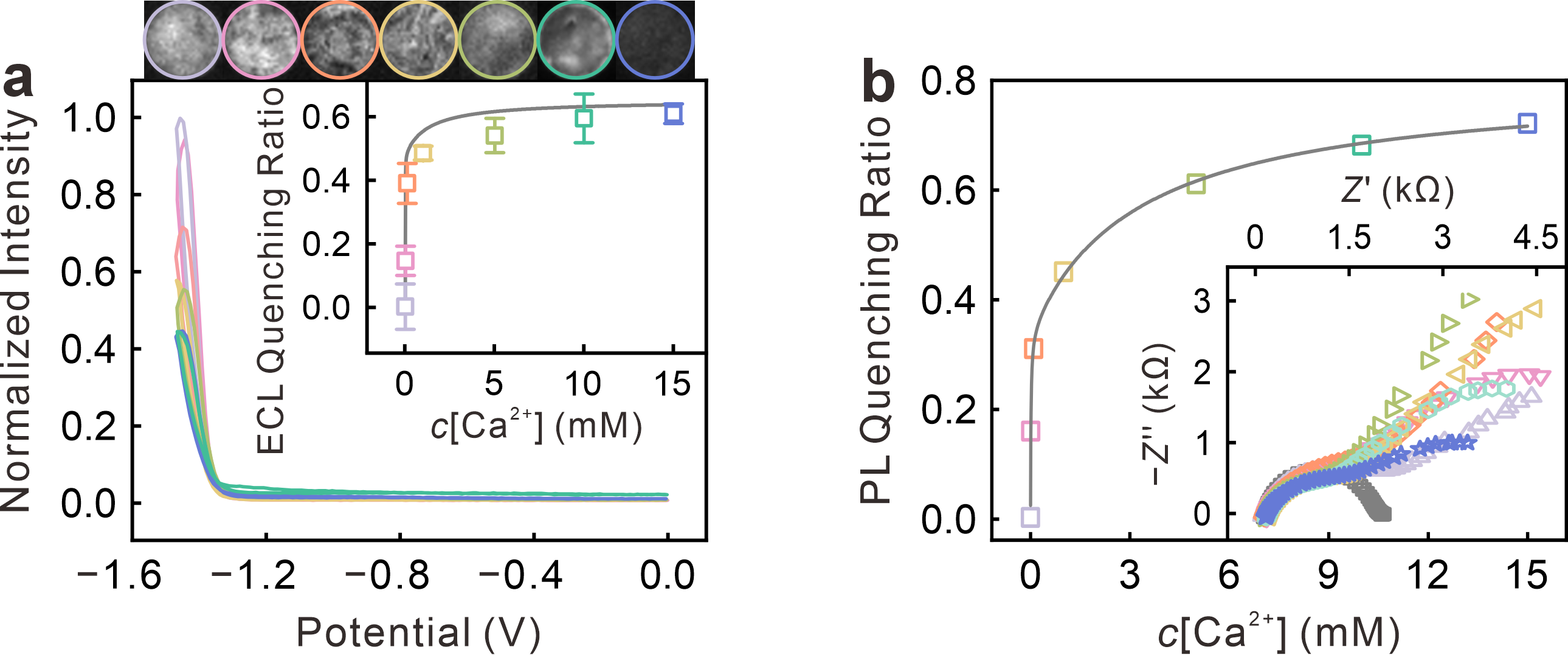


**Figure S12.** **a)** ECL–potential progressions as a function of $c$[Ca^2+^] (0.01−15 mM). **Inset:** Langmuir isothermal fit between Δ$I$_ECL_ (1−$I$_ECL_/$I$_ECL_^Θ^) and $c$[Ca^2+^]. Above is the album of ECL exposures at corresponding concentration indicated by the colour of circle. **b)** Langmuir isothermal fit between the quenching ratio Δ$I$_PL_ (1−$I$_PL_/$I$_PL_^Θ^) and $c$[Ca^2+^] by adding a Ca^2+^-selective Rhodamine B hydrazide ($\lambda$_ex_/$\lambda$_em_ = 510/578, see the 3^rd^ paragraph of "Electrode Surface Functionalization and Membrane Interaction Investigations") into the solution before and after each ECL check. Above is the album of ECL exposures at corresponding concentration indicated by the color of circle. **Inset:** the corresponding EIS Nyquist plots at GCE/ZnPC@POPC/Chl in 0.3 M KCl containing 10 mM pH 7.4 HEPES and 5 mM Fe(CN)_6_^3−/4−^ at an open-circuit potential of −0.2 V and frequencies from 10^−2^ to 10^5^ Hz. Every electron-transfer resistance ($R$_ET_) in semicircle was collected for fitting.


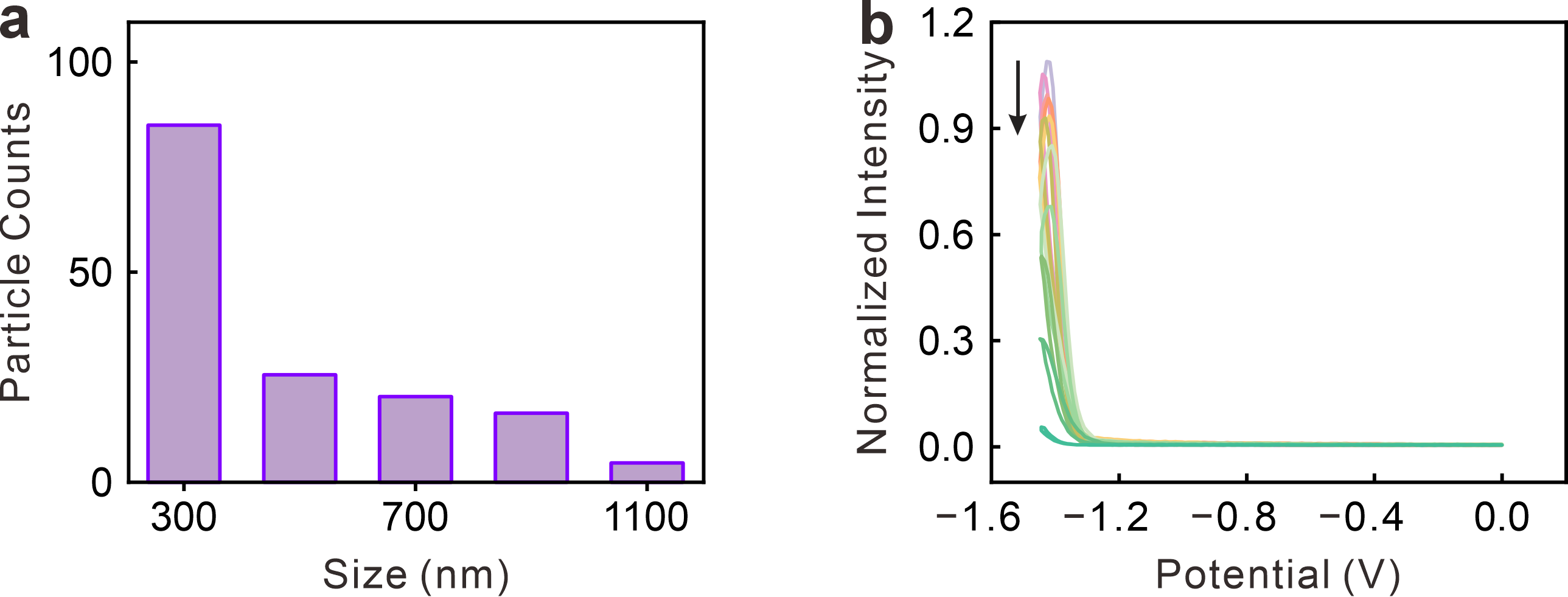


**Figure S13.** Diameter distributions of transmembrane melittin-induced perforations extracted from Figure 3e.


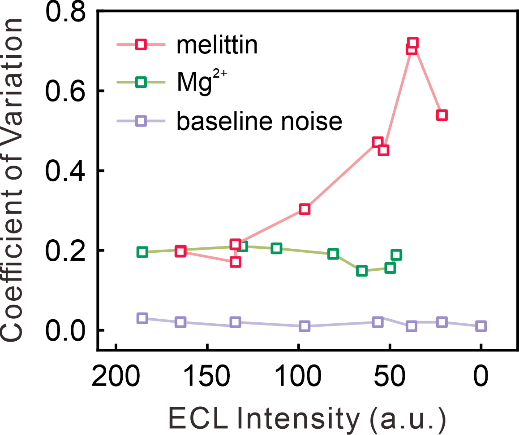


**Figure S14.** Coefficient of variation (CV) versus mean ECL intensity ($n$ = 5) during signal suppression produced by melittin (red) and Mg^2+^ (green). The baseline background trace is displayed in purple.


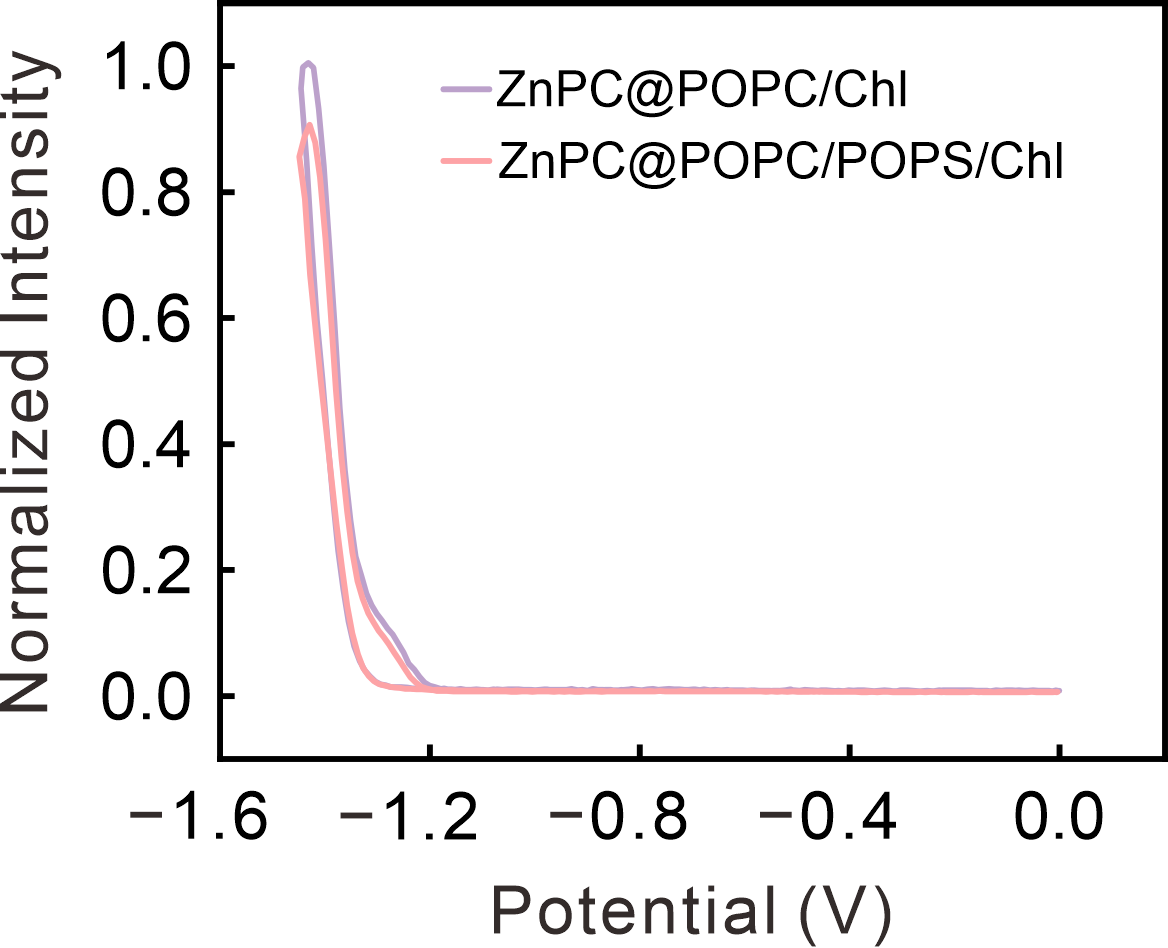


**Figure S15.** Comparative ECL-potential curves of ZnPC@POPC/Chl and ZnPC@POPC/POPS/Chl at $c$_ZnPC_: $c$_POPC_: $c$_POPS_: $c$_Chl_ = 1: 100: 10: 50.


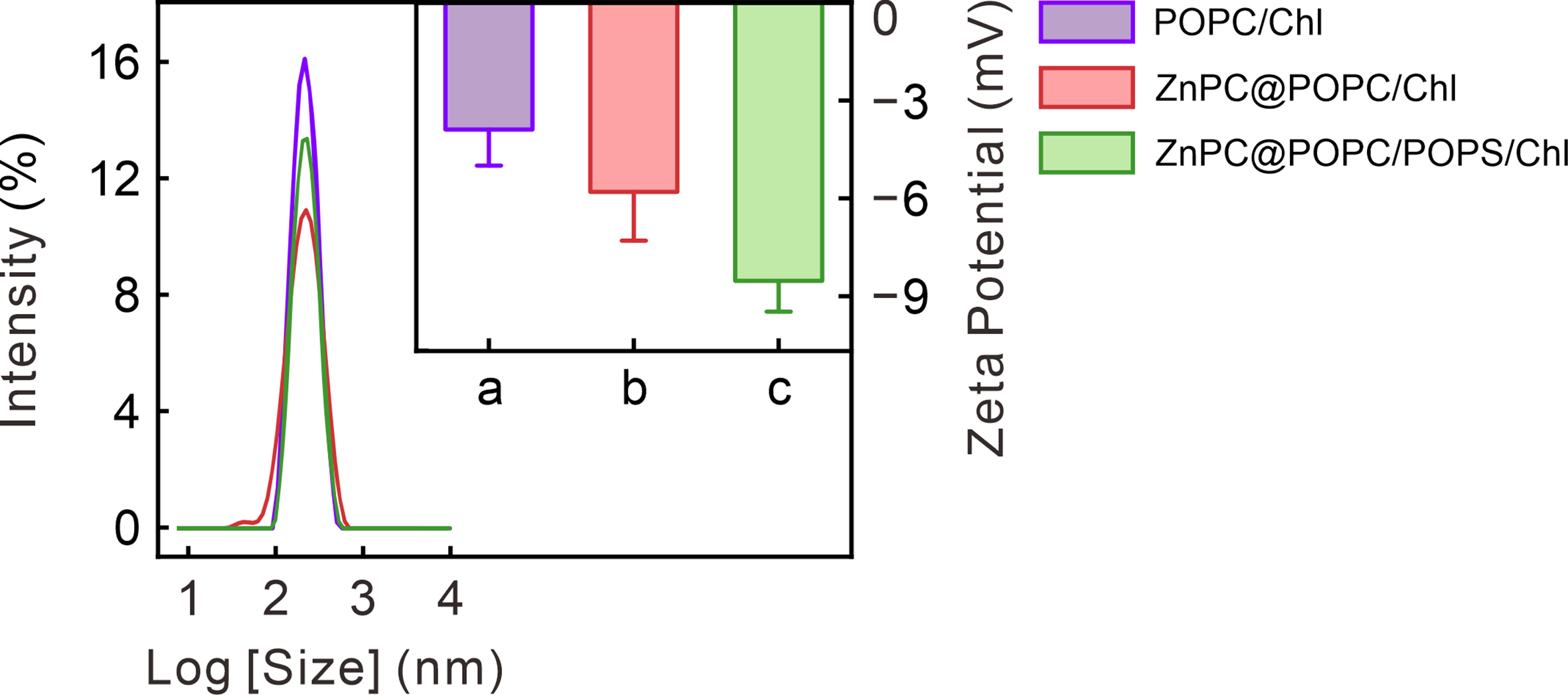


**Figure S16.** Dynamic light scattering-derived diameters of vesicles assembled from **a)** POPC/Chl ($d$ = 190 nm), **b)** ZnPC@POPC/ Chl (195 nm), and **c)** ZnPC@POPC/POPS/Chl (196 nm). **Inset:** the corresponding chart of zeta potentials ($\zeta$), exceeding electrostatic stabilization thresholds.


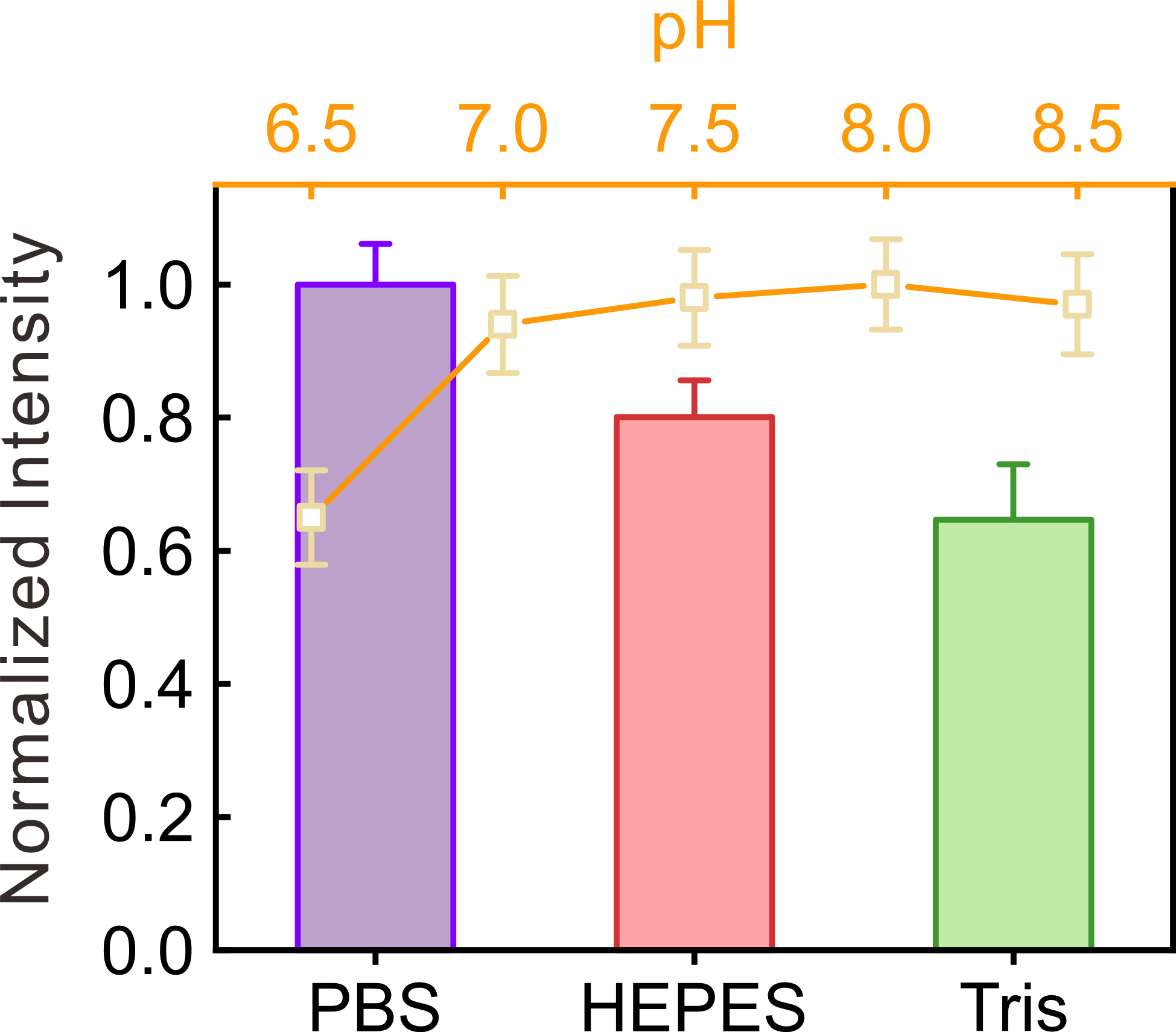


**Figure S17.** Conditional contrasts of normalized $I$_ECL_ across solution classes (histogram) and pH values (line and dots).


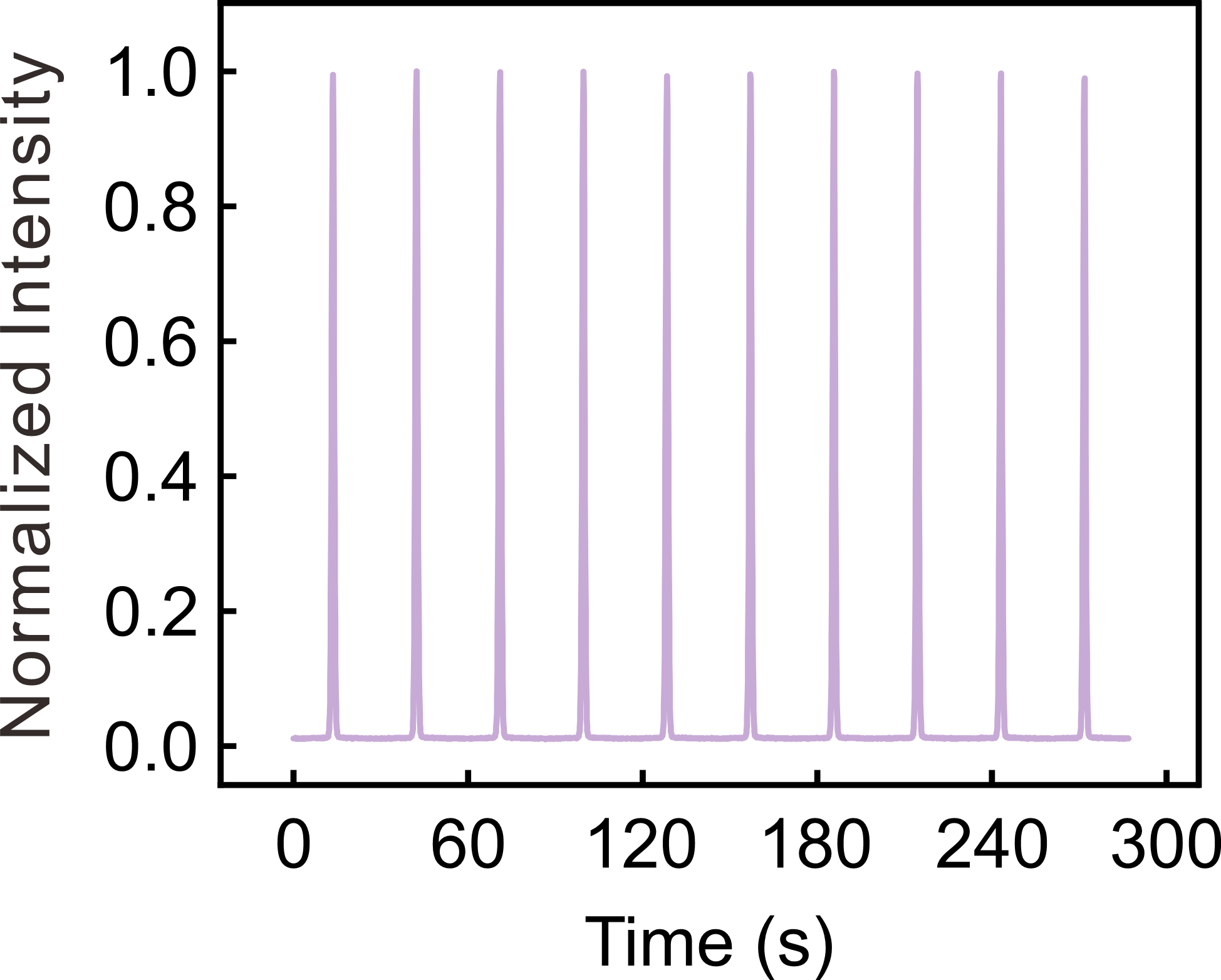


**Figure S18.** Sequential series of ECL emission collected over ten consecutive scanning loops at GCE/ZnPC@POPC/POPS/Chl.


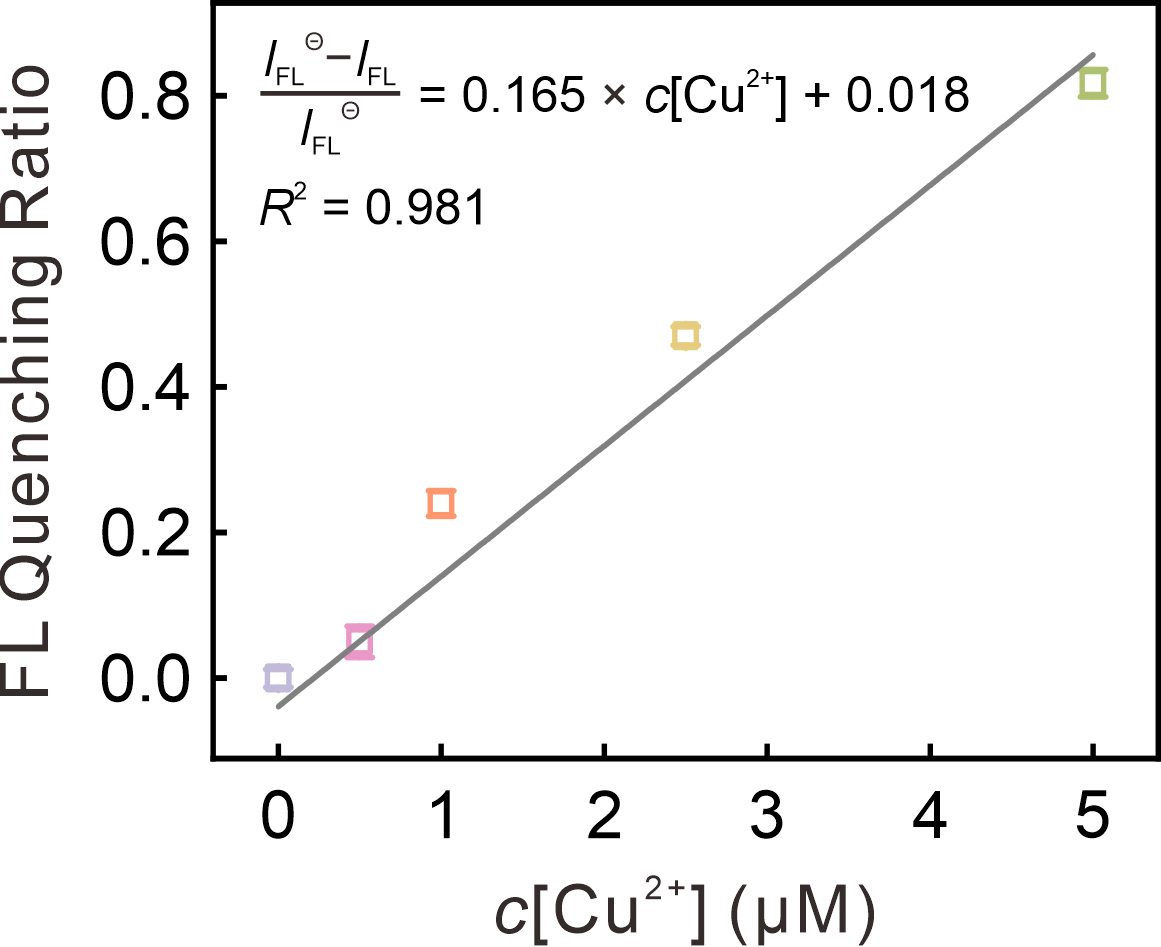


**Figure S19.** Linear fitting between normalized Δ$I$_PL_, i.e., ($I$_PL_^Θ^−$I$_PL_)/$I$_PL_^Θ^, and $c$[Cu^2+^] using a Cu ^2+^-responsive Rhodamine B hydrazide in the buffer before and after ECL emissions at GCE/ZnPC@POPC/POPS/Chol. **Inset:** fitted equation with correlation coefficient, ($I$_PL_^Θ^−$I$_PL_)/$I$_PL_^Θ^ = 0.147 × $c$[Cu^2+^] + 0.032, $R$^2^ = 0.989.


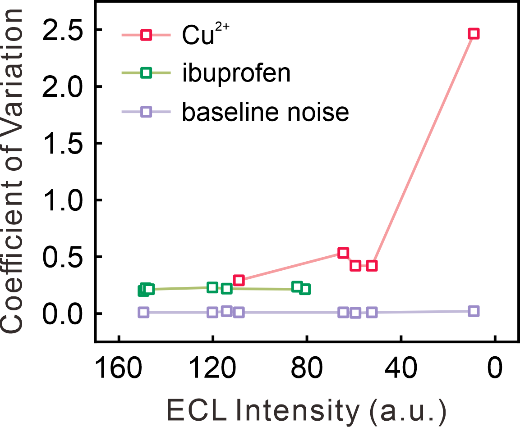


**Figure S20**. Coefficient of variation (CV) versus mean ECL intensity ($n$ = 5) during signal suppression produced by Cu^2+^ (red) and ibuprofen (green). The baseline background trace is displayed in purple.


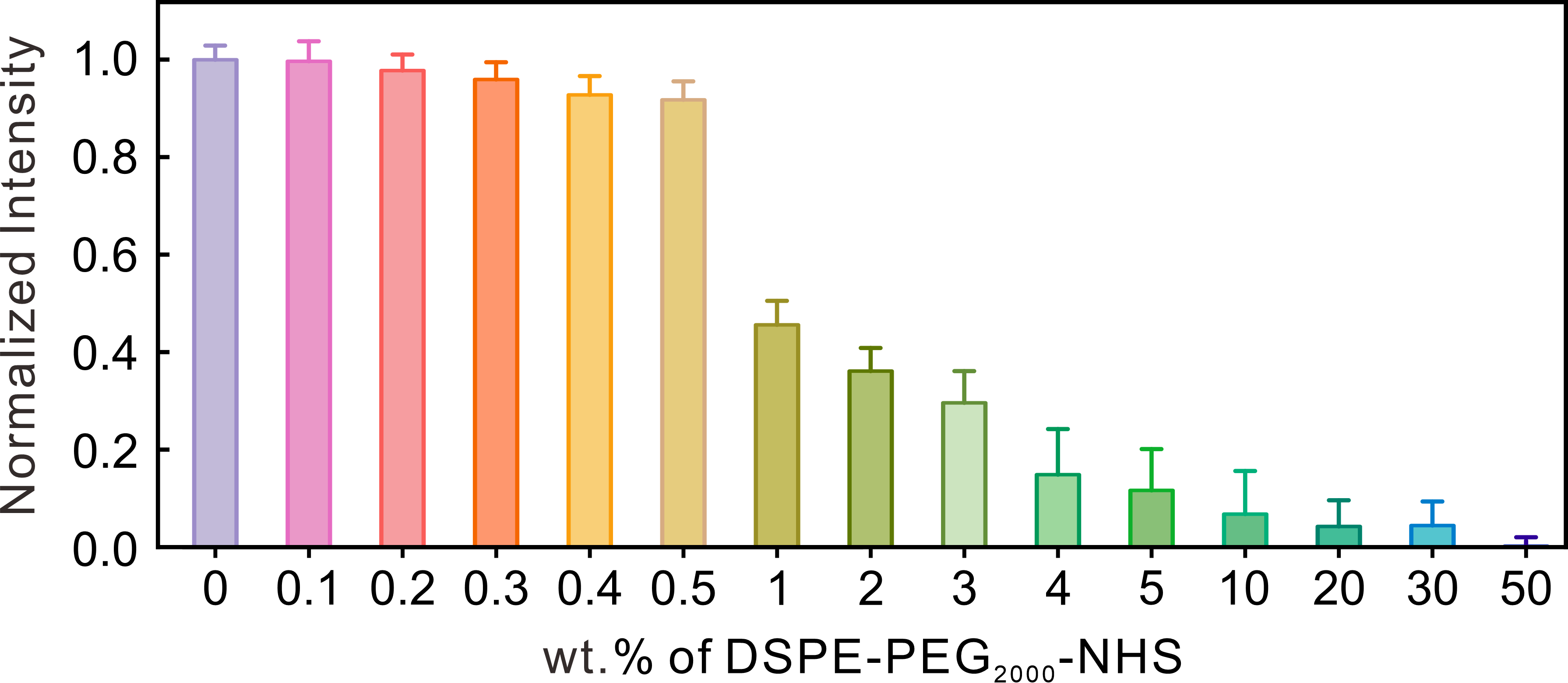


**Figure S21.** Stoichiometric scaling of DSPE-PEG-NHS doping density expressed as a mass fraction relative to POPC. The capped standard deviations were derived from three replicates ($n$ = 3).


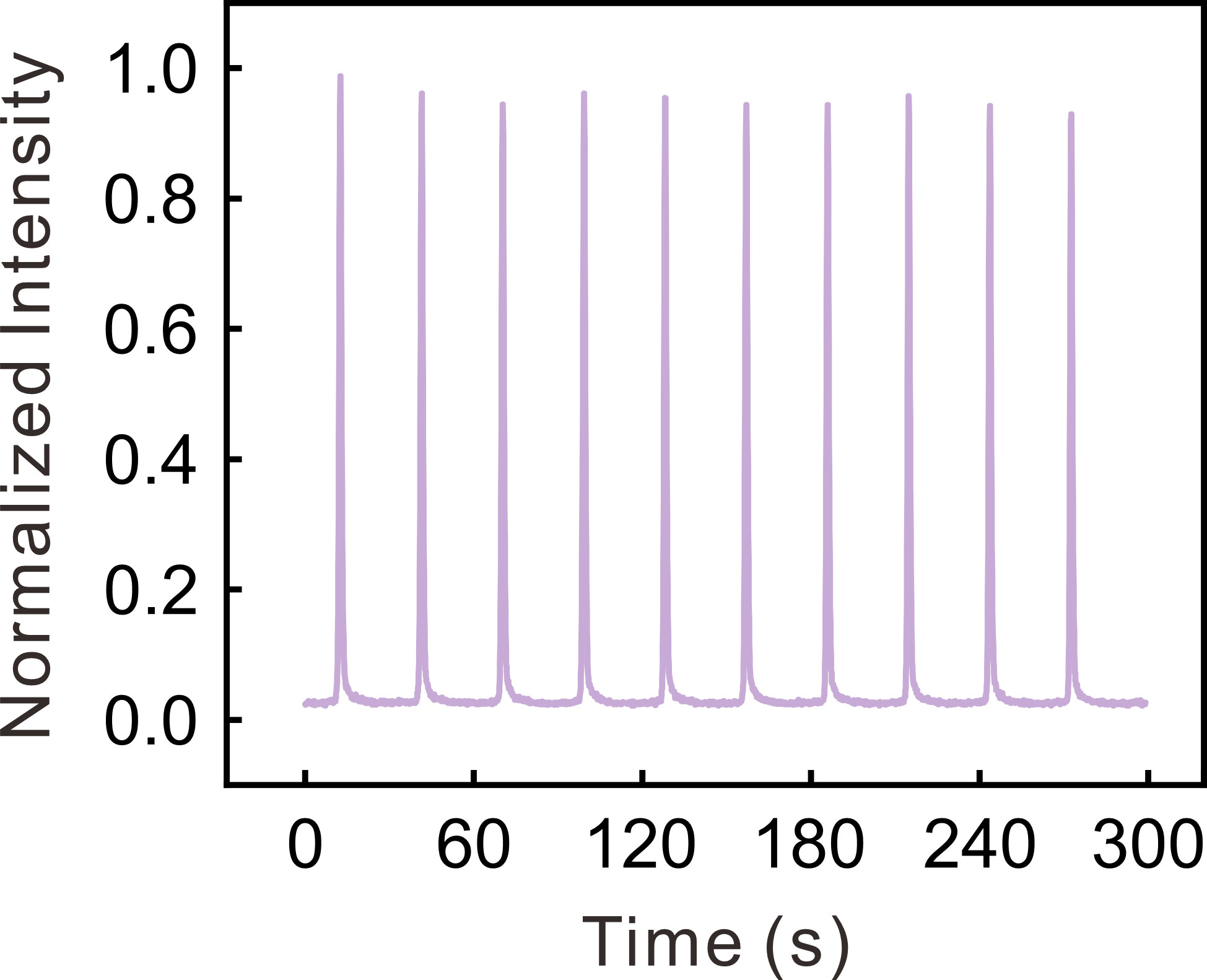


**Figure S22.** Temporal tracing of ECL emission evolution over a 300 s window at GCE/ZnPC@POPC/DSPE-PEG_2000_-NHS/Chl.


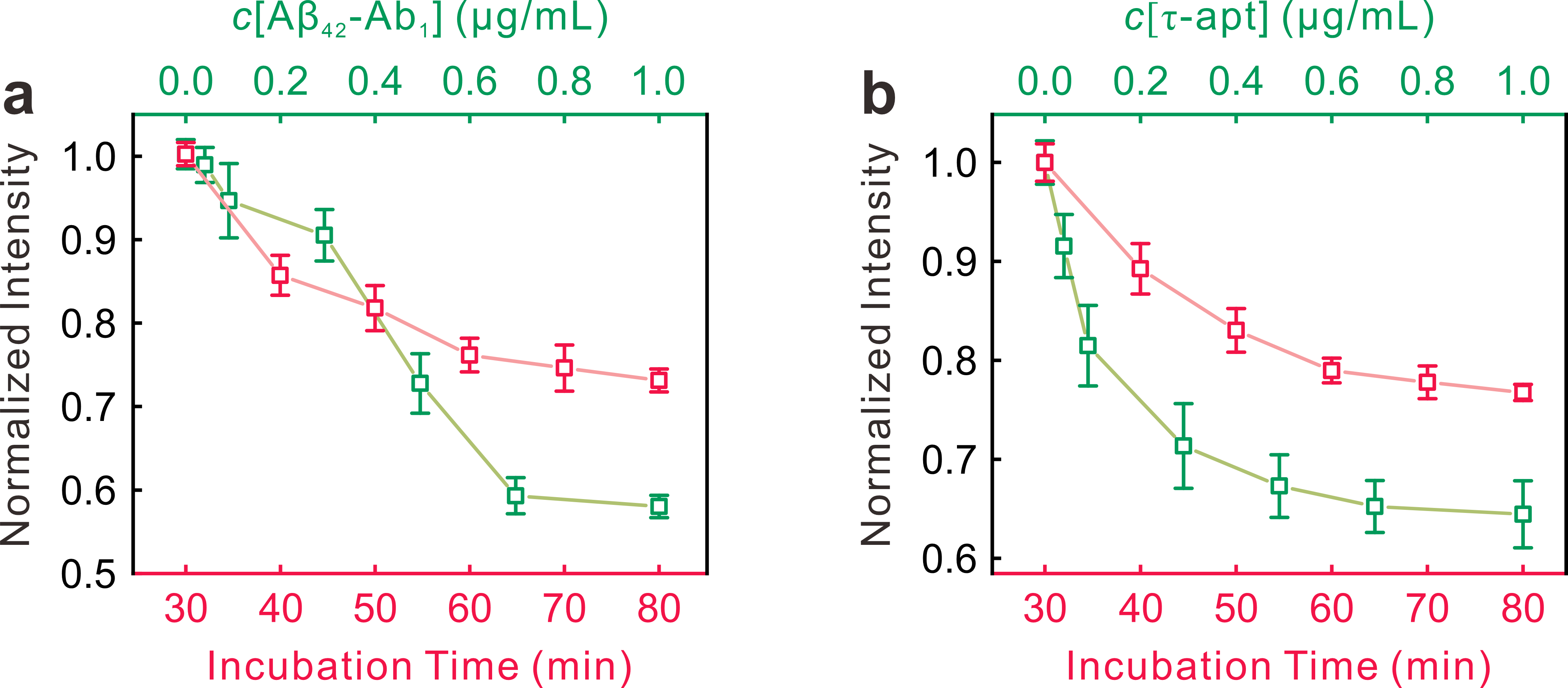


**Figure S23.** Parameter preselection for **a)** *c*[Aβ_42_-Ab_1_] (green) and incubation duration ($t$_incubation_) of Aβ_42_ (red), and **b)** *c*[τ-apt] (green) and incubation duration ($t$_incubation_) of tau protein (red).


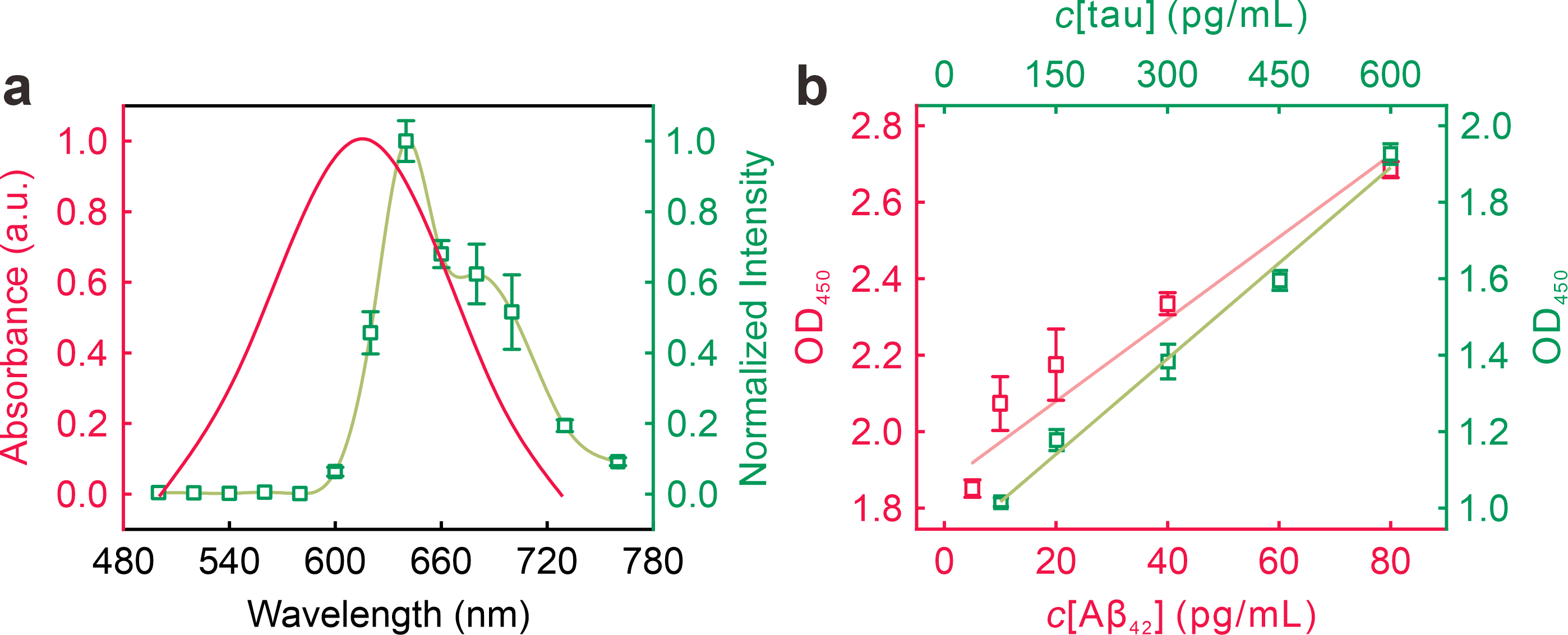


**Figure S24.** **a)** Spectral superposition between ECL emission of lipo[ZnPC@POPC/DSPE-PEG-NHS/Chl] (green) and UV absorption of AuNCs (red). **b)** Straight-line standardizations for quantifying Aβ_42_ (red) and tau (green) based on ELISA (enzyme-linked immunosorbent assay). OD_450_ = 0.00938×$c$[Aβ_42_] + 1.91080, $R$^2^ = 0.984; OD_450_ = 0.01147×$c$[Aβ_42_] + 1.32550, $R$^2^ = 0.991.


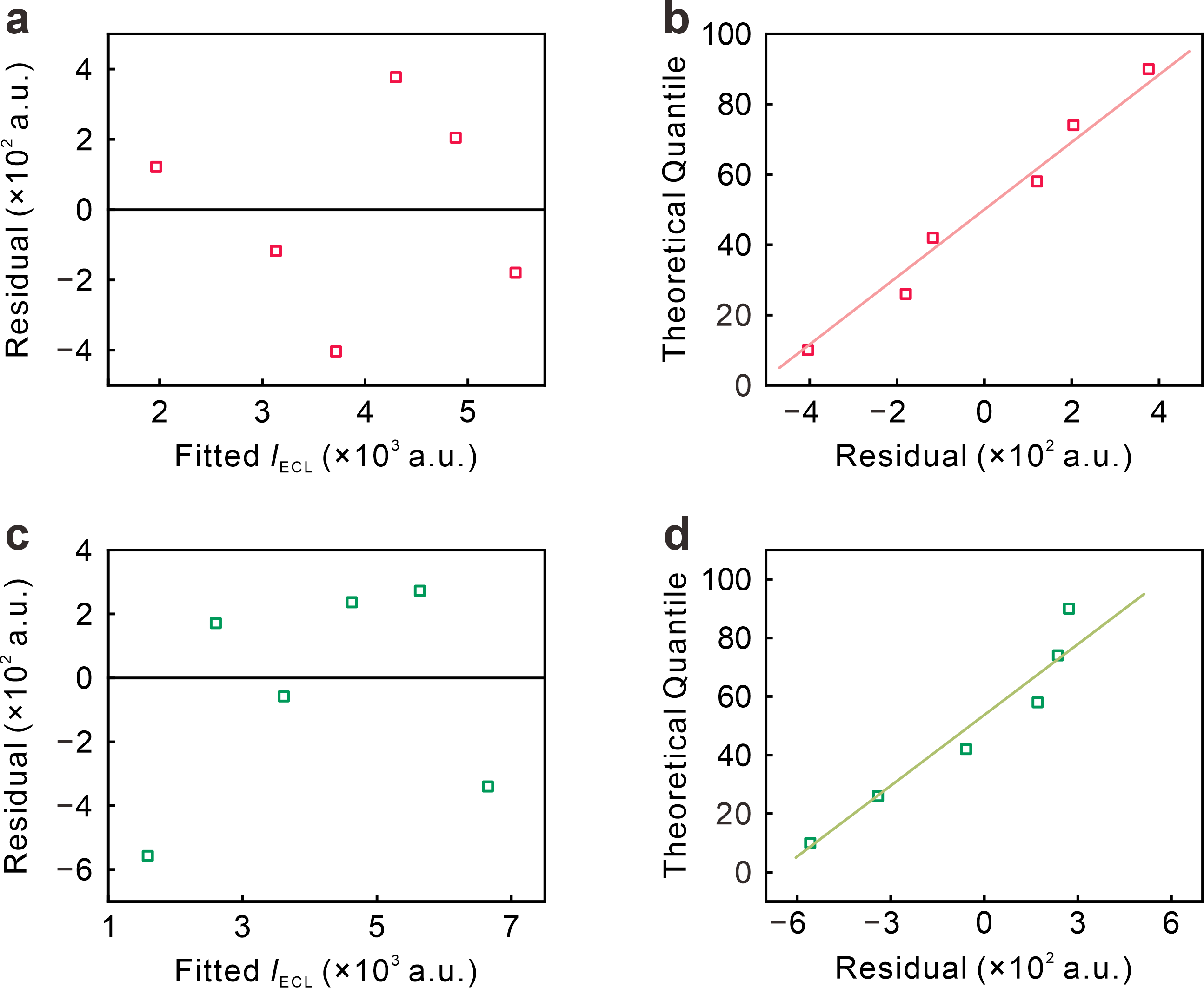


**Figure S25.** Statistical validation of the linear regression model via residual analysis. **a)** Residuals plotted against fitted $I$_ECL_ values obtained from the linear regression of $I$_ECL_ versus log($c$[Aβ_42_]). **b)** Normal probability plot of the residuals. **c)** Residuals plotted against fitted $I$_ECL_ values obtained from the linear regression of $I$_ECL_ versus log($c$[tau]). **d)** Normal probability plot of the residuals.


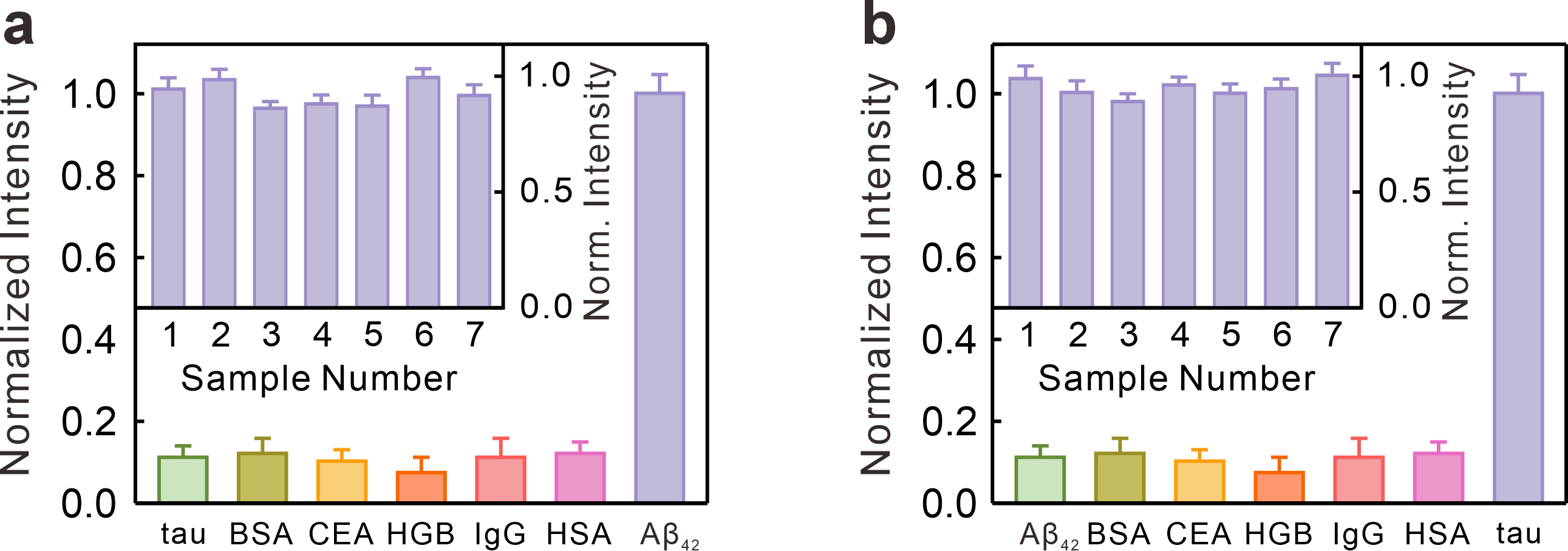


**Figure S26.** Specificity inspections against potential interferents of **a)** Aβ_42_ and **b)** tau (from left to right): BSA (bovine serum albumin), CEA (carcinoembryonic antigen), HGB (haemoglobin), lgG (immunoglobulin G), and HSA (human serum albumin).


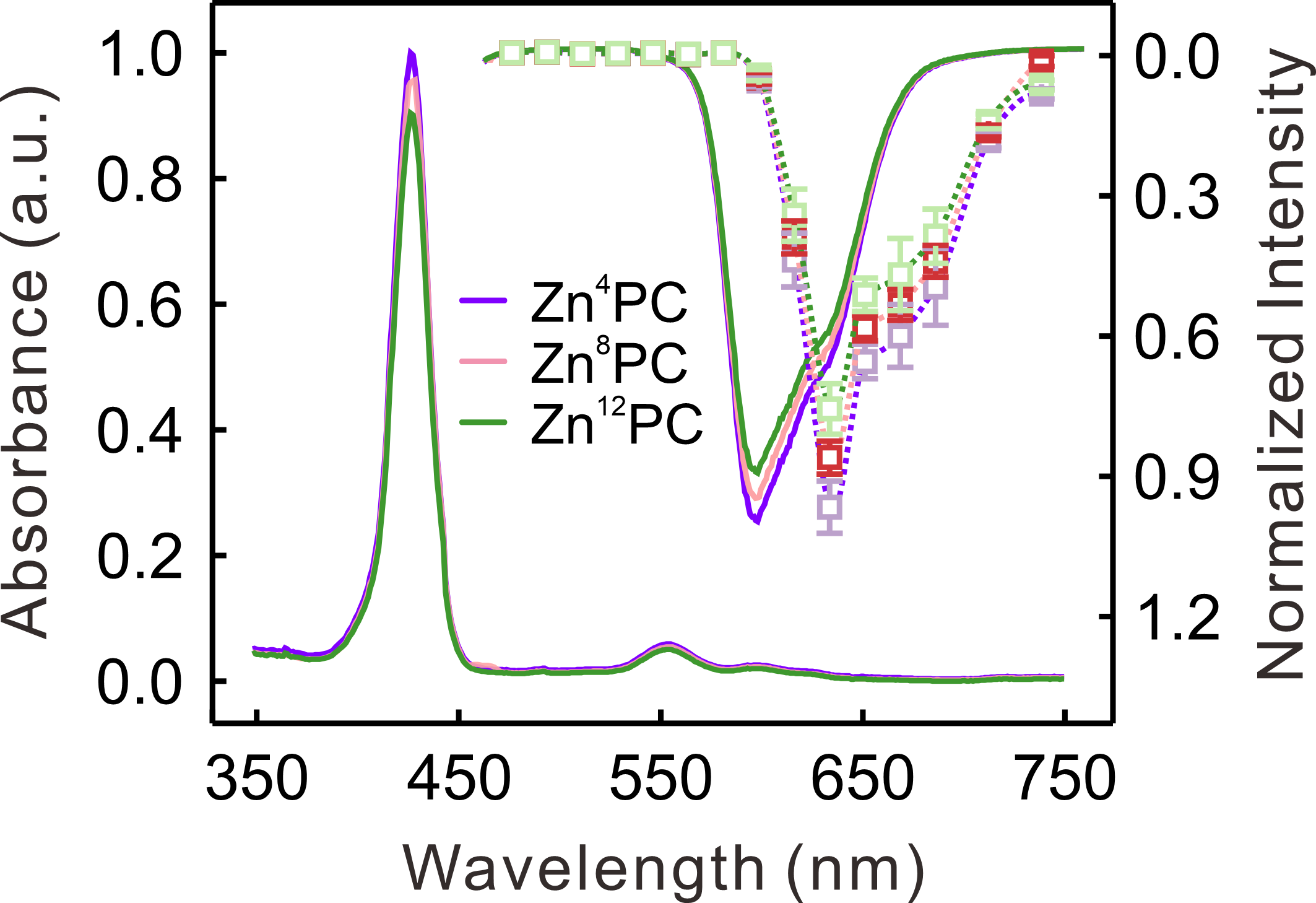


**Figure S27.** Spectroscopic synopses including UV-Vis absorbance (left $y$), PL emission (right $y$), and bandpass-filtered ECL spectra (right $y$) of equimolar Zn^4^PC, Zn^8^PC, and Zn^12^PC embedded in POPC/Chl bilayers (for ECL) or liposomes (for UV-Vis and PL) in 10 mM HEPES (pH 7.5).


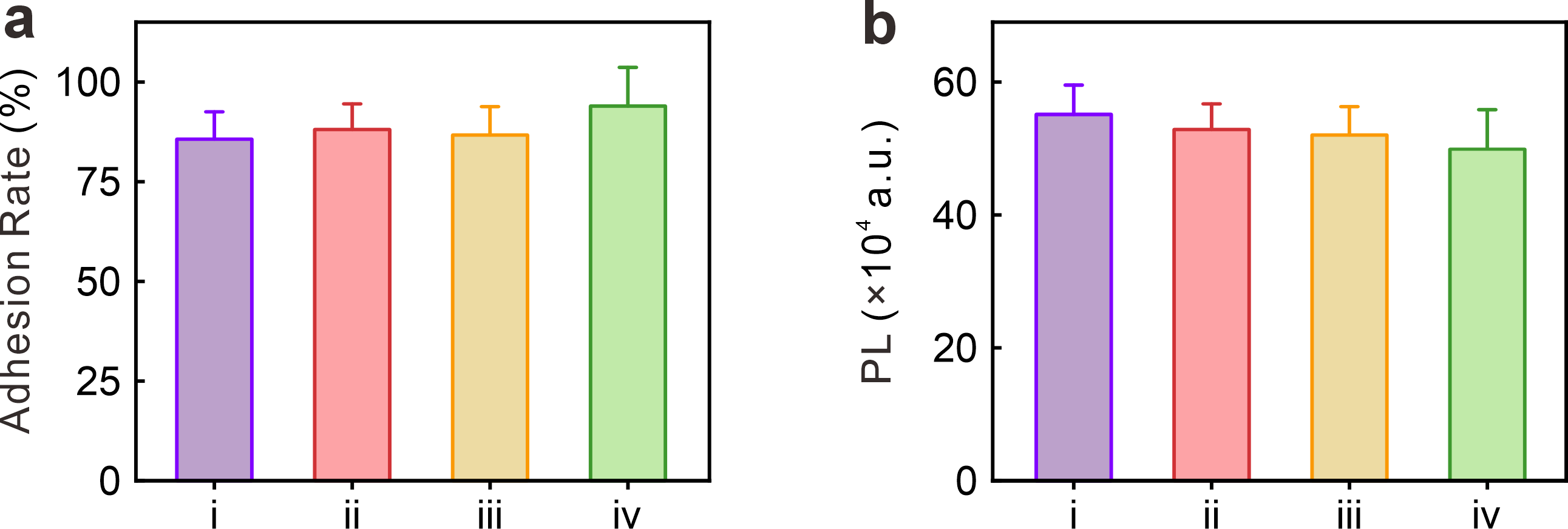


**Figure S28.** **a)** Membrane-maintenance microscopy of HeLa cells under indicated conditions: the blank control (i), co-incubated with 5 μM ZnPC-invasive vesicles for 3 h (ii), under ECL exposure for 2 min without ZnPC (iii), and under ECL exposure for 2 min with 5 μM ZnPC-invasive vesicles (iv). $\lambda$_ex_/$\lambda$_em_ = 622/670 nm. **b)** Adhesion assessments of HeLa cells on electrode surfaces under indicated conditions: the blank control (i), co-incubated with 5 μM ZnPC-invasive vesicles for 3 h (ii), under ECL exposure for 2 min without ZnPC (iii), and under ECL exposure for 2 min with 5 μM ZnPC-invasive vesicles (iv). $\lambda$_ex_/$\lambda$_em_ = 494/515 nm.


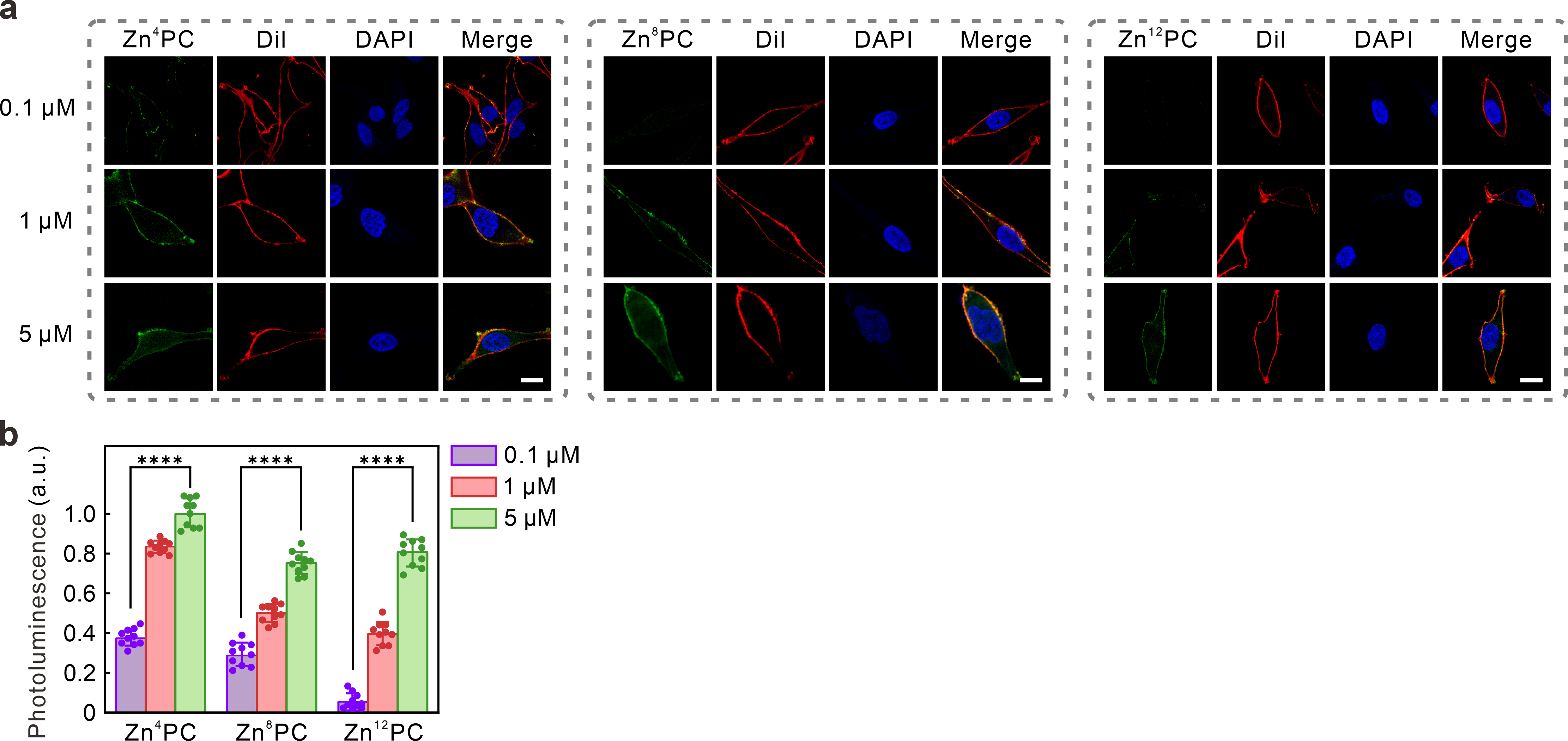


**Figure S29.** **a)** Dosage-dependent delineation of Zn^4^PC (left panel), Zn^8^PC (central), and Zn^12^PC (right) at the administrations of 0.1 μM (top row), 1 μM (middle), and 5 μM (bottom). All ZnPCs were excited with a $\lambda$_ex_ = 410 nm laser across a narrow bandpass filter at $\lambda$_em_ = 640 nm. The dye DAPI (4',6-diamidino-2-phenylindole) was excited with a 360 nm laser. Scale bar: 5 µm. (Nikon, Japan; filter cube: FITC, incident/emergent: 488/525 nm; objective: 63X, Plan Apo, W.D. ∞/0.17, N.A. 1.40, oil immersion; ORCA Flash 4.0 sCMOS camera, 2048×2048 pixel^2^, Hamamatsu Photonics, Japan.) **b)** Histogrammatic harvesting of intracellular fluorescence intensities $I$_PL_ of ZnPC in the Green channel and Dil in the red. Manifested are mean ± standard error from ten individual cells. ^**^*P* < 0.01, ^***^*P* < 0.001, ^****^*P* < 0.0001 (two-tailed Student’s *t*-test).


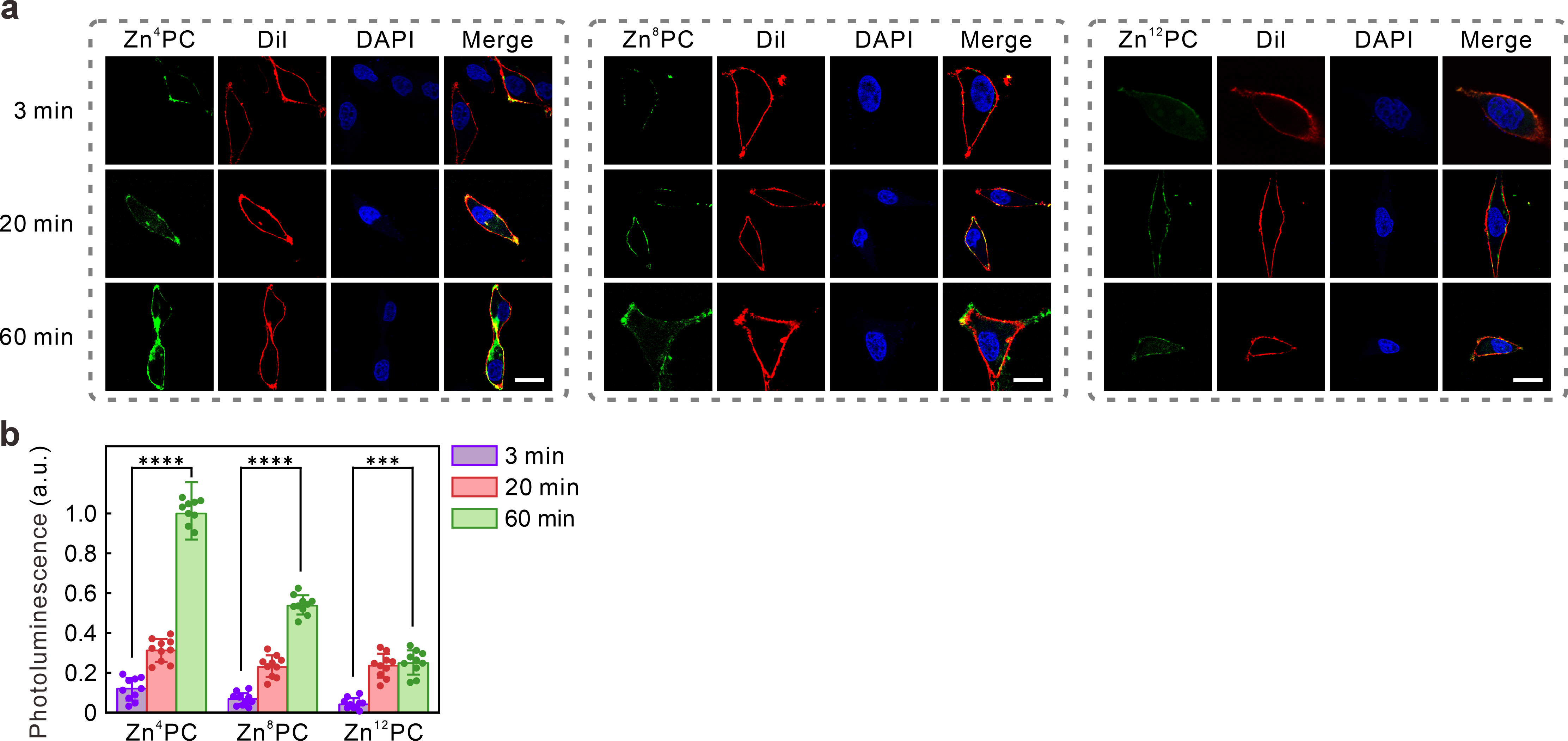


**Figure S30.** **a)** Time-dependent tuning of Zn^4^PC (left panel), Zn^8^PC (central), and Zn^12^PC (right) incubation durations: 3 min (top), 20 min (middle), and 60 min (bottom). All ZnPCs were excited with a $\lambda$_ex_ = 410 nm laser across a narrow bandpass filter at $\lambda$_em_ = 640 nm. DAPI was excited with a 360 nm laser. Scale bar: 5 µm. (Nikon, Japan; filter cube: FITC, incident/emergent: 488/525 nm; objective: 63X, Plan Apo, W.D. ∞/0.17, N.A. 1.40, oil immersion; ORCA Flash 4.0 sCMOS camera, 2048×2048 pixel^2^, Hamamatsu Photonics, Japan.) **b)** Corresponding histogrammatic harvesting of intracellular fluorescence intensities $I$_PL_ of ZnPC in the Green channel and Dil in the red. Manifested are mean ± standard error from ten individual cells. ^**^*P* < 0.01, ^***^*P* < 0.001, ^****^*P* < 0.0001 (two-tailed Student’s *t*-test).


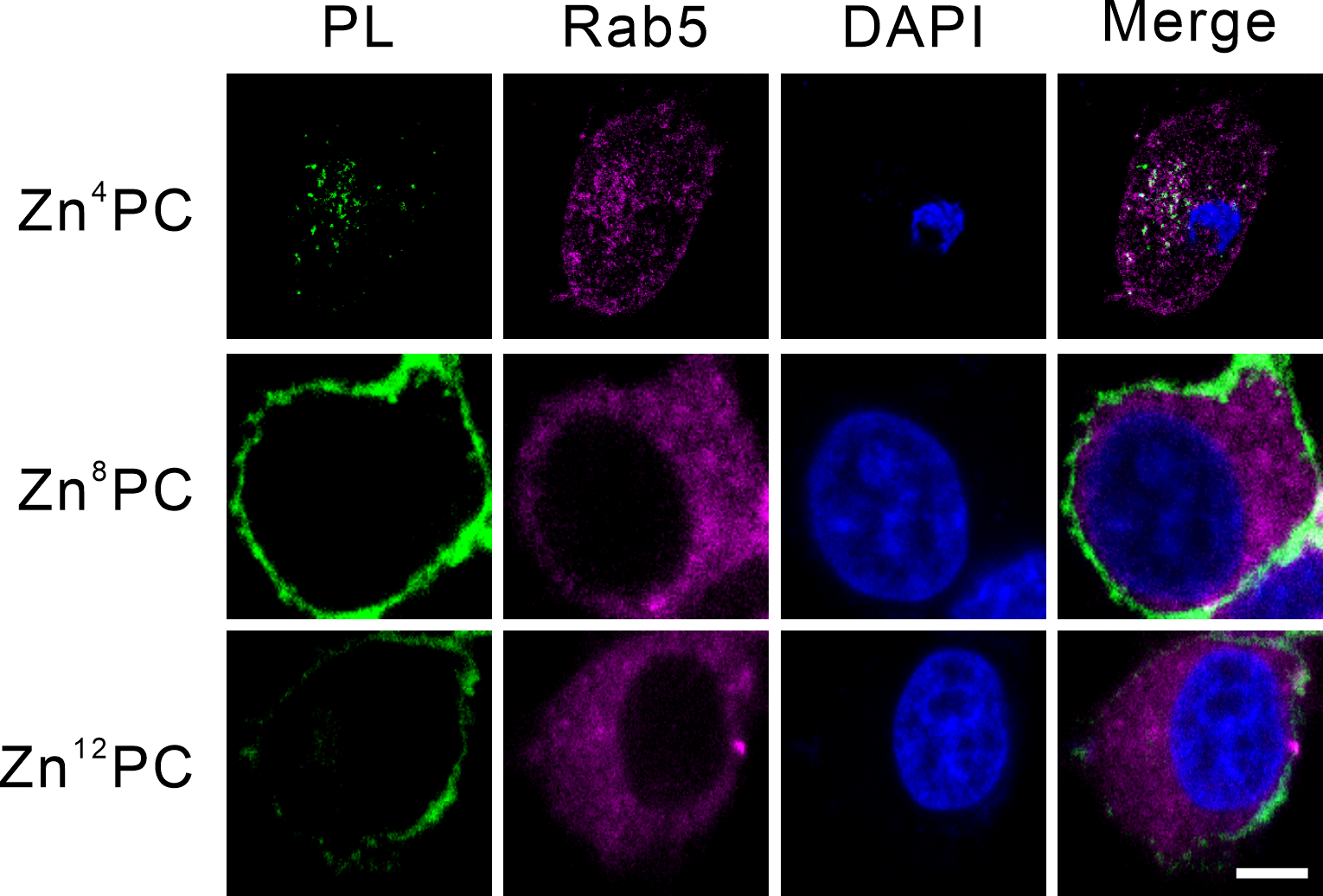


**Figure S31.** Cellular distribution of Zn^4/8/12^PC probes and their spatial relationship with Rab5. All ZnPCs were excited at $\lambda$_ex_ = 410 nm and emitted at $\lambda$_em_ = 640 nm. Rab5 and DAPI were excited at 647 nm and 360 nm, respectively. Scale bar: 5 µm. (Nikon, Japan; filter cube: FITC, incident/emergent: 488/525 nm; objective: 63X, Plan Apo, W.D. ∞/0.17, N.A. 1.40, oil immersion; ORCA Flash 4.0 sCMOS camera, 2048×2048 pixel^2^, Hamamatsu Photonics, Japan.)


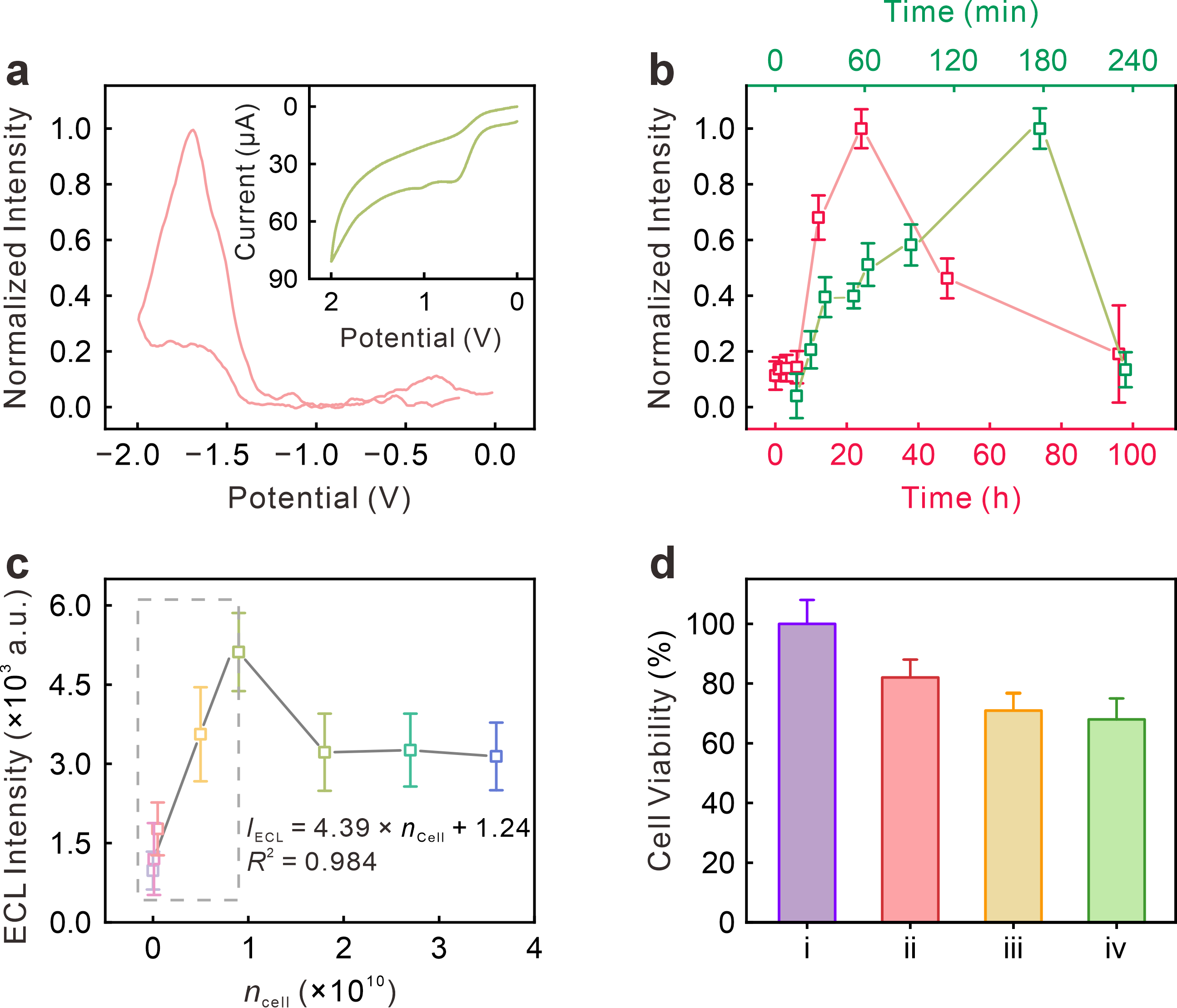


**Figure S32.** **a)** $I$_ECL_-potential trajectories of ZnPC@POPC/Chl containing a content of ZnPC (10 μL×40 μM), scan rate: 0.1 V/s. **Inset:** corresponding CVs. **b)** Melding-mounting optimization of vesicle-cell interaction (24 h was chosen, red) and HeLa cell attachment on GCE (180 min, green). **c)** ECL cytometric calibration of ZnPC-loaded HeLa cells. **Inset:** calibration curve fit $I$_ECL_ = 4.39×$n$[Cell] + 1.24, $R$^2^ = 0.984. **d)** Cytotoxicity comparisons for 10^7^ HeLa cells under conditions of: the blank control (i), co-incubated with 5 μM ZnPC-invasive vesicles for 3 h (ii), under ECL exposure for 2 min without ZnPC (iii), and then under ECL exposure for 2 min with 5 μM ZnPC-invasive vesicles (iv).

**4. Supplementary Tables**

**Table S3.** Condition Configurations for Orthogonal Experimentation

| Group No. | $c$_POPC_  (mg/mL) | $c$_Chol_  (mg/mL) | $c$_ZnPC_  (μM) | ECL Intensity  (a.u.) | Standard  Deviation |
| --- | --- | --- | --- | --- | --- |
| **01** | 0 | 0 | 5 | 1720 | 720 |
| **02** | 0 | 0.8 | 10 | 229 | 248 |
| **03** | 0 | 1.6 | 20 | 156 | 38.6 |
| **04** | 0 | 2.4 | 40 | 20 | 28.3 |
| **05** | 0 | 3.2 | 60 | 0 | 0 |
| **06** | 0.8 | 0 | 10 | 1870 | 955 |
| **07** | 0.8 | 0.8 | 20 | 742 | 250 |
| **08** | 0.8 | 1.6 | 40 | 208 | 222 |
| **09** | 0.8 | 2.4 | 60 | 49 | 59 |
| **10** | 0.8 | 3.2 | 5 | 396 | 201 |
| **11** | 1.6 | 0 | 20 | 3700 | 190 |
| **12** | 1.6 | 0.8 | 40 | 1660 | 1240 |
| **13** | 1.6 | 1.6 | 60 | 522 | 247 |
| **14** | 1.6 | 2.4 | 5 | 782 | 190 |
| **15** | 1.6 | 3.2 | 10 | 768 | 218 |
| **16** | 2.4 | 0 | 40 | 3040 | 2230 |
| **17** | 2.4 | 0.8 | 60 | 2040 | 579 |
| **18** | 2.4 | 1.6 | 5 | 6090 | 1650 |
| **19** | 2.4 | 2.4 | 10 | 2690 | 793 |
| **20** | 2.4 | 3.2 | 20 | 1370 | 736 |
| **21** | 3.2 | 0 | 60 | 2980 | 539 |
| **22** | 3.2 | 0.8 | 5 | 10800 | 2180 |
| **23** | 3.2 | 1.6 | 10 | 9190 | 1950 |
| **24** | 3.2 | 2.4 | 20 | 5430 | 2030 |
| **25** | 3.2 | 3.2 | 40 | 2020 | 303 |

Table S4. Data Derivation and Processing for Orthogonal Experimentation

|  | Factors | | |
| --- | --- | --- | --- |
|  | POPC  (a.u.) | Chol  (a.u.) | ZnPC  (a.u.) |
| $K$_1_ | 2130 | 13300 | 19000 |
| $K$_2_ | 3260 | 15500 | 14700 |
| $K$_3_ | 7430 | 16200 | 11400 |
| $K$_4_ | 15200 | 8970 | 6950 |
| $K$_5_ | 30400 | 4560 | 5590 |
| $k$_1_ | 426 | 2660 | 3800 |
| $k$_2_ | 652 | 3100 | 2940 |
| $k$_3_ | 1486 | 3240 | 2280 |
| $k$_4_ | 3040 | 1790 | 1390 |
| $k$_5_ | 6080 | 912 | 1120 |
| $k$_max_ − $k$_min_ | 5654 | 2328 | 2680 |
| Weight | POPC > ZnPC > Chol | | |
| Optimum | 3.2 mg/mL | 0.8 mg/mL | 0.04 mM |

Table S5. Parametric Profiles of Langmuir Isotherms and Partial Linear Fits

|  | Mg^2+^ | Ca^2+^ |
| --- | --- | --- |
| $r$ (Å) | 0.72 | 1.00 |
| ($I$_ECL_^Θ^ − $I$_ECL_) / $I$_ECL_^Θ^ = $B$_max,1_ × $c$[X^2+^] / ($K$_D1_ + $c$[X^2+^]) + $B$_max,2_ × $c$[X^2+^] / ($K$_D2_ + $c$[X^2+^]) | | |
| $K$_D1_ (mM) | 12.143 ± 5.206 | 14.832 ± 5.005 |
| $K$_D2_ (mM) | 0.021 ± 0.011 | 0.023 ± 0.001 |
| $B$_max,1_ | 0.199 ± 0.035 | 0.285 ± 0.046 |
| $B$_max,2_ | 0.468 ± 0.062 | 0.518 ± 0.004 |
| $R$^2^ | 0.978 | 0.999 |
| ($I$_ECL_^Θ^ − $I$_ECL_) / $I$_ECL_^Θ^ = $k$ × $c$[X^2+^] + $b$ (for the surging segment) | | |
| $k$ (mM^−1^) | 3.104 ± 0.727 | 3.707 ± 1.089 |
| $b$ | 0.068 ± 0.036 | 0.056 ± 0.033 |
| $R$^2^ | 0.949 | 0.959 |
| ($I$_PL_^Θ^ − $I$_PL_) / $I$_PL_^Θ^ = $B$_max,1_ × $c$[X^2+^] / ($K$_D1_ + $c$[X^2+^]) + $B$_max,2_ × $c$[X^2+^] / ($K$_D2_ + $c$[X^2+^]) | | |
| $K$_D1_ (mM) | 4.203 ± 2.492 | 3.318 ± 1.050 |
| $K$_D2_ (mM) | 0.003 ± 0.001 | 0.012 ± 0.003 |
| $B$_max,1_ | 0.238 ± 0.027 | 0.341 ± 0.024 |
| $B$_max,2_ | 0.372 ± 0.056 | 0.456 ± 0.029 |
| $R$^2^ | 0.991 | 0.998 |
| ($R$_ET_^Θ^ − $R$_ET_) / $R$_ET_^Θ^ = $B$_max,1_ × $c$[X^2+^] / ($K$_D1_ + $c$[X^2+^]) + $B$_max,2_ × $c$[X^2+^] / ($K$_D2_ + $c$[X^2+^]) | | |
| $K$_D1_ (mM) | 3.021 ± 0.925 | 2.607 ± 5.296 |
| $K$_D2_ (mM) | 0.000421 ± 0.000137 | 0.000388 ± 0.000103 |
| $B$_max,1_ | 2.387 ± 0.751 | 1.928 ± 0.062 |
| $B$_max,2_ | 3.193 ± 0.361 | 3.342 ± 0.460 |
| $R$^2^ | 0.929 | 0.991 |

**Note:**

$r$: the radius of ion;

$I$_ECL_^Θ^ and $I$_ECL_: the ECL intensity prior to and post the addition of cation at a given concentration, respectively;

$I$_PL_^Θ^ and $I$_PL_: the PL intensity preceding and post the addition of cation at a given concentration, respectively;

$R$_ET_^Θ^ and $R$_ET_: the charge-transfer resistance before and after the addition of cation at a given concentration, respectively;

X^2+^: the general form of divalent cations, $c$[X^2+^]: its concentration;

$K$_D_: the dissociation constant, $B$_max_: the strength at sorptive saturation;

$k$ and $b$: the slope and the intercept of a linear fit, respectively;

$R$^2^: the squared regression coefficient.

Table S6. Piecewise-Parameter Panels for Melittin Measurement

|  | melittin |
| --- | --- |
| ($I$_ECL_^Θ^ − $I$_ECL_) / $I$_ECL_^Θ^ = $k$ × $c$[melittin] + $b$ | |
| Range (μM) | 1~5 |
| $k$ (mM^−1^) | −362.4 ± 26.0 |
| $b$ | 10809.2 ± 78.8 |
| $R$^2^ | 0.975 |
| Range (μM) | 5~100 |
| $k$ (mM^−1^) | −88.0 ± 2.5 |
| $b$ | 9294.5 ± 139.4 |
| $R$^2^ | 0.996 |

**Note:**

$k$: the slope of a linear fit;

$b$: the intercept of a linear fit;

$R$^2^: the squared regression coefficient.

**Table S7.** Wide-Field Whole-Electrode ECL Quantification for Mg^2+^

| $c$[Mg^2+^]  (mM) | ECL Intensity  (a.u.) | Standard  Deviation | Coefficient of Variation |
| --- | --- | --- | --- |
| **0.001** | 185.606 | 36.321 | 0.195689 |
| **0.01** | 130.776 | 27.49 | 0.210207 |
| **0.1** | 111.996 | 22.943 | 0.204856 |
| **1** | 81.034 | 15.469 | 0.190895 |
| **5** | 65.298 | 9.696 | 0.148488 |
| **10** | 49.756 | 7.752 | 0.1558 |
| **15** | 46.439 | 8.749 | 0.188398 |

**Table S8.** Wide-Field Whole-Electrode ECL Quantification for Melittin

| $c$[melittin]  (μM) | ECL Intensity  (a.u.) | Standard  Deviation | Coefficient of Variation |
| --- | --- | --- | --- |
| **1** | 164.463 | 32.839 | 0.199 |
| **2** | 164.691 | 32.570 | 0.197 |
| **3** | 134.536 | 23.062 | 0.171 |
| **4** | 134.556 | 29.016 | 0.215 |
| **5** | 96.677 | 29.355 | 0.303 |
| **10** | 56.531 | 26.677 | 0.471 |
| **25** | 53.386 | 24.130 | 0.451 |
| **50** | 38.095 | 26.855 | 0.704 |
| **75** | 37.385 | 26.950 | 0.720 |
| **100** | 21.476 | 11.581 | 0.539 |

Table S9. Linearized Formulae with Associated $\boldsymbol{R}$^2^ Values

|  | Cu^2+^ | ibuprofen |
| --- | --- | --- |
| ($I$_ECL_^Θ^ − $I$_ECL_) / $I$_ECL_^Θ^ = $k$ × $c$ + $b$ | | |
| $k$ (mM^−1^) | 0.164 ± 0.021 | 0.00026 ± 0.00001 |
| $b$ | 0.051 ± 0.064 | 0.028 ± 0.021 |
| $R$^2^ | 0.951 | 0.991 |
| ($I$_PL_^Θ^ − $I$_PL_) / $I$_PL_^Θ^ = $k$ × $c$ + $b$ | | |
| $k$ (mM^−1^) | 0.179 ± 0.025 |  |
| $b$ | 0.038 ± 0.014 |  |
| $R$^2^ | 0.943 |  |
| Range (mM) | 0.5~5.0 | 0.05~3.00 |

**Table S10.** Wide-Field Whole-Electrode ECL Quantification for Cu^2+^

| $c$[Cu^2+^]  (μM) | ECL Intensity  (a.u.) | Standard  Deviation | Coefficient of Variation |
| --- | --- | --- | --- |
| **0.0** | 108.974 | 31.803 | 0.29184 |
| **0.5** | 64.518 | 34.345 | 0.532332 |
| **1.0** | 59.334 | 20.148 | 0.420938 |
| **2.5** | 52.421 | 22.066 | 0.440718 |
| **5.0** | 9.044 | 22.298 | 2.465502 |

**Table S11.** Wide-Field Whole-Electrode ECL Quantification for Ibuprofen

| $c$[ibuprofen]  (mM) | ECL Intensity  (a.u.) | Standard  Deviation | Coefficient of Variation |
| --- | --- | --- | --- |
| **0.05** | 149.469 | 29.745 | 0.199 |
| **0.1** | 148.601 | 33.110 | 0.222 |
| **0.5** | 147.188 | 31.540 | 0.214 |
| **1.0** | 120.176 | 27.518 | 0.228 |
| **1.5** | 114.121 | 25.272 | 0.219 |
| **2.0** | 80.890 | 17.313 | 0.214 |
| **3.0** | 84.135 | 19.909 | 0.236 |

Table S12. Linearized Formulae with Associated $\boldsymbol{R}$^2^ Values

|  | Aβ_42_ | Tau |
| --- | --- | --- |
| ($I$_ECL_^Θ^ − $I$_ECL_) / $I$_ECL_^Θ^ = $k$ × log$c$ + $b$ | | |
| $k$ (mM^−1^) | −583.1 ± 66.1 | −864.2 ± 106.2 |
| $b$ | 4299.1 ± 137.6 | 5355.1 ± 206.7 |
| $R$^2^ | 0.951 | 0.930 |
| Range (Log ng/mL) | 0.01~10^4^ | 0.01~10^4^ |

Table S13. Analytical-Appraisal Summary for Alzheimer’s Disease Detections

| Method | Biomarkers | Clinical | Detection Limit  (pg/mL) | Reference |
| --- | --- | --- | --- | --- |
| cyclic voltammetry | Aβ_42_ | serum | 266.3 | [27] |
| square wave voltammetry | Aβ_42_ | serum | 5.0 | [28] |
| surface plasmon resonance | Aβ_42_ | plasma | 4.2 | [29] |
| ECL | Aβ_42_ | serum | 11.74 | [30] |
| differential pulse voltammetry | tau | serum | 2.295 | [31] |
| EIS | tau | serum | 140 | [32] |
| ECL | tau | serum | 13.70 | [30] |
| ELISA | Aβ_42_ | / | 9.38 | Commercial Kit  Catalog No. D711115 |
| ELISA | tau | / | 18.75 | Commercial Kit  Catalog No. D711317 |
| ECL | Aβ_42_, tau | serum | 2.25, 3.24 | This Work |

Table S14. Serum-Sample Statistics for $\boldsymbol{c}$[Aβ_42_] and $\boldsymbol{c}$[tau] from Alzheimer’s Patients

| Sample No. | $c$[Aβ_42_] by ECL  (pg/mL) | $c$[Aβ_42_] by ELISA  (pg/mL) | $c$[tau] by ECL  (pg/mL) | $c$[tau] by ELISA  (pg/mL) |
| --- | --- | --- | --- | --- |
| **1** | 30.8 ± 3.8 | 28.3 ± 9.1 | 11.2 ± 2.1 | 13.4 ± 4.2 |
| **2** | 40.5 ± 7.1 | 39.6 ± 8.7 | 8.3 ± 1.7 | 9.2 ± 1.7 |
| **3** | 40.6 ± 1.1 | 44.9 ± 3.5 | 5.2 ± 1.1 | 7.7 ± 2.5 |
| **4** | 51.8 ± 6.2 | 48.4 ± 5.5 | 10.2 ± 3.4 | 11.8 ± 1.6 |
| **5** | 22.4 ± 4.2 | 20.5 ± 8.1 | 9.2 ± 3.1 | 7.3 ± 2.6 |
| **6** | 23.3 ± 6.6 | 18.9 ± 2.3 | 9.8 ± 2.2 | 10.7 ± 1.9 |

**References**

1. A. Aazmi, H. Zhou, Y. Li, M. Yu, X. Xu, Y. Wu, L. Ma, B. Zhang, H. Yang, “Engineered Vasculature for Organ-on-a-Chip Systems,” *Engineering* **2022**, *9*, 131−147. https://doi.org/10.1016/j.eng.2021.06.020
2. H. Yin, Y. Wang, N. Liu, S. Zhong, L. Li, Q. Zhang, Z. Liu, T. Yue, “Advances in the Model Structure of In Vitro Vascularized Organ-on-a-Chip,” *Cyborg Bionic Syst.* **2024**, *5*, 107−117. https://doi.org/10.34133/cbsystems.0107
3. Y. Kim, J. Koo, I.-C. Hwang, R. D. Mukhopadhyay, S. Hong, J. Yoo, A. A. Dar, I. Kim, D. Moon, T. J. Shin, Y. H. Ko, K. Kim, “Rational Design and Construction of Hierarchical Superstructures Using Shape-Persistent Organic Cages: Porphyrin Box-Based Metallosupramolecular Assemblies,” *J. Am. Chem. Soc.* **2018**, *140*, 14547−14551. https://doi.org/10.1021/jacs.8b08030
4. C. Yao, H. Song, Y. Wan, K. Ma, C. Zheng, H. Cui, P. Xin, X. Ji, S. Deng, “Electro-Photodynamic Visualization of Singlet Oxygen Induced by Zinc Porphyrin Modified Microchip in Aqueous Media,” *ACS Appl. Mater. Interfaces* **2016**, *8*, 34833−34843. https://doi.org/10.1021/acsami.6b10213
5. S. Liu, Q. Cheng, T. Wei, X. Yu, L. T. Johnson, L. Farbiak, D. J. Siegwart, “Membrane-Destabilizing Ionizable Phospholipids for Organ-Selective mRNA Delivery and CRISPR−Cas Gene Editing,” *Nat. Mater.* **2021**, *20*, 701−710. https://doi.org/10.1038/s41563-020-00886-0
6. Y. Huang, J. Chen, L. Zhu, K. Ma, K. Kang, M. Yang, S. Lu, M. Yan, Y. Wan, S. Deng, “Electrochemiluminescence-Repurposed Abiological Catalysts in Full Protein Tag for Ultrasensitive Immunoassay,” *Anal. Chem.* **2020**, *92*, 14076−14084. https://doi.org/10.1021/acs.analchem.0c03114
7. K. S. Nair, H. Bajaj, “Advances in Giant Unilamellar Vesicle Preparation Techniques and Applications,” *Adv. Colloid Interface Sci.* **2023**, *318*, 102935. https://doi.org/10.1016/j.cis.2023.102935
8. Y. C. Lin, C. Chipot, S. Scheuring, “Annexin-V Stabilizes Membrane Defects by Inducing Lipid Phase Transition,” *Nat. Commun.* **2020**, *11*, 230. https://doi.org/10.1038/s41467-019-14045-w
9. Y. Lyu, L. M. Becerril, M. Vanzan, S. Corni, M. Cattelan, G. Granozzi, M. Frasconi, P. Rajak, P. Banerjee, R. Ciancio, F. Mancin, P. Scrimin, “The Interaction of Amines with Gold Nanoparticles,” *Adv. Mater.* **2024**, *36*, 2211624. https://doi.org/10.1002/adma.202211624
10. Y. Takei, “Multiplicative Enhancement of Stereoenrichment by a Single Catalyst for Deracemization of Alcohols,” *Science* **2023**, *382*, 780−780. https://doi.org/10.1126/science.adj0040
11. Y. Liu, X. Wang, J. Li, J. Tang, B. Li, Y. Zhang, N. Gu, F. Yang, “Sphingosine 1-Phosphate Liposomes for Targeted Nitric Oxide Delivery to Mediate Anticancer Effects against Brain Glioma Tumors,” *Adv. Mater.* **2021**, *33*, 2101701. https://doi.org/10.1002/adma.202101701
12. S. Lei, J. Zhang, N. T. Blum, M. Li, D.-Y. Zhang, W. Yin, F. Zhao, J. Lin, P. Huang, “In vivo Three-Dimensional Multispectral Photoacoustic Imaging of Dual Enzyme-Driven Cyclic Cascade Reaction for Tumor Catalytic Therapy,” *Nat. Commun.* **2022**, *13*, 1298. https://doi.org/10.1038/s41467-022-29082-1
13. Z. Lei, H. Chen, S. Huang, L. J. Wayment, Q. Xu, W. Zhang, “New Advances in Covalent Network Polymers via Dynamic Covalent Chemistry,” *Chem. Rev.* **2024**, *124*, 7829−7906. https://doi.org/10.1021/acs.chemrev.3c00926
14. Q. Zhang, B. Wei, “Faradaic and Non-Faradaic Self-Discharge Mechanisms in Carbon-Based Electrochemical Capacitors,” *Small* **2025**, *21*, 2311957. https://doi.org/10.1002/smll.202311957
15. J. Chen, Y. Zhao, Y. Wan, L. Zhu, B. Li, J. Wu, L. Li, Y. Huang, Y. Li, X. Long, S. Deng, “Electrochemiluminescent Ion-Channeling Framework for Membrane Binding and Transmembrane Activity Assays,” *Anal. Chem.* **2022**, *94*, 2154−2162. https://doi.org/10.1021/acs.analchem.1c04593
16. G. Pu, Z. Yang, Y. Wu, Z. Wang, Y. Deng, Y. J. Gao, Z. Zhang, X. Lu, “Investigation into the Oxygen-Involved Electrochemiluminescence of Porphyrins and Its Regulation by Peripheral Substituents/Central Metals,” *Anal. Chem.* **2019**, *91*, 2319−2328. https://doi.org/10.1021/acs.analchem.8b05027
17. Y. Huang, J. Chen, L. Zhu, K. Ma, K. Kang, M. Yang, S. Lu, M. Yan, Y. Wan, S. Y. Deng, “Electrochemiluminescence-Repurposed Abiological Catalysts in Full Protein Tag for Ultrasensitive Immunoassay,” *Anal. Chem.* **2020**, *92*, 14076−14084. https://doi.org/10.1021/acs.analchem.0c03114
18. Y. Wu, Z. Han, L. Wei, H. Sun, T. Wang, J. Chen, R. Zhang, X. Lu, “Depolymerization-Induced Electrochemiluminescence of Insoluble Porphyrin in Aqueous Phase,” *Anal. Chem.* **2020**, *92*, 5464−5472. https://doi.org/10.1021/acs.analchem.0c00208
19. S. Hong, Md R. Rohman, J. Jia, Y. Kim, D. Moon, Y. Kim, Y. H. Ko, E. Lee, K. Kim, “Porphyrin Boxes: Rationally Designed Porous Organic Cages,” *Angew. Chem. Int. Ed.* **2015**, *54*, 13241−13244. https://doi.org/10.1002/anie.201505531
20. J. D. Watkins, J. E. Taylor, S. D. Bull, F. Marken, “Mechanistic Aspects of Aldehyde and Imine Electro-Reduction in a Liquid−Liquid Carbon Nanofiber Membrane Microreactor,” *Tetrahedron Lett.* **2012**, *53*, 3357−3360. https://doi.org/10.1016/j.tetlet.2012.04.092
21. F. Boschini, M. Minola, R. Sutarto, E. Schierle, M. Bluschke, S. Das, Y. Yang, M. Michiardi, Y. C. Shao, X. Feng, S. Ono, R. D. Zhong, J. A. Schneeloch, G. D. Gu, E. Weschke, F. He, Y. D. Chuang, B. Keimer, A. Damascelli, A. Frano, E. H. da Silva Neto, “Dynamic Electron Correlations with Charge Order Wavelength along All Directions in the Copper Oxide Plane,” *Nat. Commun.* **2021**, *12*, 597. https://doi.org/10.1038/s41467-020-20824-7
22. R. Filadi, E. Greotti, G. Turacchio, A. Luini, T. Pozzan, P. Pizzo, “Mitofusin 2 Ablation Increases Endoplasmic Reticulum–Mitochondria Coupling,” *Proc. Natl. Acad. Sci.* **2015**, *112*, E2174−E2181 https://doi.org/10.1073/pnas.1504880112
23. Y. Du, Y. Lyu, S. Li, D. Ding, J. Chen, C. Yang, Y. Sun, F. Qu, Z. Xiao, J. Jiang, W. Tan, “Ligand Dilution Analysis Facilitates Aptamer Binding Characterization at the Single-Molecule Level,” *Angew. Chem. Int. Ed.* **2023**, *62*, e202215387. https://doi.org/10.1002/anie.202215387
24. J. S. Aaron, A. B. Taylor, T. L. Chew, “Image Co-Localization-Co-Occurrence Versus Correlation,” *J. Cell Sci.* **2018**, *131*, jcs211847. https://doi.org/10.1242/jcs.211847
25. K. W. Dunn, M. M. Kamocka, J. H. McDonald, “A Practical Guide to Evaluating Colocalization in Biological Microscopy,” *Am. J. Physiol. Cell Physiol.* **2011**, *300*, C723−C742. https://doi.org/10.1152/ajpcell.00462.2010
26. G. Bouchard, W. Zhang, I. Ilerten, I. Li, A. Bhattacharya, Y. Li, W. Trope, J. B. Shrager, C. Kuo, M. G. Ozawa, A. J. Giaccia, L. Tian, S. K. Plevritis, “A Quantitative Spatial Cell-Cell Colocalizations Framework Enabling Comparisons Between in vitro Assembloids and Pathological Specimens,” *Nat. Commun.* **2025**, *16*, 1392. https://doi.org/10.1038/s41467-024-55129-6
27. J. Tong, C. Li, J. Zhao, K. Wang, Z. Zhao, Y. Liu, T. Qing, X. Liu, “Poly-Adenine Assisted Signaling Displaced Probe Ratiometric Electrochemical Aptasensor for Accurate Detection of Alzheimer’s Disease Aβ Biomarkers,” *ACS Appl. Mater. Interfaces* **2024**, *16*, 64297−64306. https://doi.org/10.1021/acsami.4c14877
28. J. Gu, L. Wang, L. Zhao, Y. Zuo, S. Gao, H. Gu, Y. Wang, Y. Yu, “Multiplex Paper-Based Electrochemical Immunosensor for the Simultaneous Monitoring of Blood Biomarkers in Alzheimer's Disease,” *Sens. Actuators. B. Chem.* **2024**, *406*, 135456. https://doi.org/10.1016/j.snb.2024.135456
29. S. Kim, A. W. Wark, H. J. Lee, “Femtomolar Detection of Tau Proteins in Undiluted Plasma Using Surface Plasmon Resonance,” *Anal. Chem.* **2016**, *88*, 7793−7799. https://doi.org/10.1021/acs.analchem.6b01825
30. Y. L. Jia, X. Q. Li, H. Y. Chen, W. Zhao, J. J. Xu, “Simultaneous Detection of Alzheimer's Biomarkers Using a Visual Electrochemiluminescence Bipolar Array,” *Sens. Actuators. B. Chem.* **2023**, *396*, 134591. https://doi.org/10.1016/j.snb.2023.134591
31. A. Shiravandi, F. Yari, N. Tofigh, M. Kazemi Ashtiani, K. Shahpasand, M. H. Ghanian, F. Shekari, F. Faridbod, “Earlier Detection of Alzheimer’s Disease Based on a Novel Biomarker cis P-tau by a Label-Free Electrochemical Immunosensor,” *Biosensors* **2022**, *12*, 879. https://doi.org/10.3390/bios12100879
32. H. T. Le, S. Cho, “Sensitive Electrochemical Detection of Phosphorylated-Tau Threonine 231 in Human Serum Using Interdigitated Wave-Shaped Electrode,” *Biomedicines* **2021**, *10*, 10. https://doi.org/10.3390/biomedicines10010010
